# Design, synthesis, and multi-target anticancer evaluation of 1,3-thiazolodin-4-one analogues against breast cancer: mechanistic insights into estrogen metabolism, inflammation, angiogenesis, and oxidative stress

**DOI:** 10.1039/d5ra07680c

**Published:** 2026-01-12

**Authors:** Manar A. El-Zend, Ibrahim M. El-Deen, Mohamed Fathy Mohamed Reyad, Samar Zuhair Alshawwa, Amal Abdullah Alrashidi, Essa M. Saied

**Affiliations:** a Department of Chemistry, Faculty of Science, Port Said University Port Said Egypt m.elzend@sci.psu.edu.eg ieldeen@yahoo.com mohamedfathyreyad@gmail.com; b Department of Pharmaceutical Sciences, College of Pharmacy, Princess Nourah bint Abdulrahman University P.O. Box 84428 Riyadh 11671 Saudi Arabia SZAlshawwa@pnu.edu.sa aaalrashidi@pnu.edu.sa; c Chemistry Department, Faculty of Science, Suez Canal University Ismailia 41522 Egypt essa.saied@science.suez.edu.eg; d Institute for Chemistry, Humboldt Universität zu Berlin 12489 Berlin Germany

## Abstract

Breast cancer remains a leading cause of mortality in women, underscoring the need for multitarget therapeutic agents. A series of 2,3-disubstituted-1,3-thiazol-4-one derivatives was synthesized and characterized, and their antiproliferative activity was assessed against MDA-MB-231 and MCF-7 cells. Compound 6 was the most active analogue, showing IC_50_ values of 2.25 ± 0.18 µM and 6.70 ± 0.63 µM, respectively, with superior selectivity and potency compared with doxorubicin. Mechanistic studies demonstrated that compound 6 induced G_0_/G_1_ arrest and apoptosis, supported by caspase-3/7 activation. It also inhibited key enzymes in estrogen biosynthesis, including aromatase (IC_50_ = 38.3 ± 2.3 nM) and steroid sulfatase (IC_50_ = 12.7 ± 0.76 µM), and selectively suppressed COX-2 (IC_50_ = 5.38 ± 0.18 µM; SI = 10.44). Strong antioxidant activity (DPPH IC_50_ = 16.26 ± 0.6 µM) further contributed to its pharmacological profile. *In vivo*, compound 6 significantly reduced tumor load in the Ehrlich ascites carcinoma model and improved liver, kidney, oxidative stress, and histopathological markers. It also lowered circulating TNF-α and VEGFR-II, indicating additional anti-inflammatory and anti-angiogenic effects. *In silico* toxicity profiling predicted a favorable safety profile, with no Ames mutagenicity, no hERG inhibition, no skin sensitization, low acute/chronic toxicity, and no predicted CYP450 inhibition. ProTox-III classified compound 6 as inactive toward major organ-toxicity endpoints. Computational studies supported these results: docking and 100-ns MD simulations showed stable binding to aromatase, STS, COX-2, TNF-α, and VEGFR-II. PCA and free-energy landscape analyses revealed early conformational adjustments followed by convergence into compact, low-energy states, consistent with stable ligand–protein interactions. Overall, compound 6 emerges as a promising multitarget lead integrating cytotoxic, hormone-modulatory, anti-inflammatory, antioxidant, and anti-angiogenic activities for potential breast cancer therapy.

## Abstract 2

Breast cancer remains a leading cause of mortality in women, underscoring the need for multitarget therapeutic agents.

## Graphical abstract

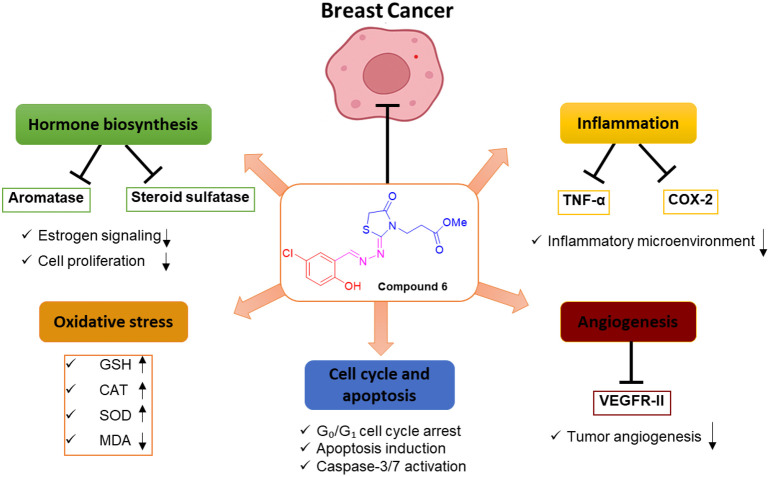

## 1 Introduction

Breast cancer is the most frequently diagnosed malignancy among women and a leading cause of cancer-related deaths worldwide. Despite decades of research and advances in chemotherapy, endocrine therapy, and targeted treatment, breast cancer remains difficult to cure.^1,2^ A major challenge in breast cancer treatment is the disease's complexity, including dysregulated hormone signaling, oxidative stress imbalance, chronic inflammation, sustained angiogenesis, and evasion of apoptosis.^3^ Resistance to apoptosis allows cancer cells to survive despite DNA damage and therapeutic stress. Alterations in the BCL-2 protein family, with overexpression of anti-apoptotic members (*e.g.*, BCL-2, BCL-xL) and downregulation of pro-apoptotic counterparts (*e.g.*, BAX, PUMA), contribute to apoptosis evasion in breast cancer. Targeting these survival mechanisms to restore apoptotic sensitivity remains a critical aspect of anticancer drug development.^4,5^ In hormone-dependent breast cancers, which account for nearly 75% of cases, enzymes of local estrogen biosynthesis play a pivotal role.^6^ Steroid sulfatase (STS) catalyzes the hydrolysis of inactive sulfated estrogens into active hormones, while aromatase (CYP19) mediates the final step in estrogen synthesis from androgens. Overexpression of these enzymes sustains intratumoral estrogen levels, driving tumor growth and therapy resistance. Consequently, dual inhibition of STS and aromatase represents a compelling strategy to suppress estrogen-driven tumor progression.^7,8^ Beyond hormone dependence, oxidative stress is another hallmark of breast cancer biology. Tumor cells exhibit elevated levels of reactive oxygen species (ROS) due to their high metabolic demand and hypoxic microenvironment. While low-to-moderate ROS levels promote oncogenic signaling, DNA mutations, and genomic instability, excessive ROS accumulation can trigger apoptosis.^9^ To survive, cancer cells develop robust antioxidant defenses, upregulating systems such as glutathione (GSH), catalase (CAT), and superoxide dismutase (SOD). This balance allows tumor cells to exploit ROS signaling while avoiding lethal oxidative damage. Modulating this redox state offers an attractive therapeutic opportunity.^10,11^ Closely linked to oxidative stress is inflammation, which contributes to the formation of a tumor-supportive microenvironment. Cyclooxygenase-2 (COX-2) is frequently overexpressed in breast tumors and plays a central role in the production of prostaglandins, which drive proliferation, angiogenesis, and metastasis. Elevated COX-2 expression has been correlated with poor prognosis, while its inhibition has demonstrated chemopreventive potential.^12^ Similarly, the pro-inflammatory cytokine tumor necrosis factor-alpha (TNF-α) enhances cancer progression by activating survival signaling pathways (*e.g.*, NF-κB) and promoting immune evasion.^13^ Angiogenesis is another critical hallmark sustaining tumor growth and metastasis. This process is largely orchestrated by the vascular endothelial growth factor (VEGF), which stimulates endothelial proliferation and vessel formation. High VEGF expression correlates with aggressive tumor behavior and poor clinical outcomes. Therefore, strategies that combine anti-angiogenic activity with modulation of other tumor hallmarks hold greater promise.^14,15^

**Fig. 1:**
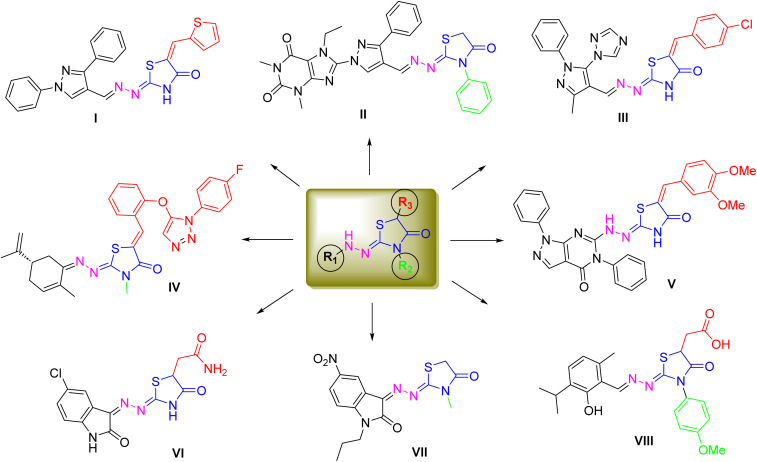
Representative structure of recently developed 2-hydrazon-4-thiazolodine analogues with multi-target activity.

Collectively, these insights emphasize the need for multi-target agents capable of simultaneously modulating several cancer-promoting processes (polypharmacology).^16,17^ Recently, thiazolidin-4-ones-based compounds (1,3-thiazol-4-ones) have emerged as privileged heterocycles with diverse pharmacological profiles, including antioxidants, antimicrobial, anti-inflammatory, and anticancer activities. Among reported thiazolidin-4-ones, 2-hydrazono-thiazolidinones demonstrated substantial anticancer activity through their ability to induce cell cycle arrest, disrupt mitochondrial function, trigger apoptosis, and inhibit angiogenesis. Structural modifications at the 3-, and 5-positions of 2-hydrazone-thiazolidinone core are particularly effective in fine-tuning biological activity. Recent advances in rational drug design have yielded thiazolone hybrids that combine the thiazolone scaffold with complementary pharmacophores, thereby enhancing multi-target engagement and therapeutic efficacy (Fig. 1).^18–21^ For example, derivatives incorporating a 2-pyrazolyl-hydrazono moiety have shown wide-ranging pharmacological potential. Compounds I–III were reported to exhibit antioxidant, anti-inflammatory, and antiproliferative activities. Among them, compound I displayed superior anti-inflammatory activity compared to reference drugs diclofenac and celecoxib,^22^ while the purine–pyrazole analogue (compound II) demonstrated remarkable antiproliferative activity against PC3, Caco-2, HepG-2, MCF-7, and A549 cells (IC_50_: 18.5–23.4 µM), significantly outperforming fluorouracil (IC_50_ > 80 µM).^23^ Notably, the arylidene-substituted derivative (compound III) achieved potent cytotoxicity toward MCF-7 cells (IC_50_ = 5.4 µM), even exceeding doxorubicin.^24^

Further diversification of the scaffold has also yielded promising outcomes. Oubella *et al.* synthesized novel 2-triazolyl-hydrazono-thiazolodines (compound IV), which exhibited potent cytotoxic activity against HT-1080 and A-549 cells with IC_50_ values of 17.1 and 15.4 µM, respectively.^25^ In another study, Labouta *et al.* reported two series of 2-pyrazolo-pyridine-hydrazono-thiazolodines, of which compound V demonstrated the strongest anti-inflammatory activity by selectively inhibiting COX-2 (IC_50_ = 0.37 µM).^26^ The thiazolone framework has also been explored for antioxidant and antimicrobial potential. Asghari *et al.* described 2-indolyl-hydrazono-thiazolodines (compound VI), which displayed substantial antioxidant capacity alongside broad-spectrum antibacterial activity.^27^ Moreover, Yousef *et al.* identified a related 2-indolyl analogue (compound VII) with strong antiproliferative activity toward HepG2, MCF-7, and HT-29 cells (IC_50_: 3.3–5.3 µM).^28^ Recently, Chen *et al.* reported that a 2-isopropylphenol-hydrazono-thiazolodine derivative (compound VIII) exerts anticancer activity by targeting the oncogenic kinase PIM-1 (proviral integration site for Moloney murine leukemia virus kinase 1).^29^ In light of these attributes and our interest in discovering novel bioactive compounds,^30–35^ the present study aimed to design, synthesize, and investigate novel 2-hydrazone-, 3-substituted-1,3-thiazol-4-one derivatives as potential multi-target agents against breast cancer. Through a combination of *in vitro*, *in vivo*, and *in silico* approaches, we evaluated their effects on estrogen biosynthesis (STS, aromatase), oxidative stress regulation, inflammatory mediators (COX-2, TNF-α), angiogenesis (VEGFR-2), and apoptosis. By integrating molecular, cellular, and computational evidence, this work aimed to establish thiazolone derivatives as promising candidates for the development of multi-target therapeutics to combat breast cancer's complexity and resistance.

## 2 Results and discussions

### 2.1 Chemistry

**Scheme 1:**
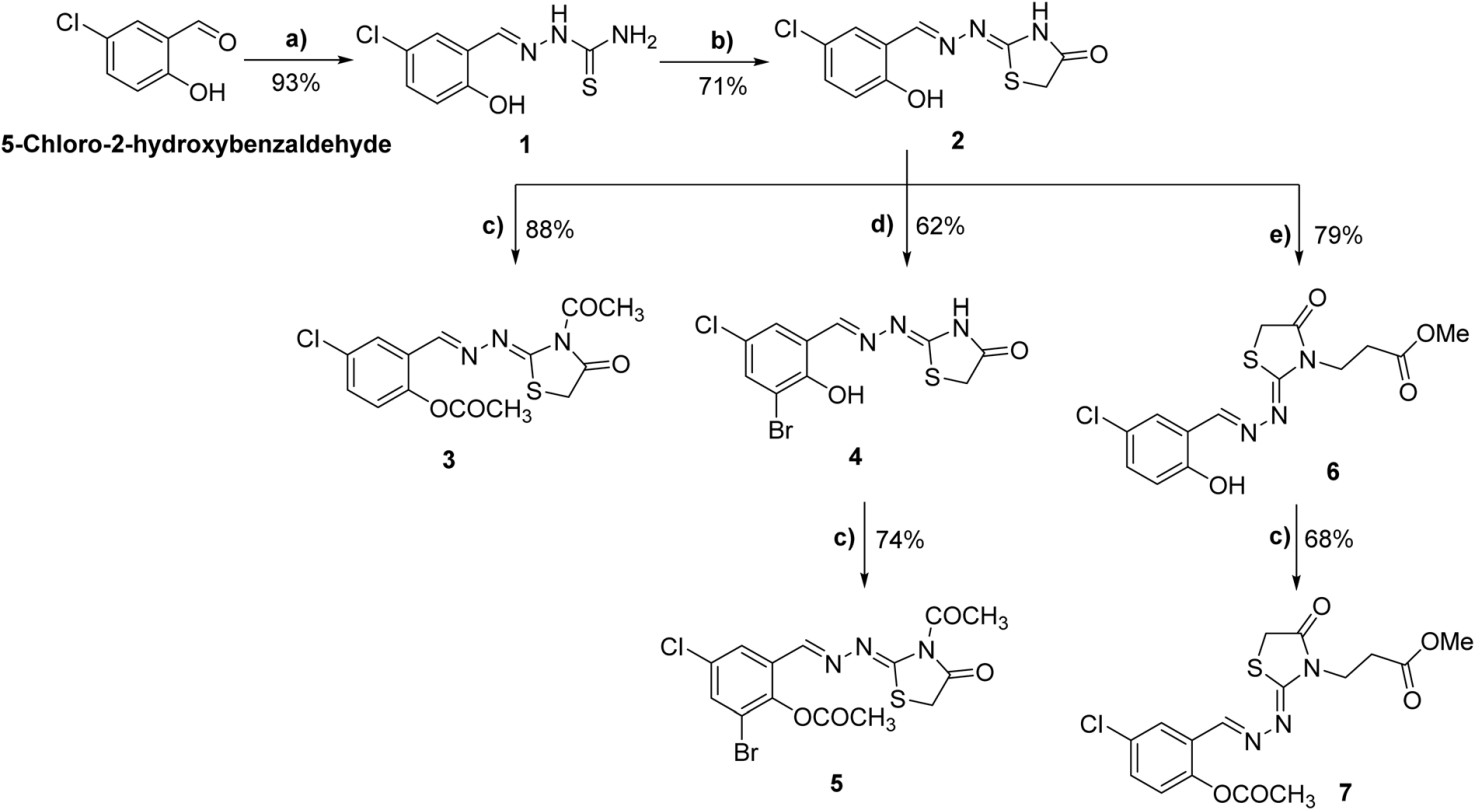
Synthesis of novel 1,3-thiazolidin-4-one analogues 2–7. Reagents and conditions: (a) NH_2_NHCSNH_2_, AcOH, EtOH, 85 °C, 16 h; (b) ClCH_2_COOEt, K_2_CO_3_, EtOH, reflux, 85 °C, 12 h; (c) acetic anhydride, reflux, 150 °C, 12–18 h; (d) Br_2_, AcOH, 60 °C-r. t., 16 h; (e) methyl acrylate, Et_3_N, DMF, reflux, 155 °C, 14 h.

The synthesis of the envisioned class of 2,3-disubstituted-1,3-thiazol-4-one analogues (2–7) started from commercially available 5-chloro-2-hydroxybenzaldehyde. As depicted in Scheme 1, the acid-catalyzed condensation of substituted benzaldehyde with hydrazinecarbothioamide provided the corresponding thiosemicarbazone 1 in quantitative yield.^36,37^ Treatment of thiosemicarbazone 1 with ethyl chloroacetate mediated by anhydrous K_2_CO_3_ furnished the key intermediate hydrazineylidene-thiazolidin-4-one 2 in satisfactory yield (71%). Having thiazolidin-4-one 2 in hand, we aimed to explore the different structural features in the scaffold, including hydrazineylidene and thiazolidin-4-one moieties. Toward this, thiazolidin-4-one 2 was initially reacted with acetic anhydride under reflux to yield the corresponding diacetylated hydrazineylidene-thiazolidin-4-one 3 in an 88% yield. Furthermore, the bromination of hydrazineylidene moiety was successfully achieved by treatment of thiazolidin-4-one 2 with bromine in the presence of glacial acetic acid to yield the 3-bromo-5-chloro-2-hydroxybenzylidene-hydrazineyl-thiazol-4-one 4 in a good yield (62%). Subsequently, compound 4 reacted with acetic anhydride to provide the corresponding diacetylated 3-bromo-5-chloro-2-hydroxybenzylidene-hydrazineyl-thiazol-4-one 5 in 74% yield. Finally, we explored the thiazolidin-4-one moiety at position 3 by subjecting the hydrazineylidene-thiazolidin-4-one 2 to a condensation reaction with methyl acrylate in the presence of triethylamine to give methyl-3-(2-(5-chloro-2-hydroxybenzylidene)-1-(4-oxo-thiazol-2-yl)-hydrazineyl)propanoate 6 in 79% yield. The reaction of compound 6 with acetic anhydride under standard conditions provided the corresponding diacetylated methyl-3-(2-(5-chloro-2-hydroxybenzylidene)-1-(4-oxo-thiazol-2-yl)hydrazineyl)-propanoate 7 in a satisfactory yield (68%). The synthesized compounds were characterized by ^1^H NMR, ^13^C NMR, FTIR, and elemental analyses. For compound 2, the IR spectrum showed bands at 3450–3390 cm^−1^ (phenolic O–H), 3226 cm^−1^ (N–H), 1705 cm^−1^ (lactam C=O), 1633 cm^−1^ (C=N), and 1605–1589 cm^−1^ (C=C), consistent with a thiazolidinone scaffold bearing phenolic and imine functionalities. In the ^1^H-NMR, the OH at *δ* 12.10 and NH at *δ* 10.95 appeared as broad singlets, confirming hydrogen-bonded protons. The azomethine proton was observed at *δ* 8.61, while aromatic signals appeared as *δ* 7.66, *δ* 7.34–7.37, and *δ* 6.97–6.99, consistent with a trisubstituted benzene. The methylene of the thiazolidinone ring resonated as a singlet at *δ* 3.98. The ^13^C-NMR supported this, with the lactam carbonyl at *δ* 178.3, a second carbonyl/imine carbon at *δ* 166.0, azomethine at *δ* 157.1, phenolic C–O at *δ* 156.0, and aromatic carbons at *δ* 137.8, 132.0, 130.8, 128.8, and 125.9. The methylene group appeared at *δ* 33.9, confirming the presence of the thiazolidinone ring system. The IR for compound 3 displayed strong ester and lactam carbonyl bands at 1753–1748 cm^−1^ and 1713 cm^−1^, with additional C=N absorption at 1633 cm^−1^. In the ^1^H-NMR, *δ* 8.56 corresponded to the azomethine proton, while aromatic protons appeared at *δ* 7.70, 6.96–6.98, and 6.73–6.78, indicating a trisubstituted aromatic system. The methylene of the thiazolidinone appeared at *δ* 3.94, and two methyl singlets at *δ* 1.91 and 1.78 corresponded to acetyl substituents. The ^13^C-NMR spectrum confirmed three carbonyl carbons at *δ* 176.9, 172.2, and 169.2, while *δ* 166.0 and 163.5 represented imine and C–O carbons. Aromatic/olefinic carbons resonated at *δ* 157.1, 154.9, 152.4, 147.9, 130.6, and 129.3, while the methylene was found at *δ* 34.5 and the methyl carbons at *δ* 22.9 and 21.6.

For compound 4, IR absorptions at 3485–3405 cm^−1^ (O–H), 3222 cm^−1^ (N–H), and 1711 cm^−1^ (C=O) confirmed hydroxyl, amine, and lactam groups. In the ^1^H-NMR, *δ* 11.00, *δ* 10.86, and *δ* 8.47 supported the presence of phenolic and imine functionalities. Aromatic protons appeared at *δ* 7.90 and 7.59, while *δ* 3.90 indicated the thiazolidinone CH_2_. In the ^13^C-NMR, *δ* 181.3 and 180.1 corresponded to lactam carbons, *δ* 172.5 to the azomethine carbon, and *δ* 170.3 to phenolic C–O. Aromatic carbons resonated at *δ* 152.4, 151.5, 144.8, 138.4, and 135.4, while the methylene carbon was downfield at *δ* 21.5. The IR spectrum for compound 5 showed carbonyl stretches at 1701, 167.2, and 158.6 cm^−1^, consistent with the presence of multiple carbonyl groups. In the ^1^H-NMR, the azomethine appeared at *δ* 9.04, aromatic protons at *δ* 7.00–7.25, thiazolidinone methylene at *δ* 4.55, and acetyl methyls at *δ* 1.77 and 1.24. The ^13^C-NMR confirmed carbonyl carbons at *δ* 173.3, 172.4, and 167.2, imine at *δ* 157.7, phenolic C–O at *δ* 149.6, aromatic carbons between *δ* 134.9–126.2, the methylene at *δ* 36.1, and methyl carbons at *δ* 31.3 and 24.8. For compound 6, the IR bands at 3425 cm^−1^ (O–H), 1690 cm^−1^ (C=O), and 1615 cm^−1^ (C=N) were consistent with phenolic and imine groups. The ^1^H-NMR revealed OH (*δ* 10.88), CH=N (*δ* 8.69), aromatic protons at *δ* 7.68, 7.36–7.39, and 6.97–7.00, and the thiazolidinone methylene at *δ* 4.03. Additional resonances at *δ* 3.95–3.99 (CH_2_), *δ* 3.36 (OCH_3_), and *δ* 2.70–2.74 (CH_2_) confirmed a methoxyalkyl substituent. The ^13^C-NMR displayed carbonyl carbons at *δ* 174.1 and 164.8, imine at *δ* 163.0, phenolic C–O at *δ* 157.1, aromatic carbons between *δ* 132.3–118.8, methoxy at *δ* 60.0, and methylenes at *δ* 34.0, 32.9, and 31.6. Finally, the IR spectra of compound 7 showed carbonyl absorptions at 1680 cm^−1^, imine at 1610 cm^−1^, and aromatic C=C bands at 1600–1570 cm^−1^. In the ^1^H-NMR, the CH=N appeared at *δ* 8.66, aromatic protons at *δ* 7.65, 7.28–7.30, and 6.98–7.00, and the thiazolidinone methylene at *δ* 3.99. Additional peaks at *δ* 3.87–3.97 and *δ* 2.70–2.71 confirmed methylene groups, *δ* 3.40 supported a methoxy substituent, and *δ* 1.76 indicated a methyl group. The ^13^C-NMR confirmed these assignments with carbonyl signals at *δ* 177.4, 174.1, 172.8, and 171.5, aromatic carbons at *δ* 164.2–119.3, methoxy at *δ* 63.2, methylenes at *δ* 32.8 and 31.6, and methyl at *δ* 21.5. Altogether, the spectral data for compounds 2–7 are fully consistent with the proposed thiazolidinone derivatives, where every diagnostic IR, ^1^H, and ^13^C signal is accounted for and matches the expected substitution pattern.

### 2.2 Assessment of cytotoxic activity and exploration of multi-targeted mode of action

#### 2.2.1. Evaluation of antiproliferative activity

**Fig. 2:**
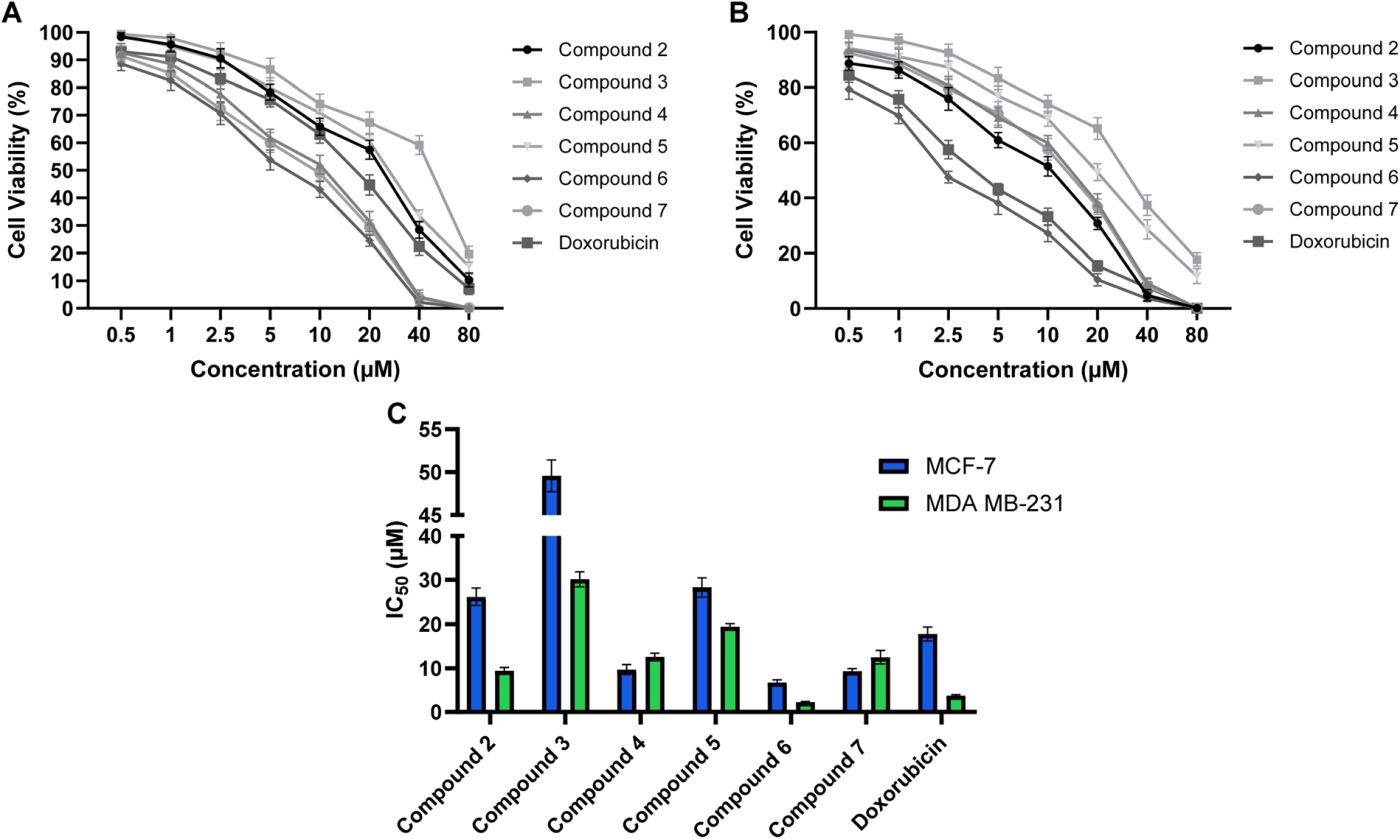
Effect of synthesized compounds (2–7) against human breast cancer MCF-7 and MDA-MB-231 cells as assessed by MTT assay after incubation for 48 h at different concentrations. (A and B) The dose-dependent curves of compounds 2–7 and doxorubicin toward the cellular viability of MCF-7 (A) and MDA-MB-231 (B) cells. (C) Summary of the IC_50_ values for the cytotoxic activity of compounds 2–7 and doxorubicin against MDA-MB-231and MCF-7 cells. Data expressed as mean ± SE from three independent experiments (*n* = 3).

Having compounds 2–7 in hand, we investigated their cytotoxic potential at various concentrations against MDA-MB-231 and MCF-7 breast cancer cell viability using the MTT assay. The activity of tested compounds was compared with doxorubicin as a reference drug in our assessments, and the inhibitory concentration at 50% inhibition was assessed from the dose-dependent curve. The 1,3-thiazolidin-4-one derivatives 2–7 demonstrated considerable and dose-dependent antiproliferative activity against the examined MDA-MB-231 and MCF-7 cell lines (Fig. 2 and Table S1). Analysis of structural features correlated to antiproliferative activity indicated that substitution at both position-3 of 1,3-thiazolidin-4-one scaffold and benzylidene moiety of 4-chloro-2-(hydrazineylidenemethyl)phenol scaffold are key players for the compound activity. Toward MDA MB-231 cell viability assessment, the main 1,3-thiazolidin-4-one scaffold 2 displayed a substantial inhibitory activity with an IC_50_ of 9.41 ± 0.75 µM. Acetylation of the scaffold at both the phenolic and thiazolidin-4-one positions resulted in a significant reduction in activity, as indicated in compound 3 (IC_50_ = 30.16 ± 1.73 µM). Bromination of the benzylidene moiety at position 3 showed a non-significant effect on the activity of the compound (compound 4, IC_50_ = 12.55 ± 0.87 µM). Similarly, acetylation of compound 4 at phenolic and thiazolidin-4-one positions led to attenuation in activity (compound 5, IC_50_ = 19.44 ± 0.73 µM). Interestingly, the substitution at position 3 in the 1,3-thiazolidin-4-one moiety with methyl propionate substantially elevated the cytotoxic activity of the compound, as shown in compound 6 (IC_50_ = 2.25 ± 0.18 µM). Further, acetylation of the phenolic-OH in compound 6 resulted in a significant amelioration in activity (compound 7, IC_50_ = 12.47 ± 1.55 µM), affirming the role of phenolic-OH in the cytotoxic activity of this class of compounds. Toward MCF-7 cell viability, the parent 1,3-thiazolidin-4-one scaffold 2 showed a moderate inhibitory activity with an IC_50_ of 26.21 ± 1.93 µM. Similar to the trend observed for MDA-MB-231 cells, acetylation at both the phenolic and thiazolidin-4-one positions (compound 3) resulted in a pronounced decline in cytotoxic activity (IC_50_ = 49.59 ± 1.85 µM), highlighting the unfavorable impact of masking the free hydroxyl group. Interestingly, bromination of the benzylidene moiety at position 3 markedly improved the cytotoxic potency compared to compound 2, as evidenced in compound 4 (IC_50_ = 9.67 ± 1.14 µM). However, acetylation of this brominated analogue (compound 5) led to a significant loss of activity (IC_50_ = 28.33 ± 2.16 µM), further confirming the importance of the free phenolic group for optimal activity. Substitution at position 3 of the thiazolidin-4-one ring with a methyl propionate group (compound 6) conferred the most pronounced activity in this series against MCF-7 cells, with an IC_50_ of 6.70 ± 0.63 µM, representing a ∼4-fold enhancement over the parent scaffold. Acetylation of the phenolic hydroxyl in this highly potent analogue (compound 7) resulted in a moderate reduction in activity (IC_50_ = 9.28 ± 0.62 µM). These findings indicate that, despite generally reduced potency of the compounds against MCF-7 compared to MDA-MB-231 cells, the structure–activity relationships for both cell lines are largely consistent, with the presence of a free phenolic hydroxyl and specific substitutions at the thiazolidin-4-one and benzylidene moieties being critical determinants of cytotoxic activity. Substitution on the aromatic ring significantly influenced activity: analogues bearing electron-donating groups showed enhanced potency, whereas electron-withdrawing substituents led to a marked reduction in cytotoxicity. Increasing hydrophobicity and steric bulk generally improved activity, indicating that more hydrophobic substituents may facilitate superior membrane penetration or stronger hydrophobic interactions within cellular targets. Among the series, compound 6 exhibited the highest potency, suggesting that the presence of the heteroaryl moiety at position 2 provides an optimal balance of steric, electronic, and hydrophobic contributions for interaction with intracellular targets. In contrast, compounds lacking this feature or bearing less favorable electronic profiles showed weaker activity. These SAR trends demonstrate that both electronic nature and steric characteristics of the substituents on the thiazolidinone scaffold strongly modulate the antiproliferative effects. These findings are in alignment with previous reports, which showed that 1,3-thiazolidin-4-one analogues exhibit substantial antiproliferative activity toward breast cancer cells.^38–40^

Among the examined compounds, compound 6 exhibited the most potent antiproliferative activity, with IC_50_ values of 2.25 ± 0.18 µM against MDA-MB-231 cells and 6.70 ± 0.63 µM against MCF-7 cells. Notably, its cytotoxic effect surpassed that of the reference drug doxorubicin, which displayed IC_50_ values of 3.72 ± 0.24 µM and 17.77 ± 1.57 µM, respectively, under the same experimental conditions (Table S1). Based on these findings, compound 6 was selected to examine the selectivity of this class of compounds toward breast cancer cells. Toward this, the cytotoxic activity of compound 6 was assessed against the non-tumorigenic MCF-10A breast epithelial cell line in comparison with doxorubicin. Compound 6 exhibited antiproliferative activity with an IC_50_ of 36.57 ± 1.37 µM, whereas doxorubicin exhibited a markedly lower IC_50_ of 14.22 ± 0.53 µM under the same experimental conditions (Fig. S7). The resulting selectivity index (SI) values for compound 6 were 16.2 against MDA-MB-231 cells and 5.5 against MCF-7 cells, indicating a substantially higher preference for targeting cancer cells over normal cells. Under the same conditions, doxorubicin exhibited SI values of 3.8 for MDA-MB-231 and 0.8 for MCF-7 cells (IC_50_ values of 3.72 ± 0.24 µM and 17.77 ± 1.57 µM *vs.* 14.22 ± 0.53 µM for MCF-10A), confirming that compound 6 displays markedly superior selectivity toward cancer cells compared with the reference drug. Collectively, our investigations indicated that compound 6 emerged as the lead analogue, surpassing the reference drug doxorubicin in potency for both cell lines, underscoring its potential as a promising candidate for further anticancer evaluation.

#### 2.2.2. Exploration of the *in vitro* antiproliferative mechanism of action

**Fig. 3:**
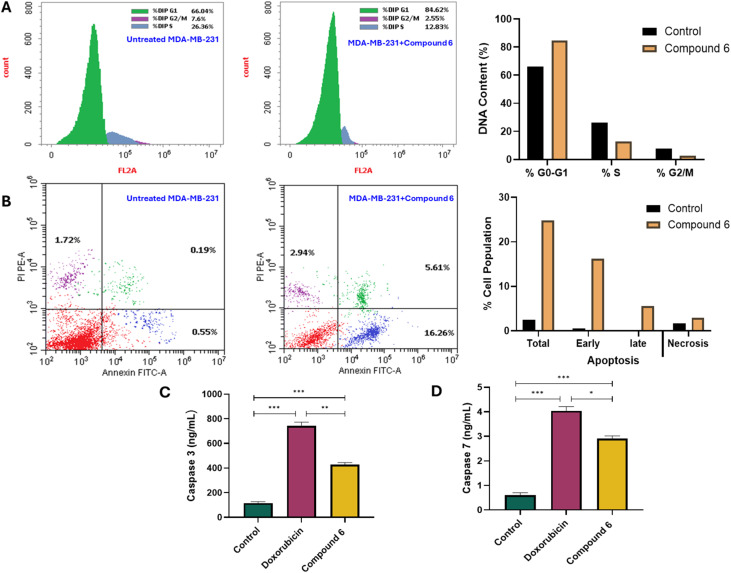
Antiproliferative mechanism of compound 6 in MDA-MB-231 cells assessed by flow cytometry and ELISA analyses. (A) Analysis and distribution of DNA content of cell cycle phases in MDA-MB-231 cells after treatment with compound 6 (2.25 µM) for 24 h, compared with untreated control cells. (B) Cell population analysis assessed by Annexin V-FITC/PI assay on MDA-MB-231 cells after treatment with compound 6 (2.25 µM) for 24 h, compared with untreated control cells. (C and D) Impact of compound 6 (2.25 µM) on triggering the relative expression levels of caspase 3 (C) and caspase 7 (D) in MDA-MB-231 cells, compared with doxorubicin drug (2.25 µM) and untreated control cells.

Based on the screening results of the synthesized compounds, compound 6 exhibited the most potent antiproliferative activity against the examined cancer cell lines. Accordingly, we focused on elucidating its mode of action in MDA-MB-231 cells by assessing its effects on cell cycle arrest and apoptosis induction using flow cytometry and ELISA analyses. To investigate the effect of compound 6 on cell cycle progression, MDA-MB-231 cells were treated with compound 6 at its IC_50_ concentration (2.25 µM) for 24 h, and the DNA content distribution was analyzed by flow cytometry. As shown in Fig. 3, treatment with compound 6 induced marked changes in cell cycle phase distribution compared to untreated control cells. Compound 6-treated cells exhibited a substantial accumulation in the G_0_/G_1_ phase (84.62%) with a concomitant decrease in the S phase (12.83%) and G_2_/M phase (2.55%). In contrast, control cells showed 66.04% in G_0_/G_1_, 26.36% in S phase, and 7.60% in G_2_/M phase. Aligned with our findings, several studies reported the ability of 1,3-thiazolodin-4-one analogues to induce cell cycle arrest at G_0_/G_1_ phase in different cancer cell lines.^41,42^ These results indicate that compound 6 exerts a strong G_1_ phase arrest in MDA-MB-231 cells, effectively halting progression into the DNA synthesis (S) and mitotic (G_2_/M) phases. The pronounced G_1_ accumulation suggests that compound 6 may interfere with cell cycle regulatory proteins controlling the G_1_/S transition, thereby suppressing DNA replication and subsequent cell proliferation. We next investigated whether the antiproliferative effect of compound 6 on MDA-MB-231 cells was associated with the induction of programmed cell death, using Annexin V-FITC/PI staining followed by flow cytometry. MDA-MB-231 cells were treated with compound 6 at its IC_50_ concentration (2.25 µM) for 24 h, and the distribution of viable, apoptotic, and necrotic cells was quantified. As shown in Fig. 3, compound 6 treatment resulted in a markedly higher total apoptosis/necrosis rate (24.81% *vs.* 2.46% in control), comprising 16.26% *vs.* 0.55% early apoptosis, 5.61% *vs.* 0.19% late apoptosis, and 2.94% *vs.* 1.72% necrosis. These results clearly indicate that compound 6 induces substantial apoptotic cell death in MDA-MB-231 cells, with early apoptosis being the predominant mode. The relatively low necrotic fraction suggests that the compound primarily activates regulated cell death pathways rather than causing nonspecific membrane damage. The observed increase in both early and late apoptotic populations aligns with its G_1_ cell cycle arrest effect, as blockade of cell cycle progression often precedes activation of apoptotic cascades. Several reports showed that 1,3-thiazolodin-4-one analogues possess the ability to induce apoptosis in various cancer cells.^43–45^

To further elucidate the mechanism underlying the pro-apoptotic effect of compound 6, we examined its impact on the activation of executioner caspases 3 and 7 in MDA-MB-231 cells. As shown in Fig. 3, treatment with compound 6 at its IC_50_ concentration (2.25 µM) for 24 h significantly increased caspase expression compared with untreated controls. Caspase-3 levels were elevated to 3.69-fold (428.56 ± 16.6 pg mL^−1^*vs.* 116.13 ± 4.51 pg mL^−1^, *p* < 0.001), while caspase-7 levels increased 4.83-fold (2.916 ± 0.11 ng mL^−1^*vs.* 0.604 ± 0.03 ng mL^−1^, *p* < 0.001). For comparison, doxorubicin (positive control) was applied, which exhibited stronger caspase activation, with caspase-3 increasing to 6.41-fold (744.03 ± 28.9 pg mL^−1^) and caspase-7 rising 6.70-fold (4.043 ± 0.17 ng mL^−1^), both *p* < 0.0001 relative to control. These findings demonstrate that compound 6 induces apoptosis in MDA-MB-231 cells at least in part through activation of caspase-3 and caspase-7, consistent with its ability to increase early and late apoptotic populations in Annexin V-FITC/PI assays. This caspase-mediated apoptosis complements the observed G_1_ cell cycle arrest, reinforcing the conclusion that compound 6 suppresses tumor cell proliferation *via* a multifaceted cytostatic and cytotoxic mechanism. Taken together, the mechanistic studies revealed that compound 6 exerts its antiproliferative effect on MDA-MB-231 cells through a combination of G_0_/G_1_ cell cycle arrest, induction of early and late apoptosis, and activation of executioner caspases 3 and 7, highlighting its potential as a multitargeted anticancer agent.

#### 2.2.3. Assessment of hormone-related enzyme inhibition

**Fig. 4:**
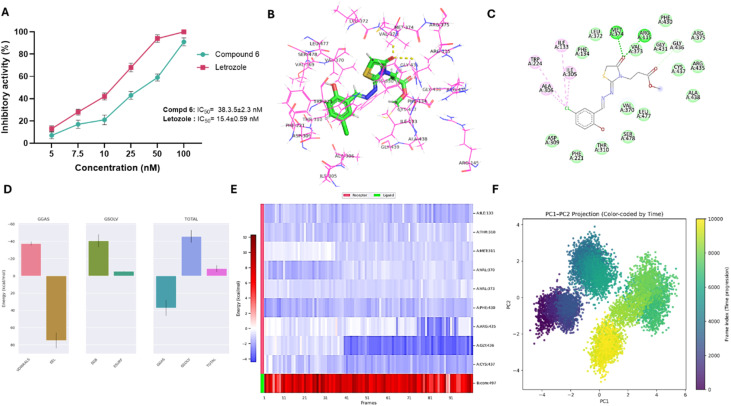
Inhibitory activity and binding affinity of compound 6 toward aromatase (CYP19A1) protein. (A) Representative dose-dependent graph showing the antagonist activity of compound 6 and letrozole toward aromatase enzyme activity. Data expressed as mean ± SE from three independent experiments (*n* = 3). (B and C) 3D and 2D interaction diagrams of compound 6 docked with aromatase protein. (D and E) Structural dynamics of aromatase protein calculated during the 100 ns of MD trajectories showing binding free energy estimated for aromatase with the compound 6 by MM/PBSA (D), and heat map for residue-wise energy decomposition in 100 selected frames (E). (F) Principal component analysis and free energy landscape of the aromatase–compound 6 complex illustrating the two-dimensional PC1–PC2 projection color-coded by simulation time (purple to yellow).

To gain further insights into the antiproliferative mechanism of compound 6, the potential of compound 6 to interfere with estrogen biosynthesis pathways was evaluated by assessing its inhibitory activities against two key enzymes: aromatase (CYP19A1) and steroid sulfatase (STS). Both enzymes play critical roles in maintaining estrogen levels in hormone-dependent breast cancers. Aromatase catalyzes the conversion of androgens into estrogens, while STS hydrolyzes sulfated steroid precursors into active estrogens. Inhibiting these pathways can therefore deprive estrogen receptor–positive (ER^+^) tumor cells of a critical growth stimulus.^46^ Toward aromatase protein, we examined the ability of compound 6 to inhibit aromatase (CYP19A1) activity using a fluorometric assay. Purified aromatase enzyme was incubated with various concentrations of compound 6 in the presence of a fluorogenic aromatase substrate, and fluorescence was monitored at Ex/Em = 488/527 nm. As shown in Fig. 4, compound 6 inhibited aromatase activity in a dose-dependent manner, with an IC_50_ value of 38.3 ± 2.3 nM. For comparison, the clinically used aromatase inhibitor letrozole displayed an IC_50_ of 15.4 ± 0.59 nM under the same conditions. The potent inhibition of aromatase by compound 6 suggests that, in addition to its cytostatic and pro-apoptotic activities, it may exert part of its antiproliferative effect through suppression of estrogen biosynthesis. Although less potent than letrozole, compound 6's nanomolar inhibitory activity underscores its potential dual functionality as both a direct cytotoxic agent and a modulator of hormone-dependent cancer pathways. To further elucidate the molecular basis of aromatase inhibition by compound 6, molecular docking and 100 ns molecular dynamics (MD) simulations were performed using the aromatase crystal structure (PDB ID: 3eqm). Redocking the native co-crystallized ligand into the active site produced a binding affinity of −8.01 kcal mol^−1^ and an RMSD of 0.20 Å, accurately reproducing the experimental pose and forming two hydrogen bonds with Arg115 and Met374, residues located near the heme prosthetic group and critical for catalysis (Fig. S9A, B and Table S2). Docking of compound 6 yielded a binding affinity of −7.82 kcal mol^−1^, closely matching that of the native ligand, and reproduced the same hydrogen bond interactions with Arg115 and Met374 (Fig. 1A and B). In the subsequent MD simulations, the protein backbone RMSD (Fig. S9C) stabilized after ∼30 ns, fluctuating around 0.25 ± 0.1 nm, while the ligand RMSD (Fig. S9D) stabilized within 10 ns and remained below 0.1 nm, confirming a stable binding pose. The radius of gyration (Fig. S9E) and solvent-accessible surface area (Fig. S9F) also remained stable, indicating preserved protein compactness and solvent exposure during the simulation. RMSF analysis (Fig. S9G and H) showed minimal fluctuations (<0.2 nm) for key binding site residues, with Arg115 being the most rigid, alongside Ile133, Cys437, Gly436, Pro429, and Phe430, supporting a stable interaction network. Hydrogen bond monitoring revealed the persistent formation of 1–3 stable hydrogen bonds, with occasional transient fourth bonds, consistent with the docking pose (Fig. 4). MM/PBSA binding free energy analysis (Fig. 4) yielded a Δ*G*_binding of −8.66 ± 3.80 kcal mol^−1^, with van der Waals interactions (−37.59 kcal mol^−1^) providing the main stabilizing force, counterbalancing repulsive electrostatics (+74.77 kcal mol^−1^) through favorable polar (−40.65 kcal mol^−1^) and nonpolar (−5.19 kcal mol^−1^) solvation energies. Per-residue energy decomposition (Fig. 4) identified Arg115 as the dominant contributor *via* hydrogen bonding and electrostatic stabilization, while Ile133 and Ile132 contributed hydrophobic contacts, Cys437 and Gly436 provided polar stabilization, and Pro429 and Phe430 contributed π–sulfur and π–π stacking interactions. Next, PCA was applied to assess the dominant collective motions of aromatase in complex with compound 6 and to complement the docking and MD stability analyses. The eigenvalue spectrum showed that PC1 and PC2 contributed most to the total structural variance and were therefore selected for further evaluation (Fig. S9J). The time evolution of both components indicated multiple phases of coordinated structural adjustment. PC1 displayed three major directional shifts across the trajectory, while PC2 exhibited a pronounced increase between ∼30–50 ns followed by a steady decline, suggesting that the complex sampled several distinct modes of motion rather than simple random fluctuations (Fig. S9K). The PC1–PC2 projection revealed that the aromatase–ligand system explored five low-energy basins throughout the simulation, consistent with transitions between multiple metastable conformational states. Early in the trajectory, these basins were more widely separated, indicating broader conformational exploration. In contrast, the final three basins appeared in close proximity, forming a compact region on the free-energy landscape. This clustering suggests that the system progressively settled into a more restricted and energetically favorable conformational space during the latter half of the run (Fig. 4). These results indicate that aromatase undergoes meaningful large-scale rearrangements upon ligand binding but ultimately converges toward a stable dynamic state, supporting the RMSD and MM/PBSA evidence for a well-maintained and favorable binding mode. Collectively, these computational findings confirm that compound 6 is stably accommodated within the aromatase active site, maintains key interactions with catalytically important residues, and displays favorable binding energetics, providing a strong structural rationale for its potent experimental aromatase inhibition.

**Fig. 5:**
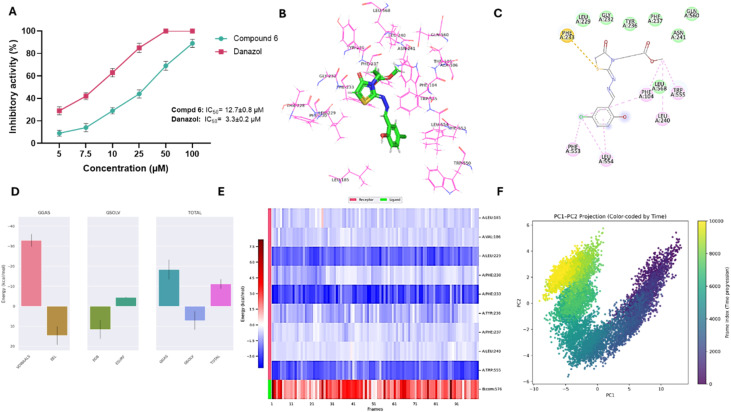
Inhibitory activity and binding affinity of compound 6 toward steroid sulfatase protein. (A) Representative dose-dependent graph showing the antagonist activity of compound 6 and danazole toward steroid sulfatase activity. Data expressed as mean ± SE from three independent experiments (*n* = 3). (B and C) 3D and 2D interaction diagrams of compound 6 docked with steroid sulfatase protein. (D and E) Structural dynamics of steroid sulfatase protein calculated during the 100 ns of MD trajectories showing binding free energy estimated for steroid sulfatase with the compound 6 by MM/PBSA (D), and heat map for residue-wise energy decomposition in 100 selected frames (E). (F) Principal component analysis and free energy landscape of STS–compound 6 complex illustrating the two-dimensional PC1–PC2 projection color-coded by simulation time (purple to yellow).

**Fig. 6:**
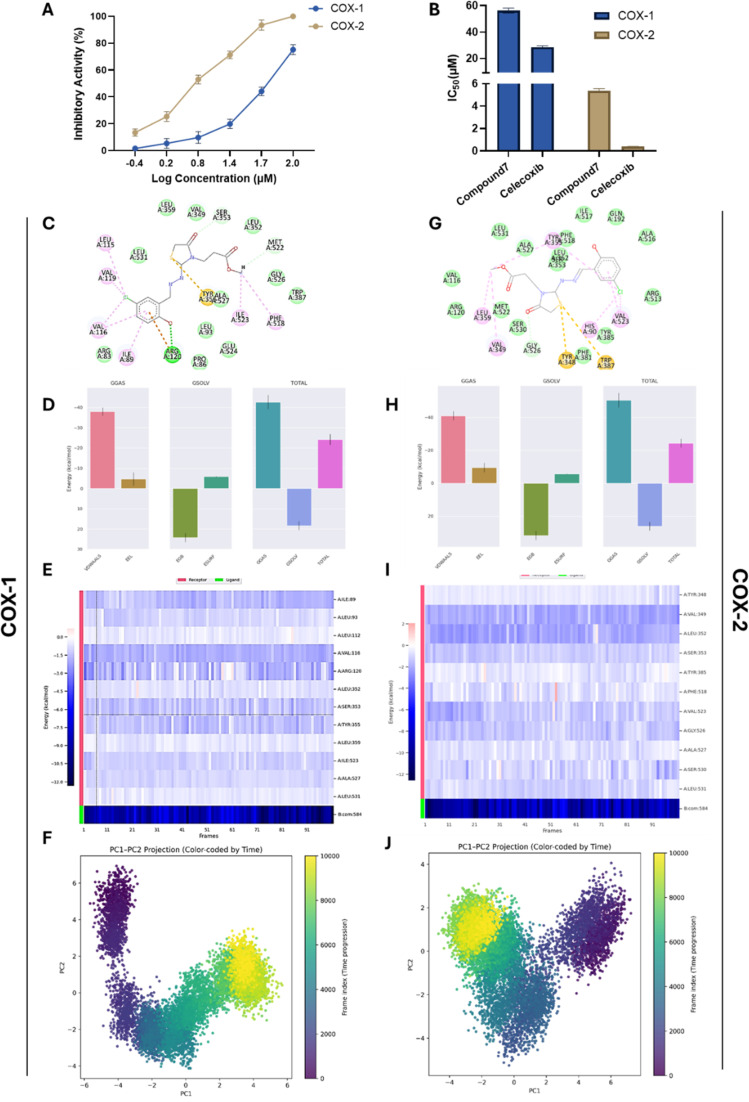
Inhibitory activity and binding affinity of compound 6 toward cyclooxygenase 1 & 2 activity. (A and B) Representative dose-dependent and IC_50_ values graphs showing the antagonist activity of compound 6 and celecoxib toward cyclooxygenase 1 & 2 activity. Data expressed as mean ± SE from three independent experiments (*n* = 3). (C and G) 2D interaction diagrams of compound 6 docked with COX-1 (C) and COX-2 (G) proteins. (D–I) Structural dynamics of COX-1 & 2 proteins calculated during the 100 ns of MD trajectories showing binding free energy estimated for COX-1 (D) and COX-2 (H) with the compound 6 by MM/PBSA, and heat map for residue-wise energy decomposition in 100 selected frames for COX-1 (E) and COX-2 (I). (F–J) Principal component analysis and free energy landscape of COX-1 (F) and COX-2 (J) complexes illustrating the two-dimensional PC1–PC2 projection color-coded by simulation time (purple to yellow).

Toward steroid sulfatase activity, the potency of compound 6 to inhibit STS activity was assessed in comparison to the well-known inhibitor danazol, utilizing the chemiluminescent sandwich assay. As depicted in Fig. 5, compound 6 demonstrated a substantial inhibitory activity in a dose-dependent manner toward STS activity and displayed an IC_50_ of 12.7 ± 0.76 µM, compared with danazol with an IC_50_ of 3.3 ± 0.2 µM. While this potency is moderate relative to danazol, the result is of interest because STS plays a central role in estrogen biosynthesis, particularly in ER^+^ breast cancers, by converting inactive estrone sulfate into active estrogens. While the antiproliferative studies in this work were conducted in ER^−^ MDA-MB-231 cells, where STS inhibition would not be expected to contribute directly to the observed cytotoxicity, the ability of compound 6 to inhibit STS is noteworthy. It highlights the potential for this scaffold to exert therapeutic effects in ER^+^ breast cancer models, where STS-mediated estrogen production plays a key role in tumor growth. Following the observed *in vitro* STS inhibition by compound 6, molecular docking and 100 ns MD simulations were performed to gain deeper insight into its binding mode and stability within the STS active site. Unlike aromatase, the available STS crystal structure lacked a suitable co-crystallized ligand for RMSD validation; therefore, no redocking comparison was conducted. Docking of compound 6 produced a binding affinity of −6.81 kcal mol^−1^ and revealed a notable π–sulfur interaction with Phe233, a key hydrophobic residue lining the lipophilic binding pocket (Fig. 5 and Table S2), suggesting a binding mode that could interfere with STS enzymatic activity. In MD simulations, the protein backbone RMSD (Fig. S10A) equilibrated within 20 ns and remained stable between 0.3–0.4 nm, while the ligand RMSD (Fig. S10B) stabilized from the beginning of the trajectory with minimal fluctuations (0.1–0.2 nm), indicating a persistent binding pose. The radius of gyration (Fig. S10C) and SASA (Fig. 6D) also remained stable, confirming preserved protein compactness and solvent exposure. RMSF analysis (Fig. S10D) showed minimal fluctuations (<0.2 nm) for key binding residues, including Phe233, Trp555, and Phe230, indicating a rigid and stable binding site. Hydrogen bond analysis (Fig. S10E and F) revealed the persistent formation of one stable hydrogen bond throughout the simulation, supporting the docking pose. MM/PBSA binding free energy calculations yielded a favorable Δ*G*_binding of −11.14 ± 2.41 kcal mol^−1^, dominated by van der Waals interactions (−32.90 kcal mol^−1^) with limited electrostatic contributions, consistent with the nonpolar nature of the STS binding pocket (Fig. 5). Per-residue energy decomposition identified Phe233 as the primary stabilizing residue (−2.86 kcal mol^−1^) through van der Waals and π–sulfur interactions, with additional contributions from Leu229 (−2.41 kcal mol^−1^), Trp555 (−2.50 kcal mol^−1^), Ile226 (−0.31 kcal mol^−1^), and aromatic residues such as Phe230, Phe237, and Tyr236, which engaged in π–π and π–alkyl contacts. Minor contributions from Gly436 and Asn241 near the polar rim provided local electrostatic stabilization. These analyses indicate that the STS–compound 6 complex is stabilized primarily by hydrophobic packing and aromatic interactions deep within the lipophilic pocket, in agreement with the experimental inhibitory activity and supporting its potential as a scaffold for STS inhibitor development (Fig. 5). PCA was also performed to characterize the dominant collective motions of STS upon binding compound 6 and to integrate these observations with the docking and MD analyses. The eigenvalue distribution showed that PC1 and PC2 accounted for most of the total variance, indicating that they represent the principal dynamical modes of the STS–ligand complex (Fig. S10H). The time evolution of the components revealed substantial early adjustments: PC1 decreased steadily from high positive values during the first ∼50 ns before stabilizing, while PC2 showed a similar downward trend until ∼40 ns and then gradually shifted toward positive values, plateauing during the final portion of the trajectory. These patterns indicate a pronounced early reorganization followed by convergence into a more equilibrated structural regime (Fig. S10I). The PC1–PC2 projection confirmed this behavior. The FEL displayed several low-energy basins, reflecting transitions through multiple metastable conformational states during the simulation. Early basins were more widely dispersed, consistent with broad initial conformational sampling, whereas later basins clustered tightly within a restricted region, forming a compact low-energy zone. This contraction of the landscape indicates that STS progressively stabilized into a well-defined and energetically favorable conformation as the simulation advanced (Fig. 5). These findings align with the stable RMSD profile and favorable MM/PBSA binding energy, demonstrating that compound 6 induces early global adjustments but ultimately promotes a stable binding mode within the STS active site. Collectively, these findings indicate that compound 6 has the capacity to interfere with two major estrogen-producing pathways, aromatase-mediated androgen conversion and STS-mediated hydrolysis of steroid sulfates, thereby expanding its potential utility beyond direct cytotoxicity to include hormone-dependent breast cancer.

#### 2.2.4. Assessment of cyclooxygenase inhibition

To explore the influence of compound 6 on key inflammatory targets relevant to cancer progression, we aimed to assess its ability to target the cyclooxygenase isoforms utilizing a fluorometric assay. Our results revealed dose-dependent inhibition of both enzymes, with IC_50_ values of 56.27 ± 1.89 µM for COX-1 and 5.38 ± 0.18 µM for COX-2, corresponding to a selectivity index (SI = COX-1 IC_50_/COX-2 IC_50_) of 10.44. This indicates a marked preference for COX-2 over COX-1 inhibition (Fig. 6). For comparison, the reference selective COX-2 inhibitor celecoxib exhibited IC_50_ values of 28.51 ± 0.96 µM for COX-1 and 0.391 ± 0.01 µM for COX-2, with a much higher SI of 72.94 (Fig. S8). Although celecoxib was considerably more potent and selective, the data suggest that compound 6 possesses appreciable COX-2 inhibitory activity while maintaining reduced COX-1 inhibition. Various reports revealed that 1,3-thiazolodin-4-one-based compounds display substantial selectivity and inhibitory activity toward COX-2 protein.^26,47^ Given the established role of COX-2 in tumor-promoting inflammation, angiogenesis, and metastasis, the observed COX-2 selectivity of compound 6 may contribute to its antiproliferative activity and provide an additional therapeutic mechanism alongside its direct cytotoxic and hormone-modulating effects. To investigate the molecular basis of COX inhibition and COX-2 selectivity, compound 6 was subjected to molecular docking and 100 ns MD simulations against both COX-1 and COX-2. For COX-1, docking produced a binding affinity of −6.57 kcal mol^−1^, compared to −7.48 kcal mol^−1^ for the native ligand (RMSD 0.15 Å), reproducing key NSAID interactions: a hydrogen bond and π–cation contact with Arg120, and a π–sulfur interaction with Tyr355 (Fig. 6 and Table S2). MD simulations confirmed complex stability, with backbone RMSD ∼0.3 nm (Fig. S11C), ligand RMSD 0.15–0.25 nm (Fig. S11D), stable *R*_g_ (∼2.45 nm) and SASA (295–305 nm^2^) (Fig. 11E and F), and minimal RMSF (<0.2 nm) for binding site residues (Fig. S11G). A persistent hydrogen bond with Arg120 was maintained throughout the simulation (Fig. S11H). MM/PBSA analysis yielded a Δ*G* binding of −24.23 ± 2.63 kcal mol^−1^, driven mainly by van der Waals interactions (−37.93 kcal mol^−1^) with modest electrostatics (−4.71 kcal mol^−1^) and an unfavorable polar solvation penalty (+24.28 kcal mol^−1^). Per-residue identified Arg120 (−1.58 kcal mol^−1^) and Tyr355 (−1.48 kcal mol^−1^) as major contributors, supported by hydrophobic residues Val116, Ile89, and Leu93, consistent with a binding mode dominated by nonpolar packing decomposition (Fig. 6). PCA was further performed to characterize the dominant motions of COX-1 upon binding compound 6. The eigenvalue distribution indicated that PC1 and PC2 accounted for the majority of the structural variance, identifying them as the key collective modes of the COX-1–ligand complex (Fig. S11J). The time evolution of these components showed that PC1 increased steadily during the first ∼80 ns before stabilizing, while PC2 exhibited an opposite trend, decreasing over the initial 20–30 ns and then oscillating around a stable range. These trajectories reflect early large-amplitude motions associated with relaxation from the docked pose, followed by convergence toward a more equilibrated state (Fig. S11K). The PC1–PC2 projection further illustrated this behavior. In the first ∼20 ns, the complex sampled a broader region of conformational space, indicating rapid structural adjustment. As the simulation progressed, the conformational sampling contracted into a compact cluster of closely positioned low-energy basins. The FEL revealed a shallow early basin that evolved into a deeper, well-defined minimum dominating the later stages of the trajectory (Fig. 6). Together, these results reveal that COX-1 undergoes initial structural reorganization upon ligand binding but ultimately settles into a stable and energetically favorable conformation, consistent with the RMSD patterns and favorable MM/PBSA binding energies.

For COX-2, docking gave a binding affinity of −6.98 kcal mol^−1^, compared to −8.30 kcal mol^−1^ for the native ligand (RMSD 0.83 Å), engaging Tyr355 and Trp387 *via* π–sulfur interactions, though not replicating the hydrogen bonding network of the native ligand (Fig. 6 and Table S2). MD simulations indicated high stability, with backbone RMSD ∼0.3 ± 0.1 nm (Fig. S12C), ligand RMSD <0.05 nm (Fig. S12D), stable *R*_g_ and SASA (Fig. S12E and F), and minimal RMSF (<0.2 nm) for key residues (Arg120, Gln192, Tyr355, Trp387) (Fig. S12G). Three persistent hydrogen bonds were maintained, with a transient fourth (Fig. S12H). MM/PBSA analysis showed a Δ*G*_binding of −24.3 ± 2.67 kJ mol^−1^, dominated by van der Waals forces (−41.0 kJ mol^−1^) and moderate electrostatics (−9.47 kJ mol^−1^), offset by a polar solvation penalty (+31.79 kJ mol^−1^). Per-residue decomposition highlighted strong contributions from Arg120 (−251.32 kJ mol^−1^, electrostatics), Trp387 (−58.13 kJ mol^−1^), Gln192 (−32.30 kJ mol^−1^), and Tyr355 (−1.48 kJ mol^−1^), consistent with persistent π-interactions during MD (Fig. 6). PCA was also performed to examine the major collective motions of COX-2 upon binding compound 6. The eigenvalue distribution showed that PC1 and PC2 accounted for most of the total variance, identifying them as the dominant modes of motion (Fig. S12J). The time evolution of these components revealed that PC1 decreased steadily during the first ∼80 ns before stabilizing, while PC2 showed higher variability in the first 30–40 ns and then converged toward oscillations around zero. This pattern indicates early large-scale rearrangements followed by a transition into a more restricted and equilibrated structural regime (Fig. S12K). The PC1–PC2 projection confirmed this behavior: during the initial 20 ns, the complex sampled a broader region of conformational space, corresponding to rapid relaxation from the starting structure. As the trajectory progressed, the sampling contracted into closely grouped low-energy basins. The FEL displayed a shallow early basin followed by the emergence of a deeper, well-defined minimum that dominated the later stages of the simulation (Fig. 6). These findings indicate that COX-2 undergoes initial conformational exploration but ultimately stabilizes into a compact and energetically favorable state, consistent with the RMSD and MM/PBSA results. Collectively, the computational analyses confirm that compound 6 binds stably to both COX isoforms, with COX-1 interactions dominated by Arg120/Tyr355 and hydrophobic residues, while COX-2 binding is stabilized by Arg120, Trp387, Gln192, and Tyr355. The binding profiles align with the experimental IC_50_ data, supporting the observed COX-2 selectivity and providing a structural basis for its inhibitory activity.

#### 2.2.5. Evaluation of DPPH scavenging activity

**Fig. 7:**
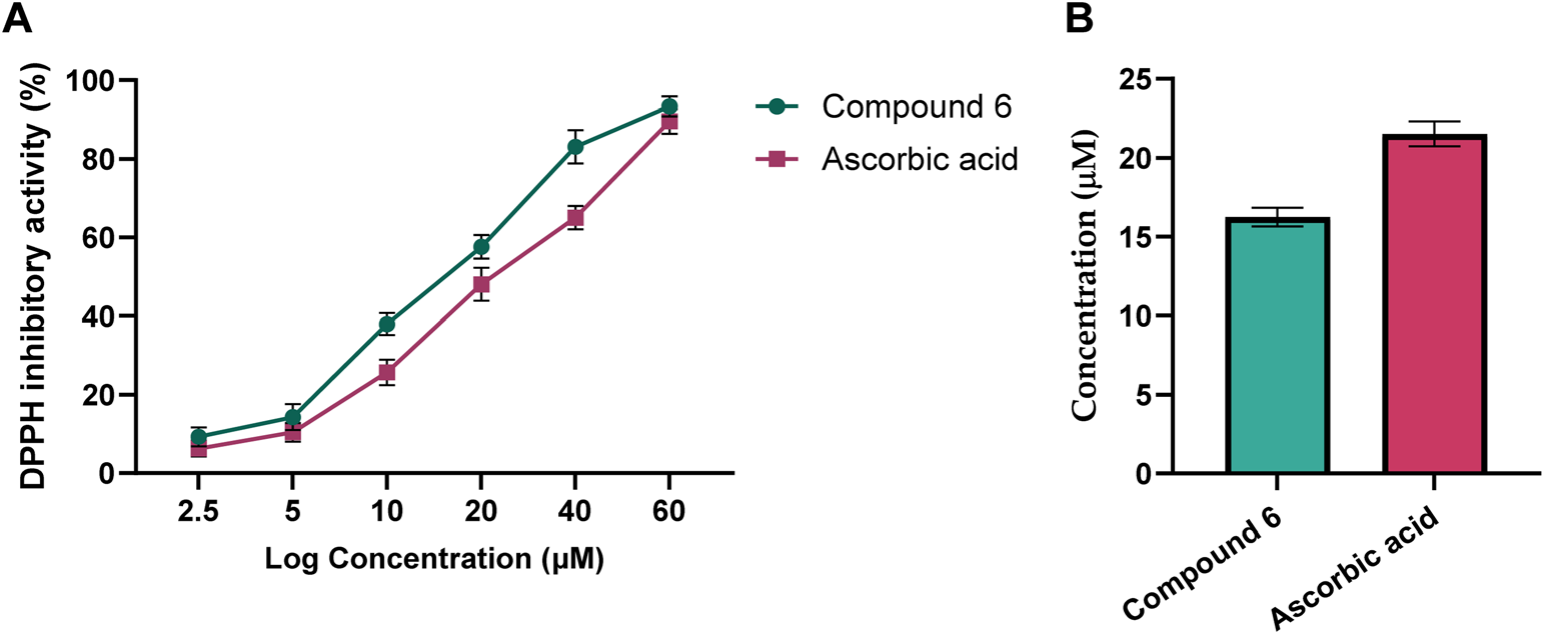
Inhibitory activity of compound 6 toward DPPH activity, as compared to ascorbic acid. Representative dose-dependent (A) and IC_50_ values (B) graphs showing the inhibitory activity of compound 6 and ascorbic acid toward DPPH activity. Data expressed as mean ± SE from three independent experiments (*n* = 3).

We finally aimed to assess the antioxidant potential of compound 6. Toward this, the antioxidant capacity of compound 6 was assessed using the 2,2-diphenyl-1-picrylhydrazyl (DPPH) assay, which measures the ability to neutralize free radicals, a key defense mechanism against oxidative stress.^48^ As shown in Fig. 7, compound 6 exhibited marked, concentration-dependent scavenging activity with an IC_50_ of 16.26 ± 0.6 µM, surpassing the standard antioxidant ascorbic acid (IC_50_ = 21.36 ± 0.8 µM). This higher potency suggests a strong radical-neutralizing capability. The observed antioxidant activity is particularly relevant in the context of our earlier findings, as oxidative stress plays a pivotal role in cancer progression, inflammation, and metastasis. These findings are in agreement with reported studies, which showed that 1,3-thiazolidin-4-one analogs possess considerable antioxidant potential and inhibitory activity toward DPPH radicals.^23,49^ The COX-2 inhibitory effect of compound 6 may synergize with its free radical scavenging ability to reduce tumor-promoting inflammation, while its pro-apoptotic and cell cycle-arresting actions directly impair tumor growth. Collectively, these results position compound 6 as a multifunctional agent with complementary anticancer, anti-inflammatory, and antioxidant activities, supporting its promise for further preclinical evaluation.

### 2.3 *In vivo* validation of anticancer impact in an EAC-induced model

#### 2.3.1. Assessment of acute toxicity and optimal therapeutic dose

**Fig. 8:**
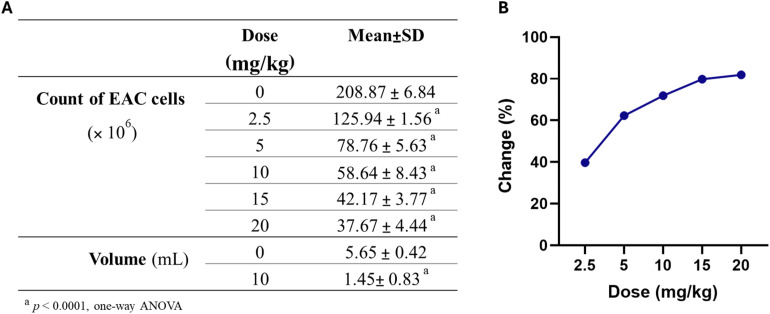
Dose-dependent effect of compound 6 on EAC cell count and ascitic tumor volume. (A) Reduction in total viable EAC cell count and tumor volume following treatment with different doses of compound 6 compared to the untreated control group. (B) Percentage change in EAC cell count at each dose relative to the untreated control group. Data are expressed as mean ± SD (*n* = 6). Statistical significance was determined by one-way ANOVA.

The acute toxicity of compound 6 was evaluated in mice using a modified Meier and Theakston protocol to establish its safety profile and guide subsequent dosing in the *in vivo* tumor model. Animals received intraperitoneal injections of escalating doses of compound 6 (1–200 mg kg^−1^) and were closely monitored for 48 hours for mortality, behavioral changes, and overt signs of toxicity. At doses up to 120 mg kg^−1^, no mortality or abnormal behavioral manifestations were observed, and the animals maintained normal grooming, feeding, and locomotor activity, indicating a good safety margin at these concentrations. In contrast, administration of 200 mg kg^−1^ resulted in complete mortality, establishing this dose as the approximate lethal dose (LD_100_). Accordingly, the LD_0_ of compound 6 was determined to be 120 mg kg^−1^. In light of these observations, doses well below the LD_0_ were selected for subsequent pharmacological evaluation in the EAC-induced tumor model to ensure that the observed effects would reflect genuine therapeutic efficacy rather than nonspecific toxicity. Following the acute toxicity evaluation, the therapeutic efficacy of compound 6 was assessed *in vivo* using the EAC-induced mouse model. Animals were treated intraperitoneally with escalating doses of compound 6 (2.5, 5, 10, 15, and 20 mg kg^−1^) every two days for a period of 10 days, and the impact on viable EAC cell counts and ascitic tumor volume was determined. As summarized in Fig. 8, compound 6 elicited a pronounced and dose-dependent reduction in viable EAC cells compared to the untreated control group (208.87 ± 6.84 × 10^6^ cells). At the lowest tested dose of 2.5 mg kg^−1^, the viable cell count was reduced to 125.94 ± 1.56 × 10^6^, corresponding to a 39.7% decrease. Increasing the dose to 5 mg kg^−1^ and 10 mg kg^−1^ further suppressed cell viability to 78.76 ± 5.63 × 10^6^ (62.3% reduction) and 58.64 ± 8.43 × 10^6^ (71.9% reduction), respectively. At higher doses of 15 and 20 mg kg^−1^, the number of viable cells was further reduced to 42.17 ± 3.77 × 10^6^ (79.8%) and 37.67 ± 4.44 × 10^6^ (81.9%), respectively, indicating a near-maximal effect beyond 15 mg kg^−1^. A parallel evaluation of ascitic tumor volume revealed that treatment with 15 mg kg^−1^ of compound 6 markedly reduced the mean tumor volume to 1.45 ± 0.83 mL, representing a 74.3% reduction relative to the untreated EAC control (5.65 ± 0.42 mL). These findings demonstrate that compound 6 exerts potent antitumor activity *in vivo*, characterized by both reduced viable tumor cell load and suppression of ascitic tumor growth. These data suggest a plateau effect beyond 15 mg kg^−1^, with 10–15 mg kg^−1^ representing the most effective therapeutic window. The *in vivo* efficacy observed here aligns closely with our earlier *in vitro* studies, where compound 6 exhibited strong cytotoxic, pro-apoptotic, and enzyme-inhibitory activity, collectively supporting its potential as a promising anticancer candidate.

#### 2.3.2. Evaluation of hepatorenal function and histopathological alterations

**Fig. 9:**
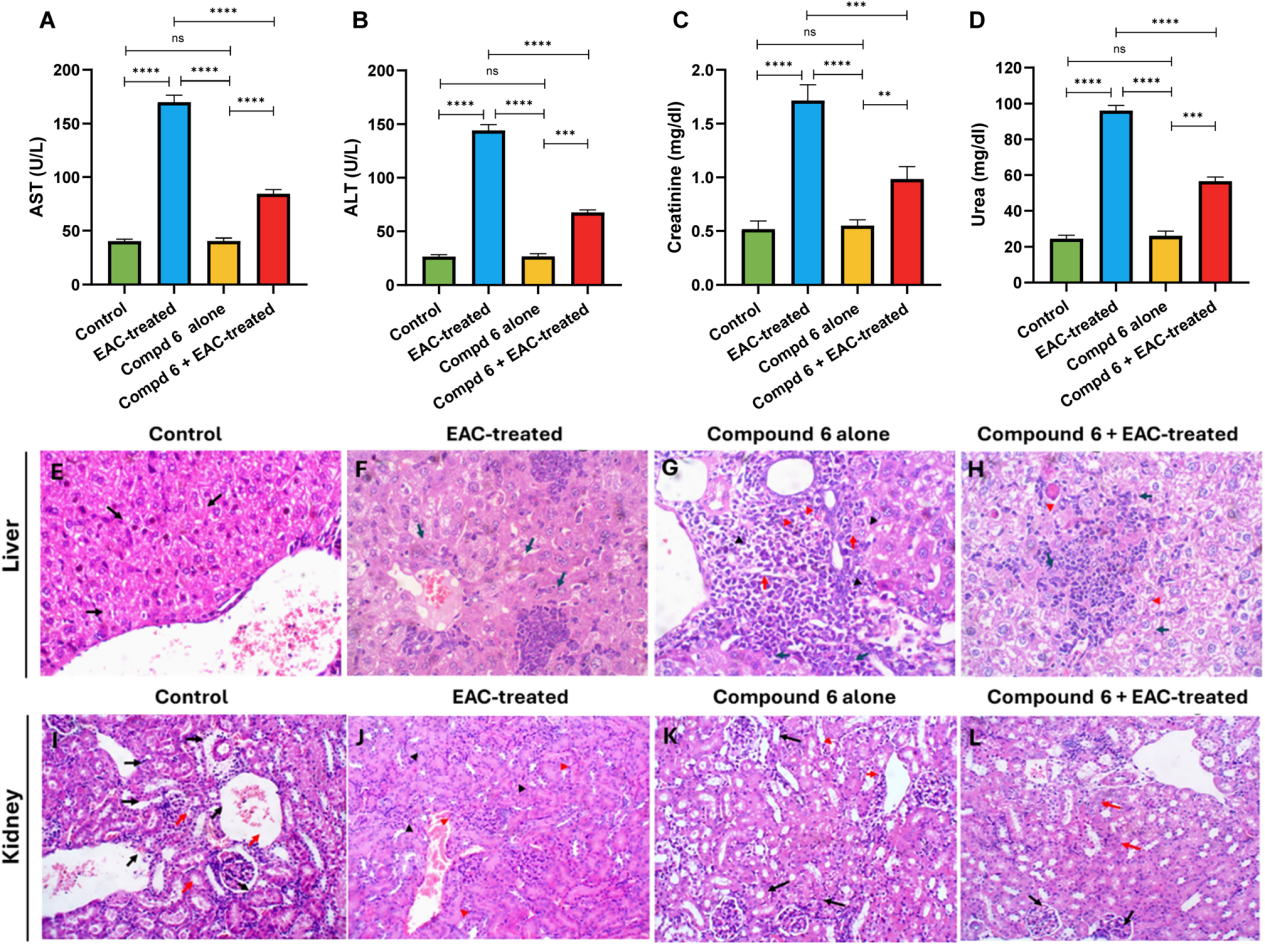
Effect of compound 6 on liver and kidney function in EAC-bearing mice. (A–D) Serum biochemical parameters (ALT, AST, creatinine, and urea) across untreated control, EAC control, EAC + compound 6-treated, and compound 6-only groups. Data are expressed as mean ± SD (*n* = 6), with statistical significance assessed by one-way ANOVA. (E–H) Representative liver histology (H&E, 40×). The untreated control showed normal hepatic architecture with orderly hepatocyte cords (E). The EAC-induced control group exhibited hydropic degeneration, lobular inflammation, and portal inflammatory infiltrates (F). Treatment with compound 6 markedly improved liver histology with preserved hepatocyte structure and minimal inflammation (G), while the drug-only group showed mild hydropic changes and scattered apoptotic hepatocytes (H). (I–L) Representative kidney histology (H&E, 20×). The untreated control displayed normal renal glomeruli and tubules (I). The EAC control showed mesangial proliferation, focal glomerulosclerosis, and acute tubular injury (J). Treatment with compound 6 restored renal integrity with only mild tubular changes (K), whereas the compound 6-only group exhibited minimal focal alterations without significant pathology (L).

To further validate the therapeutic efficacy and systemic safety of compound 6 in EAC-bearing mice, we assessed serum biochemical markers of liver and kidney function alongside histopathological examination of the respective tissues. This dual approach was intended to determine whether compound 6 could not only reduce tumor progression but also protect vital organs from EAC-induced damage, a common limitation of many chemotherapeutic agents. Biochemical analysis revealed that EAC induction caused profound alterations in hepatic and renal function (Fig. 9). Compared with the untreated control group (ALT: 26.50 ± 1.87 U L^−1^, AST: 40.5 ± 1.87 U L^−1^, creatinine: 0.51 ± 0.07 mg dL^−1^, urea: 24.50 ± 1.87 mg dL^−1^), the EAC-control group exhibited a striking elevation of ALT (144.16 ± 5.63 U L^−1^) and AST (170 ± 6.6 U L^−1^), reflecting severe hepatocellular damage. Similarly, serum creatinine (1.71 ± 0.14 mg dL^−1^) and urea (96.16 ± 2.85 mg dL^−1^) were markedly increased, consistent with impaired renal function. Treatment with compound 6 significantly restored these biochemical markers toward normal values (ALT: 67.50 ± 2.42 U L^−1^, AST: 84.5 ± 3.83 U L^−1^, creatinine: 0.98 ± 0.11 mg dL^−1^, urea: 60.50 ± 2.42 mg dL^−1^), clearly demonstrating its protective role in counteracting EAC-induced hepatic and renal dysfunction. Importantly, the compound 6 control group exhibited values comparable to those of the untreated control (ALT: 26.66 ± 2.58 U L^−1^, AST: 40.67 ± 2.73 U L^−1^, creatinine: 0.55 ± 0.05 mg dL^−1^, urea: 25.00 ± 2.60 mg dL^−1^), indicating that compound 6 is safe at the tested dose and does not inherently compromise hepatic or renal physiology (Fig. 9). Histological analysis corroborated these biochemical findings (Fig. 9E–L). In the liver, the untreated control group displayed normal histoarchitecture, with cords of hepatocytes arranged radially around central veins and intact portal tracts (black arrows), showing no signs of inflammation, degeneration, or fibrosis (score 0, H&E, 20×) (Fig. 9E–I). In contrast, the EAC control group exhibited severe pathological changes, including enlargement of the portal tract with dense inflammatory infiltrates (black arrowheads), hepatocytic hydropic degeneration (red arrowheads), and multiple foci of lobular inflammation (blue arrows). The hepatic injury was graded as hydropic degeneration (45%, grade 2), lobular inflammation (2 foci, grade 2), and pronounced portal inflammation (grade 3), resulting in a total histological activity score of 7 (H&E, 40×). These changes confirm that EAC markedly disrupts liver structure through inflammatory and degenerative mechanisms. Treatment with compound 6 markedly improved hepatic histology. Liver sections from the compound 6-treated EAC-induced group revealed no portal tract inflammation, no interface hepatitis, and no hydropic degeneration. Only scattered foci of lobular inflammation and apoptotic hepatocytes were observed (blue arrows), resulting in a much lower overall score (grade 3, stage 0). This restoration of hepatic architecture strongly supports the biochemical evidence that compound 6 mitigates EAC-induced hepatotoxicity, likely through its antioxidant and anti-inflammatory activities. In the compound 6 control group, liver sections showed generally preserved architecture with no portal inflammation or fibrosis; however, limited hydropic degeneration (∼20% of hepatocytes, red arrowheads) and scattered apoptotic cells (blue arrows) were evident, leading to a slightly higher activity grade of 4. These mild alterations suggest that while compound 6 is largely safe, minimal adaptive hepatic responses may occur, though not reflected in serum enzyme elevations. Kidney histology showed a similar pattern (Fig. 9I–L). The untreated control group presented normal renal histology with intact glomeruli (black arrows) and regular tubular architecture (red arrows), scoring 0 (H&E, 20×). The EAC control group, however, displayed significant renal pathology, including mesangial proliferation with focal glomerulosclerosis (black arrowheads) and acute tubular injury in several tubules (red arrowheads). Tubular damage was estimated at 30% (score 2+) and glomerular changes at 25%, indicative of early nephropathy (class II). Remarkably, treatment with compound 6 restored renal integrity, as kidney sections revealed uniformly normal glomeruli with no structural abnormalities, while only ∼10% of tubules displayed mild acute injury (red arrowheads), corresponding to a low score of 1+. These findings confirm the protective role of the compound against EAC-induced nephropathy. In the compound 6 control group, mild pathological alterations were noted, including mesangial proliferation with focal sclerosis and limited tubular injury (red arrowheads), though the overall histological score remained minimal (0), aligning with the absence of biochemical abnormalities. Together, biochemical and histological data provide compelling evidence that compound 6 not only reduces tumor burden but also prevents secondary organ damage in EAC-bearing mice. This dual activity underscores its therapeutic promise, distinguishing it from conventional chemotherapeutics that often exacerbate hepatic and renal toxicity. The observed protection of hepatocytes and renal tubules is likely mediated through a combination of antioxidant activity, reduction of inflammatory signaling, and preservation of tissue integrity, mechanisms that are consistent with its previously demonstrated *in vitro* cytoprotective and antiproliferative effects.

#### 2.3.3. Assessment of oxidative stress and antioxidant defense

**Fig. 10:**
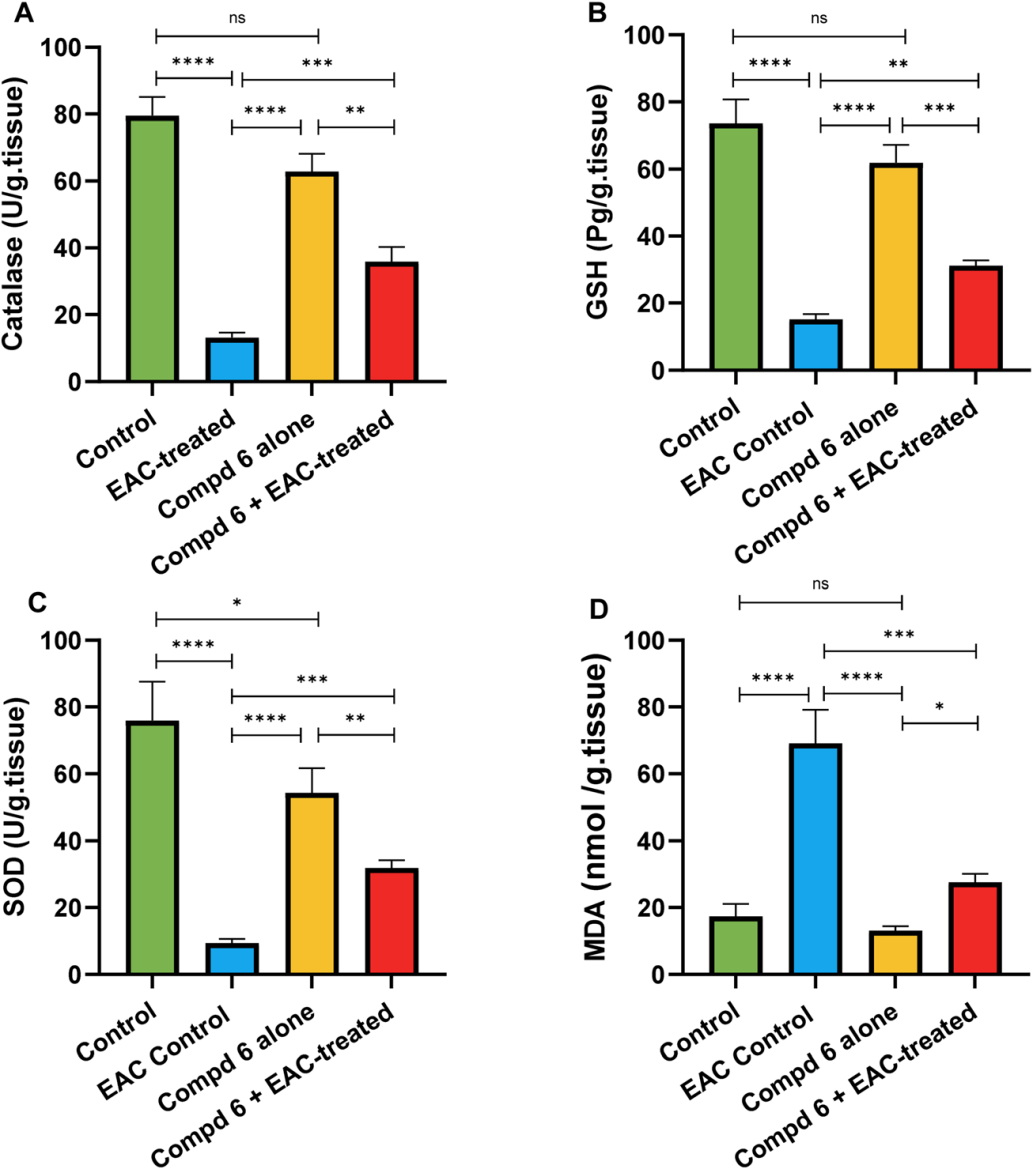
Effect of compound 6 treatment on hepatic oxidative stress and antioxidant defense markers. Levels of catalase (A), glutathione (B), superoxide dismutase (C), and malondialdehyde (D) were measured in liver tissue across the different treatment groups. Data are expressed as mean ± SD (*n* = 6). Statistical analysis was performed using one-way ANOVA.

To further clarify the protective mechanisms underlying the anticancer activity of compound 6, we examined its effect on oxidative stress and antioxidant defense systems in the liver of EAC-bearing mice. Since tumor progression is often associated with elevated oxidative stress and depletion of endogenous antioxidants, assessing these biomarkers provides critical insights into both the therapeutic efficacy and safety of the examined compound. The biochemical analysis revealed that EAC induction markedly impaired the redox balance. In the EAC control group, glutathione (GSH: 14.15 ± 1.54 pg per g tissue), catalase (CAT: 13.08 ± 1.53 U per g tissue), and superoxide dismutase (SOD: 9.39 ± 1.20 U per g tissue) levels were drastically reduced compared with the untreated control group (GSH: 73.61 ± 7.13 pg per g tissue, CAT: 79.50 ± 5.61 U per g tissue, SOD: 75.98 ± 11.61 U per g tissue). Conversely, malondialdehyde (MDA), a lipid peroxidation marker, was significantly elevated (69.10 ± 10.06 *vs.* 17.37 ± 3.78 nmol per g tissue). These findings confirm that EAC burden induces severe oxidative stress and compromises the liver's antioxidant defense capacity (Fig. 10). Treatment with compound 6 led to marked improvement in redox status. Mice in the EAC + compound 6 group displayed significantly higher antioxidant activity (GSH: 31.20 ± 1.57 pg per g tissue, CAT: 35.90 ± 4.42 U per g tissue, SOD: 31.86 ± 2.30 U per g tissue) compared to the EAC control.

Simultaneously, MDA levels dropped to 27.65 ± 2.50 nmol per g tissue, reflecting a substantial attenuation of lipid peroxidation (Fig. 10). These results suggest that compound 6 alleviates oxidative damage by enhancing antioxidant defenses while limiting ROS-mediated injury. Interestingly, although antioxidant enzyme activities were not fully restored to normal values, the partial recovery highlights the drug's protective yet non-toxic nature under tumor-induced oxidative stress conditions. The compound 6 control group further confirmed the safety profile of compound 6, as its oxidative stress markers remained comparable to those of the untreated control (GSH: 61.90 ± 5.29 pg per g tissue, CAT: 62.86 ± 5.32 U per g tissue, SOD: 54.32 ± 7.40 U per g tissue, MDA: 13.16 ± 1.27 nmol per g tissue). This indicates that in the absence of tumor burden, the compound does not perturb hepatic redox homeostasis. Collectively, these findings demonstrate that compound 6 counteracts EAC-induced oxidative stress by restoring antioxidant enzyme activity and suppressing lipid peroxidation. This antioxidant potential not only supports its hepatoprotective properties but may also synergize with its anticancer activity by reducing ROS-driven tumor progression.

#### 2.3.4. Evaluation of inflammatory and angiogenic markers

**Fig. 11:**
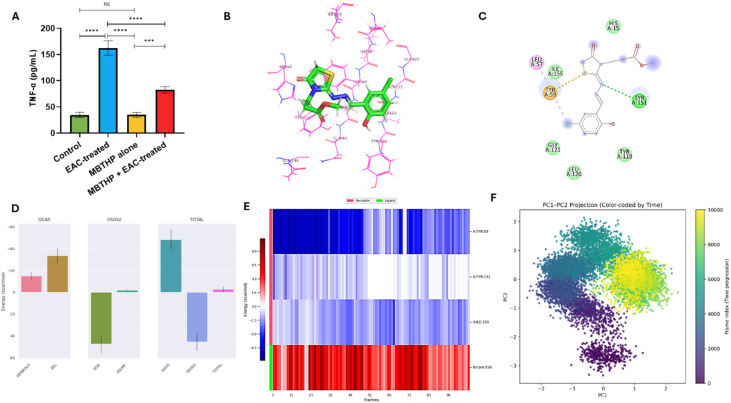
Effect of compound 6 treatment on expression and activity of TNF-α protein. (A) Effect of compound 6 treatment on the expression levels of serum TNF-α in the different treatment groups. Data is presented as mean ± SD from *n* = 6 experiments. The statistical analysis was done by one-way ANOVA. (B and C) 3D and 2D interaction diagrams of compound 6 docked with TNF-α protein. (D–F) Structural dynamics of TNF-α protein calculated during the 100 ns of MD trajectories showing binding free energy estimated for TNF-α with the compound 6 by MM/PBSA (D), and heat map for residue-wise energy decomposition in 100 selected frames (E). (F) Principal component analysis and free energy landscape of TNF-α–compound 6 complex illustrating the two-dimensional PC1–PC2 projection color-coded by simulation time (purple to yellow).

Since inflammation and angiogenesis are fundamental drivers of tumor initiation and progression, we next evaluated the expression of tumor necrosis factor-alpha (TNF-α) and vascular endothelial growth factor receptor II (VEGFR-II) to determine whether compound 6 exerts anticancer activity through modulation of these pathways. This analysis was designed to complement our earlier findings on oxidative stress defense and tissue protection, thereby providing further mechanistic insights into how compound 6 suppresses tumor growth in the EAC model. As shown in Fig. 11, TNF-α levels were markedly elevated in the EAC control group (162.63 ± 13.35 pg mL^−1^) compared to both the untreated control (36.49 ± 4.64 pg mL^−1^) and compound 6 control group (34.43 ± 4.57 pg mL^−1^), reflecting the profound pro-inflammatory response triggered by tumor progression. Treatment with compound 6 (EAC + compound 6) significantly attenuated this rise, reducing TNF-α to 82.27 ± 6.44 pg mL^−1^, which represents almost a 50% decrease compared to the EAC control. Importantly, TNF-α levels remained unaffected in the compound 6-only group, suggesting that compound 6 specifically targets tumor-induced inflammation rather than interfering with normal immune homeostasis. Since TNF-α is a central mediator of inflammation and was significantly elevated in EAC-bearing animals, we further explored the molecular basis of compound 6's interaction with TNF-α using molecular docking and 100 ns MD simulations. This computational study was performed to validate our experimental findings and provide atomistic insights into the compound's anti-inflammatory potential. Docking analysis showed that the co-crystallized ligand bound TNF-α with a binding affinity of −6.25 kcal mol^−1^ and formed stabilizing π–π and hydrogen bond interactions with Tyr59 and Tyr151, validating the docking protocol (RMSD = 0.35 Å) (Fig. S13A, B and Table S2). Compound 6 demonstrated a comparable binding affinity of −5.34 kcal mol^−1^ and preserved key interactions with the same residues, engaging Tyr59 and Tyr151 through π–sulfur and hydrogen bonding contacts. These findings suggest that the compound mimics native binding features within the TNF-α pocket, supporting its potential as a modulator of this cytokine (Fig. 11). MD simulations provided further evidence of stable complex formation. The protein backbone RMSD equilibrated within the first 10 ns and remained stable around 0.30 ± 0.05 nm (Fig. S13C), while ligand RMSD values dropped to <0.025 nm after 40 ns (Fig. S13D), indicating a highly stable binding pose. Structural compactness (*R*_g_) (Fig. S13E) and solvent accessibility (SASA) (Fig. S13F) remained consistent, confirming the absence of major conformational disruptions. RMSF analysis revealed minimal flexibility in key binding residues Tyr59 and Tyr151, underscoring the rigidity of the binding pocket (Fig. S13G and H). Importantly, hydrogen bond analysis showed that compound 6 maintained at least one persistent hydrogen bond throughout the simulation, which stabilized further in the latter half with two stable hydrogen bonds. Binding free energy calculations (MM-PBSA) yielded an average Δ*G*_bind of −2.87 kcal mol^−1^, reflecting a modest but favorable interaction. The binding was primarily driven by van der Waals (−14.87 kcal mol^−1^) and electrostatic (−33.48 kcal mol^−1^) contributions, which were partially offset by polar solvation penalties. Per-residue energy decomposition highlighted Tyr59 and Tyr151 as key contributors to stabilization, consistent with docking and MD results (Fig. 11).

Further, PCA was performed to assess the major collective motions of TNF-α upon binding compound 6. The eigenvalue profile showed that PC1 and PC2 captured most of the overall variance, identifying them as the dominant dynamical modes of the complex (Fig. S12J). The time evolution of these components indicated clear early rearrangements: PC1 fluctuated near 0, shifted toward negative values within the first ∼20 ns, and then increased toward positive values before stabilizing with small oscillations during the final part of the trajectory. PC2 showed a complementary behavior, briefly stabilizing near +1 and then decreasing toward 0, where it maintained restrained fluctuations for the remainder of the simulation (Fig. S12K). The PC1–PC2 projection confirmed these trends. During the initial 10–20 ns, the complex sampled a wider conformational region, reflecting rapid relaxation from the starting pose. As the simulation progressed, the distribution contracted into a compact cluster, indicating reduced structural heterogeneity and increasing dynamical stability. The corresponding free-energy landscape displayed an early, shallow basin followed by the emergence of a deeper, well-defined minimum that dominated the later trajectory. This transition toward a single low-energy basin shows that TNF-α undergoes initial conformational exploration but settles into a stable and energetically favorable state upon ligand binding (Fig. 11). These results align with stable RMSD behavior and favorable MM/PBSA binding energy observed for this complex. Overall, these computational findings confirm that compound 6 can stably associate with TNF-α, engaging critical aromatic residues that play a role in cytokine signaling. The moderate binding affinity and stable interaction profile suggest that the observed experimental reduction in TNF-α levels may be partially attributed to direct modulation of its molecular activity.

**Fig. 12:**
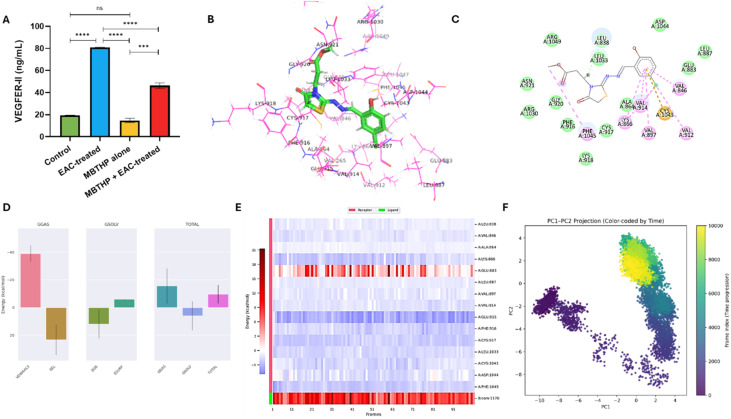
Effect of compound 6 treatment on expression and activity of VEGFER-II protein. (A) Effect of compound 6 treatment on the expression levels of serum VEGFER-II in the different treatment groups. Data is presented as mean ± SD from *n* = 6 experiments. The statistical analysis was done by one-way ANOVA. (B and C) 3D and 2D interaction diagrams of compound 6 docked with VEGFER-II protein. (D–F) Structural dynamics of VEGFER-II protein calculated during the 100 ns of MD trajectories showing binding free energy estimated for VEGFER-II with compound 6 by MM/PBSA (D), and heat map for residue-wise energy decomposition in 100 selected frames (E). (F) Principal component analysis and free energy landscape of VEGFER-II–compound 6 complex illustrating the two-dimensional PC1–PC2 projection color-coded by simulation time (purple to yellow).

Similarly, VEGFR-II levels were dramatically upregulated in the EAC control group (80.76 ± 0.33 ng mL^−1^) compared to the untreated control (19.33 ± 0.27 ng mL^−1^) and compound 6 control group (14.76 ± 1.83 ng mL^−1^), consistent with the angiogenic drive required for ascitic tumor expansion. Treatment with compound 6 substantially reduced VEGFR-II expression to 46.59 ± 2.71 ng mL^−1^, demonstrating a pronounced anti-angiogenic effect (Fig. 12). Together, these results suggest that compound 6 effectively counteracts both tumor-associated inflammation and angiogenesis, two hallmarks of cancer progression. By lowering TNF-α, the compound may reduce pro-inflammatory signaling that fosters tumor cell proliferation and survival. Simultaneously, suppression of VEGFR-II suggests inhibition of angiogenic signaling pathways, thereby limiting the vascular support essential for tumor expansion. These findings are consistent with our earlier biochemical and histological data, where treatment with compound 6 restored liver and kidney function, improved tissue architecture, and enhanced antioxidant defenses. Collectively, this dual anti-inflammatory and anti-angiogenic action underscores the therapeutic promise of compound 6 in targeting multiple cancer-promoting pathways. Given that VEGFR2 plays a pivotal role in angiogenesis and was markedly elevated in EAC-bearing mice, we conducted molecular docking and 100-ns MD simulations to explore whether compound 6 could directly modulate VEGFR2 at the molecular level. Docking analysis showed that compound 6 bound VEGFR2 with a high affinity (−10.20 kcal mol^−1^), stronger than the co-crystallized ligand (−7.87 kcal mol^−1^; RMSD = 0.48 Å) (Fig. S14A, B and Table S2). Compound 6 established multiple stabilizing interactions within the ATP-binding pocket, including hydrogen bonds with Glu883 and Cys917, π–sulfur contacts with Cys1043 and Phe916, a π–anion interaction with Asp1044, and π–π stacking with Phe916 (Fig. 12). In contrast, the co-crystallized ligand formed only a single π–sulfur contact with Cys1043, underscoring the superior interaction profile of compound 6 and its potential as a competitive VEGFR2 inhibitor (Fig. S14A and B). MD simulations further confirmed the stability of this interaction. The protein backbone RMSD equilibrated after ∼25 ns and remained steady between 0.45–0.55 nm (Fig. S14C), while ligand RMSD values stabilized within <0.1 nm (Fig. S14D), indicating a highly anchored binding pose. Structural compactness (*R*_g_) (Fig. S14E) and solvent exposure (SASA) (Fig. S14F) remained stable, demonstrating that ligand binding did not induce global structural instability. RMSF analysis revealed low flexibility (<0.2 nm) in critical binding-site residues (Val846, Val912, Val914, Cys1043), highlighting the rigidity of the interaction pocket (Fig. S14G and H). A persistent hydrogen bond was maintained throughout most of the trajectory, confirming a robust and durable binding mode. Binding free energy calculations (MM-PBSA) yielded an average Δ*G*_bind of −9.57 kcal mol^−1^, consistent with strong and favorable binding. The interaction was mainly driven by van der Waals contacts (−39.01 kcal mol^−1^) and reinforced by non-polar solvation, partially offset by electrostatic penalties. Residue-level decomposition identified Glu883 and Asp1044 as major contributors through hydrogen bonding and π-anion interactions, while Cys917, Cys1043, and Phe916 provided π–sulfur and π–π stabilization. Hydrophobic residues such as Val846, Val912, and Val914 further enhanced ligand anchoring *via* van der Waals forces (Fig. 12).

Finally, PCA was used to examine the large-scale motions of VEGFR-2 upon binding compound 6. The eigenvalue distribution showed that PC1 and PC2 accounted for most of the total variance, identifying them as the dominant modes of motion (Fig. S13J). The time evolution of these components revealed clear early adjustments: PC1 increased from approximately −10 to +3 during the first part of the simulation before stabilizing, while PC2 rose toward +3 after the initial ∼10 ns and then oscillated within a narrow range. This pattern indicates substantial early rearrangements followed by convergence into a more equilibrated regime (Fig. S13K). The PC1–PC2 projection confirmed this behavior. During the first 10–20 ns, the complex sampled a broader region of conformational space, reflecting rapid relaxation from the initial pose. As the simulation progressed, the sampling contracted into closely grouped basins. The corresponding FEL showed an early, shallow minimum followed by a deeper, well-defined low-energy basin that dominated the later part of the trajectory (Fig. 12). Together, these findings demonstrate that VEGFR-2 undergoes initial large-scale motions but ultimately stabilizes into a compact and energetically favorable conformation, consistent with RMSD stability and MM/PBSA binding results. Collectively, these computational findings corroborate our experimental data, demonstrating that compound 6 reduces VEGFR2 levels *in vivo* and directly engages critical residues within the receptor's ATP-binding pocket. This dual evidence highlights its potential role as an angiogenesis modulator in EAC-induced.

### 2.4 *In silico* toxicity and off-target assessment

The *in silico* toxicity profile of compound 6 was evaluated using pkCSM and ProTox-III to complement the experimental selectivity index and *in vivo* safety findings. pkCSM predicted compound 6 to be non-mutagenic (Ames = no), non-sensitizing to skin, and free of hERG I/II inhibition, indicating a low likelihood of genotoxic or cardiotoxic liabilities. The predicted human maximum tolerated dose (0.265 log mg per kg per day) together with the oral rat acute (LD_50_ = 2.917 mol kg^−1^) and chronic toxicity values (LOAEL = 1.214 log mg per kg per bw per day) suggested acceptable systemic tolerance. Aquatic toxicity estimates (*T. pyriformis* = 0.504 log µg per L; minnow toxicity = 0.717 log mM) were also low, reflecting minimal environmental risk (Table S3). ProTox-III predictions corroborated the pkCSM results. Compound 6 was classified as inactive toward major organ- and cellular-toxicity endpoints, including hepatotoxicity, cardiotoxicity, carcinogenicity, mutagenicity, and general cytotoxicity. All major CYP450 isoenzymes (CYP1A2, 2C19, 2C9, 2D6, 3A4, 2E1) were predicted as non-inhibited, implying a low risk of metabolic interactions or bioactivation into toxic intermediates. A single moderate-probability alert for immunotoxicity (0.53) suggested a potential mild immunomodulatory effect, which may merit targeted *in vitro* follow-up but does not indicate overt immune toxicity (Table S4). Overall, the combined pkCSM–ProTox-III data demonstrate a favorable toxicity and off-target profile for compound 6, aligning with its high cellular selectivity index and the absence of significant hepato-renal alterations observed *in vivo*. These findings support the compound's broad therapeutic safety window.

## 3 Materials and methods

### 3.1 Chemical synthesis and characterization

#### 3.1.1. Synthesis of 2-(5-chloro-2-hydroxybenzylidene)hydrazineylidene-thiazolidin-4-one (2)

A solution of (*E*)-2-(5-chloro-2-hydroxybenzylidene)hydrazine-1-carbothioamide 1 (ref. 36) (3.2 g, 14.2 mmol) in dry ethanol (30 mL) was treated with anhydrous K_2_CO_3_ (5.7 g, 42.6 mmol), followed by ethyl chloroacetate (1.8 mL, 17.1 mmol). The reaction mixture was allowed to stir under reflux at 85 °C, and the reaction progress was followed by TLC analysis. After being refluxed for 12 hours, the TLC analysis revealed a complete reaction. Subsequently, the mixture was cooled in ice-cold water and quenched with water. The mixture was stirred at 0 °C for 30 min, resulting in the formation of a solid precipitate. The mixture was filtered, and the obtained solid was washed with water and dried to provide a crude product as a pale-yellow solid. The crude product was further purified by recrystallization in ethanol to afford compound 2 as colorless crystals. Yield: 71% (2.7 g), m. p. 225 °C. IR *ν*_max_: 3450–3390 (br-OH), 3226 (NH), 1705 (C=O), 1633 (C=N), 1605, 1589 (C=C), 1087, 1033 (C–O) cm^−1^. ^1^H-NMR (DMSO-*d*_6_, 400 MHz, ppm): *δ* = 12.10 (br. s, 1H, OH), 10.95 (s, 1H, NH), 8.61 (s, 1H, CH=N), 7.66 (s, 1H, H-aromatic), 7.34–7.37 (d, 1H, H-aromatic), 6.97–6.99 (d, 1H, H-aromatic), 3.98 (s, 2H, CH_2_ of thiazolone ring). ^13^C-NMR (DMSO-*d*_6_, 100 MHz, ppm): *δ* 178.3 (C-2 of thiazolone ring), 166.0 (C=O), 157.1 (C=N), 156.0 (C–O), 137.8, 132.0, 130.8, 128.8, 125.9 (C-aromatic), 33.9 (CH_2_ of thiazolone ring). ESI-MS (EI, 70 eV) *m*/*z* (%): 269 (37.01), 251 (31.40), 219 (10.13), 155 (37.21), 152 (28.19), 139 (57.85), 125 (23.74), 115 (12.43), 111 (62.80), 100 (26.18), 99 (44.30), 75 (100), 74 (71.12), 63 (30.42), 62 (70.83). Anal. calcd. for C_10_H_8_ClN_3_O_2_S (M. wt = 269): C, 44.53; H, 2.99; N, 15.58. Found: C, 44.33; H, 2.89; N, 15.48.

#### 3.1.2. Synthesis of 2-((3-acetyl-4-oxothiazolidin-2-ylidene)hydrazineylidene)methyl-4-chlorophenyl acetate (3)

Compound 2 (700 mg, 2.6 mmol) was dissolved in acetic anhydride (15 mL), and the obtained mixture was allowed to reflux at 150 °C for 16 h. After the TLC analysis indicated a complete reaction, the reaction was cooled in ice-cold water and quenched with water. Stirring the mixture at 0 °C for 30 min produced a solid precipitate, which was subsequently filtered and washed with water. The obtained crude product was further purified by recrystallization using ethanol to provide compound 3 as a yellow solid. Yield: 88% (810 mg), m. p. 168 °C. IR *ν*_max_: 1753–1748 (br. C=O of ester), 1713 (C=O), 1633 (C=N), 1608, 1592 (C=C), 1123, 1082, 1036 (C–O) cm^−1^. ^1^H-NMR (DMSO-*d*_6_, 400 MHz, ppm) *δ*: 8.56 (s, 1H, CH=N), 7.70 (s, 1H, H-aromatic),6.96–6.98 (d, 1H, H-aromatic), 6.73–6.78 (d, 1H, H-aromatic), 3.94 (s, 2H, CH_2_ of thiazolone ring), 1.91 (s, 3H, OCOCH_3_), 1.78 (s, 3H, COCH_3_). ^13^C-NMR (DMSO-*d*_6_, 100 MHz, ppm) *δ*: 176.9, 172.2, 169.2, 166.0, 163.5 (3C=O, 2C=N and C–O), 157.1, 154.9, 152.4, 147.9, 130.6, 129.3 (C-aromatic and olefinic), 34.5 (CH_2_ of thiazolone ring), 22.9, 21.6 (2CH_3_). ESI-MS (EI, 70 eV) *m*/*z* (%): 355 (8.48), 311 (19.07), 269 (96.58), 268 (37.80), 251 (100), 153 (35.87), 116 (42.85). Anal. calcd. for C_14_H_12_ClN_3_O_4_S (M. wt = 353): C, 47.53; H, 3.42; N, 11.88. Found: C, 47.33; H, 3.32; N, 11.78.

#### 3.1.3. Synthesis of 2-((-3-bromo-5-chloro-2-hydroxybenzylidene)-hydrazineylidene)-thiazolidin-4-one (4)

Compound 2 (1.2 g, 4.5 mmol) was dissolved in 25 mL of glacial acetic acid, and the resulting solution was allowed to heat to 60 °C. Subsequently, the reaction was carefully treated with bromine (0.5 mL, 8.9 mmol), during which the color changed to yellow. The Br_2_ color disappeared after five to ten minutes, leaving only a yellow solution. At this stage, 1 mL of the Br_2_-AcOH solution was added, and it was left stirring for 16 h at room temperature. After swirling the reaction into freezing H_2_O and filtering off the solid, it was rinsed with H_2_O and dried. Crystallization of the final product from ethanol afforded the desired compound 4 as pale-yellow crystals. Yield 62% (970 mg), m. p. 246 °C. IR *ν*_max_: 3485–3405 (br. OH), 3222 (NH), 1711 (C=O), 1635 (C=N), 1610, 1590 (C=C), 1068, 1031 (C–O) cm^−1^. ^1^H-NMR (DMSO-*d*_6_, 400 MHz, ppm): *δ* = 11.00 (s, 1H, OH), 10.86 (s, 1H, NH), 8.47 (s, 1H, CH=N), 7.90 (s, 1H, H-aromatic), 7.59 (s, 1H, H-aromatic), 3.90 (s, 2H, CH_2_ of thiazolone ring).^13^C-NMR (DMSO-*d*_6_, 100 MHz, ppm): *δ* 181.3 (C-2 of thiazolone ring), 180.1 (C=O), 172.5 (C=N), 170.3 (C–O), 152.4, 151.5, 144.8, 138.4, 135.4 (C-aromatic), 21.5 (CH_2_ of thiazolone ring). Anal. calcd. for C_10_H_7_BrClN_3_O_2_S (M. wt = 346): C, 34.46; H, 2.02; N, 12.05. Found: C, 34.26; H, 1.97; N, 11.90.

#### 3.1.4. Synthesis of 2-((-3-acetyl-4-oxothiazolidin-2-ylidene)-hydrazineylidene-methyl)-6-bromo-4-chlorophenyl acetate (5)

A stirred solution of compound 4 (600 mg, 1.73 mmol) in acetic anhydride (7 mL) was allowed to reflux at 150 °C, and the reaction progress was followed by TLC analysis. After being stirred at 150 °C for 18 h, TLC analysis indicated a complete reaction. The reaction was allowed to cool to 0 °C and subsequently quenched with water. The resulting mixture was stirred under the same conditions for 30 min, resulting in the formation of a solid precipitate, which was filtered. The obtained solid was washed with water and dried to afford a yellow residue. Finally, compound 5 was obtained as yellow crystals by crystallizing the residue from ethanol. Yield: 74% (550 mg), m. p. 206 °C. IR *ν*_max_: 1701 (C=O), 1615 (C=N), 1605, 1580 (C=C), 1058, 1021 (C–O) cm^−1^. ^1^H-NMR (DMSO-*d*_6_, 400 MHz, ppm): *δ* = 9.04 (s, 1H, CH=N), 7.00–7.25 (m, 2H, H-aromatic), 4.55 (s, 2H, CH_2_ of thiazolone ring), 1.77 (s, 3H, OCOCH_3_), 1.24 (s, 3H, COCH_3_). ^13^C-NMR (DMSO-*d*_6_, 100 MHz, ppm): *δ* 173.3 (C-2 of thiazolone ring), 172.4, 167.2, 158.6 (3C=O), 157.7 (C=N), 149.6 (C–O), 134.9, 133.2, 131.7, 128.8, 126.2 (C-aromatic), 36.1 (CH_2_ of thiazolone ring), 31.3, 24.8 (2CH_3_). Anal. calcd. for C_14_H_11_BrClN_3_O_4_S (M. wt = 430): C, 38.86; H, 2.56; N, 9.71. Found: C, 38.76; H, 2.46; N, 9.61.

#### 3.1.5. Synthesis of methyl 3-2-((5-chloro-2-hydroxybenzylidene)-hydrazineylidene)-4-oxothiazolidin-3-yl-propanoate (6)

A solution of thiazol-4-one 2 (1.6 g, 6 mmol) in dry DMF (30 mL) at ambient temperature was treated with triethylamine (2 mL), followed by methyl acrylate (0.6 mL, 6.6 mmol). The obtained mixture was allowed to reflux at 155 °C, and the reaction progress was followed by TLC analysis. After being stirred for 14 h under the same conditions, TLC indicated a complete reaction. The reaction was subsequently cooled to 0 °C and carefully quenched with 2% HCl solution. The mixture was extracted with diethyl ether, and the combined organic layers were washed with water, followed by brine solution. After the resulting solution was dried over anhydrous sodium sulphate and filtered, it was concentrated under reduced pressure to provide a yellow solid residue. Purification of the crude residue by recrystallization from ethanol yielded the desired product 6 as a pale yellow solid. Yield 79% (1.58 g), m. p. 226 °C. IR *ν*_max_: 3425 (OH), 1690 (C=O), 1615 (C=N), 1580, 1610 (C=C), 1058, 1021 (C–O) cm^−1^. ^1^H-NMR (DMSO-*d*_6_, 400 MHz, ppm): *δ* = 10.88 (s, 1H, OH), 8.69 (s, 1H, CH=N), 7.68 (s, 1H, H-aromatic), 7.36–7.39 (d, 1H, H-aromatic), 6.97–7.00 (d, 1H, H-aromatic), 4.03 (s, 2H, CH_2_ of thiazolone ring), 3.95–3.99 (t, 2H, CH_2_), 3.36 (s, 3H, OCH_3_), 2.70–2.74 (t, 2H, CH_2_). ^13^C-NMR (DMSO-*d*_6_, 100 MHz, ppm): *δ* 174.1 (C-2 of thiazolone ring), 164.8 (C=O), 163.0 (C=N), 157.1 (C–O), 132.3, 131.9, 128.96, 123.6, 120.7, 118.8 (C-aromatic), 60.0 (OCH_3_), 34.0, 32.9, 31.6 (3CH_2_). ESI-MS (EI, 70 eV) *m*/*z* (%): 355 (23.91), 323 (12.25), 268 (37.08), 251 (100). Anal. calcd. for C_14_H_14_ClN_3_O_4_S (M. wt = 355): C, 47.26; H, 3.97; N, 11.81. Found: C, 47.16; H, 3.87; N, 11.71.

#### 3.1.6. Synthesis of methyl 3-(2-((2-acetoxy-5-chlorobenzylidene)hydrazineylidene)-4-oxothiazolidin-3-yl)propanoate (7)

Compound 6 (500 mg, 1.4 mole) was treated with acetic anhydride (10 mL), and the resulting mixture was heated at 150 °C. The reaction progress was followed by TLC analysis, which showed a complete reaction after 12 h. The mixture was allowed to cool to 0 °C and then carefully quenched with water. The obtained mixture was extracted with dichloromethane, and the combined layers were subsequently washed with brine and dried over anhydrous sodium sulphate. The residue obtained after concentration was finally purified by recrystallization from ethanol to provide compound 7 as a yellow solid. Yield: 68% (378 mg), m. p. 206 °C. IR *ν*_max_: 1680 (C=O), 1610 (C=N), 1600, 1570 (C=C), 1048, 1011 (C–O) cm^−1^. ^1^H-NMR (DMSO-*d*_6_, 400 MHz, ppm): *δ* = 8.66 (s, 1H, CH=N), 7.65 (s, 1H, H-aromatic), 7.28–7.30 (d, 1H, H-aromatic), 6.98–7.00 (d, 1H, H-aromatic), 3.99 (s, 2H, CH_2_ of thiazolone ring), 3.87–3.97 (t, 2H, CH_2_), 3.40 (s, 3H, COCH_3_), 2.70–2.71 (t, 2H, CH_2_), 1.76 (s, 3H, OCOCH_3_). ^13^C-NMR (DMSO-*d*_6_, 100 MHz, ppm): *δ* 177.4 (C-2 of thiazolone ring), 174.1 (C=O), 172.8 (C=N), 171.5 (C–O), 164.2, 158.4, 156.2, 132.1, 127.7, 122.5, 121.2, 119.3 (C-aromatic), 63.2 (OCH_3_), 32.8, 31.6 (2CH_2_), 21.5 (COCH_3_). Anal. calcd. for C_14_H_11_BrClN_3_O_4_S (M. wt = 430): C, 38.86; H, 2.56; N, 9.71. Found: C, 38.76; H, 2.46; N, 9.61.

### 3.2 *In vitro* biological assessments

#### 3.2.1. Cell growth inhibition assay

The cytotoxic potential of the synthesized compounds was evaluated using the MTT colorimetric assay. Human breast cancer cell lines MCF-7 and MDA-MB-231, along with the non-tumorigenic breast epithelial cell line MCF-10A (obtained from the Regional Center for Mycology and Biotechnology at Al-Azhar University, Cairo, Egypt), were used as representative models. Briefly, cells were seeded in 96-well plates at a density of 1 × 10^4^ cells per well and allowed to adhere for 24 h under standard culture conditions (37 °C, 5% CO_2_, humidified atmosphere). After incubation, cells were treated with increasing concentrations of the test compounds, which were freshly prepared in DMSO and diluted in culture medium to ensure a final solvent concentration of <0.5% (non-cytotoxic). Doxorubicin was included as a positive control. Following 48 h of exposure, the treatment medium was replaced with 20 µL of MTT solution (5 mg mL^−1^ in PBS), and plates were incubated for an additional 4 h to allow mitochondrial reduction of MTT to insoluble formazan crystals. The crystals were then dissolved in 100 µL of DMSO, and absorbance was measured at 570 nm using a microplate reader. Cell viability was calculated relative to untreated controls, and IC_50_ values (concentration required to inhibit 50% of cell growth) were determined using nonlinear regression analysis in GraphPad Prism (GraphPad Software, USA). All experiments were performed in triplicate and repeated three times independently, with data expressed as mean ± SD.^50,51^

#### 3.2.2. Flow cytometric analysis

Flow cytometry was employed to evaluate the effects of compound 6 on cell cycle distribution and apoptosis induction in MDA-MB-231 cells. The effect of compound 6 on cell cycle progression was assessed using propidium iodide (PI) staining. Briefly, MDA-MB-231 cells were seeded at a density of 2 × 10^5^ cells per well and allowed to adhere overnight under standard conditions (37 °C, 5% CO_2_). Cells were then treated with compound 6 at its IC_50_ concentration (2.25 µM) for 24 h, while vehicle-treated cells (0.5% DMSO) served as controls. Following treatment, cells were harvested, washed twice with cold PBS, and fixed in 70% ethanol at −20 °C for 2 h. Fixed cells were washed, resuspended in PI/RNase staining solution, and incubated for 30 min at 37 °C in the dark. DNA content was analyzed using a BD FACSCalibur™ flow cytometer, and PI fluorescence was detected at 585 nm. Cell cycle distribution across the G_0_/G_1_, S, and G_2_/M phases was determined. To evaluate apoptosis, Annexin V-FITC/PI double staining was performed. MDA-MB-231 cells were seeded at 2 × 10^5^ cells per well and incubated overnight. Cells were then treated with compound 6 at its IC_50_ concentration (2.25 µM) for 24 h, while vehicle-treated cells (0.5% DMSO) served as controls. After treatment, both floating and adherent cells were collected, washed with cold PBS, and resuspended in 500 µL of Annexin V binding buffer. Subsequently, 5 µL of Annexin V-FITC and 5 µL of PI were added, and cells were incubated for 15 min at room temperature in the dark. Samples were immediately analyzed by BD FACSCalibur™ flow cytometer, with Annexin V detected at 530 nm and PI at 585 nm. This assay enabled discrimination between viable cells, early apoptotic cells, late apoptotic cells, and necrotic/dead cells.^52,53^

#### 3.2.3. Quantification of caspase-3 and caspase-7

To determine whether compound 6 activates apoptotic pathways, the expression of caspase-3 and caspase-7 was quantified in MDA-MB-231 cells using ELISA-based detection kits. Active caspase-3 levels were measured with the Human Active Caspase-3 ELISA kit (Invitrogen, Cat. No. KHO1091), while caspase-7 expression was assessed with the Human Caspase-7 ELISA kit (BioVision, Cat. No. E4295-100). Briefly. MDA-MB-231 cells were seeded in 96-well plates (5 × 10^5^ cells per well) and treated with compound 6 at its IC_50_ concentration for 24 h, while untreated cells and doxorubicin served as controls. After treatment, cells were harvested, lysed in extraction buffer supplemented with protease inhibitors, clarified by centrifugation (13 000 rpm, 10 min, 4 °C) and kept on ice (single freeze–thaw avoided). Aliquots of cell lysates and standards were added to antibody-coated wells (100 µL per well) and incubated according to the manufacturer's protocol, followed by sequential addition of biotinylated detection antibody and HRP-conjugate. The enzymatic reaction was visualized with TMB substrate, stopped with sulfuric acid, and the absorbance was measured at 450 nm using a microplate reader. Caspase activities were calculated by interpolating absorbance values against the standard curves, and results were expressed as mean ± SD from triplicate independent experiments.^54,55^

#### 3.2.4. Aromatase inhibition assay

Aromatase (CYP19A1) inhibition by compound 6 was assessed in parallel with letrozole (reference) using a fluorometric 96-well plate assay (BioVision, K983-100). Stock solutions were prepared in DMSO and serially diluted in assay buffer to 5–100 nM (final DMSO ≤ 0.5%). In black 96-well plates, recombinant human aromatase was combined with assay buffer and NADPH-generating components; test or vehicle solutions were added and pre-incubated for 10 min at 37 °C to allow inhibitor–enzyme equilibration. Reactions were initiated by adding the fluorogenic substrate and monitored at Ex/Em 488/527 nm in kinetic mode for 60 min at 37 °C. Each plate included vehicle control (enzyme + DMSO), a no-enzyme blank (background), and the letrozole reference at the same 5–100 nM range. For each well, background-subtracted rates (ΔRFU/Δ*t*) were calculated over the linear phase; % inhibition = 100 × [1 − (rate_sample/rate_vehicle)]. IC_50_ values for compound 6 and letrozole were obtained using GraphPad Prism. All conditions were run in triplicate and repeated in three independent experiments; data are reported as mean ± SD.^56,57^

#### 3.2.5. Steroid sulfatase inhibition assay

The inhibitory activity of compound 6 against steroid sulfatase was evaluated using the Human STS Chemiluminescent Immunoassay Kit (LSBio, Cat. No. LS-F29115), with danazol as the reference inhibitor. Compounds were dissolved in DMSO (final DMSO concentration <0.5%) and serially diluted (100–5 µM). Briefly, 100 µL of each sample, standard, or blank was added to antibody-coated wells and incubated at 37 °C for 90 min. After aspiration, 100 µL of the biotinylated detection antibody (1× dilution) was added and incubated for 60 min at 37 °C, followed by three washes with wash buffer. Then, 100 µL of the HRP–avidin conjugate (1× dilution) was added and incubated for 30 min at 37 °C, followed by five washes. Next, 100 µL of freshly prepared chemiluminescent substrate solution (substrate reagent A + B, 1 : 1) was added and incubated for 5 min at 37 °C in the dark. Relative light units (RLU) were measured immediately using a luminometer. A standard curve was generated from serial dilutions of the supplied recombinant STS standard (62.5–4000 pg mL^−1^), and sample values were interpolated accordingly. STS inhibition by test compounds was calculated relative to untreated controls. All assays were performed in triplicate, and results are expressed as mean ± SD.^58^

#### 3.2.6. DPPH free radical scavenging assay

The antioxidant potential of compound 6 was evaluated using the 2,2-diphenyl-1-picrylhydrazyl (DPPH) assay. A 0.1 mM DPPH solution was prepared in ethanol, and 1 mL of this solution was added to 3 mL of compound 6 solutions prepared in DMSO and diluted to final concentrations ranging from 2.5–60 µM. The mixtures were vigorously shaken and incubated at room temperature for 30 min in the dark, after which absorbance was measured at 517 nm using a UV-Vis spectrophotometer. Ascorbic acid (2.5–60 µM) served as the reference antioxidant. All experiments were performed in triplicate, and results were expressed as mean ± SD. The percentage of radical scavenging activity was calculated using the following equation: Percent inhibition or DPPH scavenging action (%) = *A*_0_ − *A*_1_/*A*_0_ × 100. *A*_1_ was the absorbance in the presence of a test or standard sample, and *A*_0_ was the absorbance of the control reaction. The IC_50_ value was determined from the dose–response curve.^59^

#### 3.2.7. Cyclooxygenase activity inhibition

COX inhibition was assessed using BioVision COX-1 Inhibitor Screening Kit (K548-100) and COX-2 Inhibitor Screening Kit (K547-100), which quantify conversion of arachidonic acid to PGG_2_*via* a fluorogenic probe (Ex/Em 535/587 nm) in 96-well plates. Test solutions of compound 6 and celecoxib (reference inhibitor for both isoforms) were prepared in DMSO and diluted in COX assay buffer to the desired concentrations (final DMSO ≤ 0.5%). For each isoform, a per-well reaction master mix was assembled on ice (76 µL assay buffer + 1 µL COX probe + 2 µL 200× COX cofactor + 1 µL recombinant COX-1 or COX-2; total 80 µL), followed by 10 µL of compound 6, celecoxib, or vehicle solution. Reactions were initiated with 10 µL freshly prepared arachidonic acid/NaOH (activated AA per kit instructions) and read kinetically for 30 min at 25 °C. Background-subtracted slopes (ΔRFU/Δ*t*) were used to calculate % inhibition. IC_50_ values for compound 6 and celecoxib were determined utilizing GraphPad Prism. All conditions were run in triplicate, and results were reported as mean ± SD.^60^

### 3.3 *In silico* computational analysis

#### 3.3.1. Molecular docking analysis

Molecular docking simulations were performed to evaluate the binding affinity and interaction profiles of compound 6 against multiple protein targets relevant to cancer progression and inflammation. Based on prior biochemical findings, the following targets were selected: aromatase (PDB ID: 3EQM), steroid sulfatase (STS; PDB ID: 1P49), cyclooxygenase-1 (COX-1; PDB ID: 1EQG), cyclooxygenase-2 (COX-2; PDB ID: 5KIR), Vascular Endothelial Growth Factor Receptor-2 (VEGFR2; PDB ID: 2OH4), and Tumor Necrosis Factor-α (TNF-α; PDB ID: 2AZ5). The 3D structure of compound 6 was energy-minimized and prepared using AutoDockTools, while docking simulations were carried out with AutoDock4 employing the Lamarckian genetic algorithm. For each protein, the docking grid box was centered on the active site, covering key catalytic residues. To validate the docking protocol, co-crystallized ligands were re-docked into their respective binding sites, and the accuracy was confirmed by calculating the root mean square deviation (RMSD) between the predicted and experimental poses. Docking outputs were ranked by binding energy, and the most favorable poses were analyzed for hydrogen bonding, hydrophobic contacts, and π-interactions using Discovery Studio Visualizer.^61–63^

#### 3.3.2. Molecular dynamics simulations

Molecular dynamics simulations were carried out using GROMACS 2023.1 to evaluate the stability and dynamic behavior of the ligand–protein complexes over 100 ns. Protein topologies were generated with the CHARMM36 force field, while ligand parameters were prepared using the CGenFF server. The complexes were solvated in a dodecahedral water box with periodic boundary conditions and a 10 Å buffer distance from the solute to the box edge. Neutralization was achieved by adding appropriate numbers of Na^+^ and Cl^−^ ions. Energy minimization was performed using the steepest descent algorithm (maximum 50 000 steps, force convergence threshold 10 kJ mol^−1^) to relieve steric clashes. System equilibration was conducted in two phases: *NVT* (constant volume and temperature) and *NPT* (constant pressure and temperature), each for 50 000 steps (∼100 ps total), using a modified Berendsen thermostat and the leap-frog integrator. The production MD run was performed for 100 ns with a 2 fs time step, applying periodic boundary conditions throughout. Structural stability and interaction persistence were analyzed using RMSD, RMSF, radius of gyration (*R*_g_), solvent accessible surface area (SASA), and hydrogen bond profiles. For binding free-energy estimation, the MM/GBSA approach was applied using the gmx_MMPBSA v1.4.1 package, which implements the MMPBSA.py framework, to calculate the average free energy and per-residue decomposition across the trajectory.^53,57,64^

#### 3.3.3. Principal component and free energy landscape analysis

Principal component analysis (PCA) was carried out to characterize the dominant collective motions of the targeted protein–compound 6 complex following the 100 ns MD simulations. For every target, the equilibrated portion of the trajectory was corrected for periodic boundary conditions, centered, and aligned to the initial minimized structure using Cα atoms to eliminate overall translational and rotational movements. The positional fluctuations of the aligned coordinates were used to construct the covariance matrix of atomic displacements, which was subsequently diagonalized to yield eigenvalues and eigenvectors describing the amplitude and direction of the major internal motions. The first two principal components (PC1 and PC2), accounting for the largest fraction of the total variance, were extracted and used to project the trajectories and visualize the conformational space sampled by the complexes. Free energy landscapes (FELs) were generated by mapping the Gibbs free energy onto the PC1–PC2 space to identify dominant conformational basins and assess the stability of ligand-bound states. All PCA and FEL computations were performed using GROMACS utilities (gmx covar and gmx anaeig) to ensure consistent treatment of the six protein–ligand systems.

### 3.4 Assessments of antitumor potential in the EAC-induced animal model

#### 3.4.1. Animals and housing conditions

Swiss albino female mice (20–25 g) were obtained from the breeding colony at Abo Rawash, Giza, Egypt. The animals were maintained in the animal facility of the Faculty of Science, Port Said University, in mesh-type stainless steel cages under controlled laboratory conditions (temperature 22 ± 2 °C; 12-h light/dark cycle). Standard rodent chow and water were available without restriction. Prior to the experimental procedures, mice were acclimatized for one week. All protocols were conducted in accordance with institutional animal welfare regulations and received approval from the Animal Ethics Committee of Port Said University (ERN: PSU.Sci.15).

#### 3.4.2. Ehrlich ascites carcinoma (EAC) cell quantification and viability testing

EAC cells were supplied by the National Cancer Institute, Cairo, and propagated into mice through serial intraperitoneal transplantation at 10-day intervals. For experimental use, ascitic fluid was withdrawn under sterile conditions using disposable syringes, and the collected volume was recorded. Cell viability was evaluated using the trypan blue dye-exclusion method.^65^ In brief, a portion of the ascitic suspension was diluted with sterile physiological saline to achieve the desired cell concentration. A 10 µL sample of the diluted suspension was then mixed with an equal volume of 0.4% trypan blue solution, gently homogenized, and left to stand for 2–3 minutes at ambient temperature. The stained preparation was subsequently loaded onto a Neubauer hemocytometer and examined microscopically (40×). Each measurement was carried out in triplicate, and results were expressed as mean ± SD.^66,67^

#### 3.4.3. Acute toxicity and optimal therapeutic dose determination

Modified Meier–Theakston procedure was utilized to assess the lethal dose of compound 6. Groups of mice (*n* = 4 per dose) received intraperitoneal injections of escalating concentrations of the compound (1–200 mg kg^−1^). In the initial phase (1–10 mg kg^−1^), animals were monitored for 24 h, during which no mortality was observed. A subsequent phase employed higher doses (15–200 mg kg^−1^) with a 48-h observation period. Mortality, behavioral alterations, and clinical signs of toxicity were documented. The non-lethal maximum dose (LD_0_) and the dose causing complete lethality (LD_100_) were established and used to guide subsequent efficacy studies.^68^ For the determination of the therapeutic dose, Swiss albino mice were inoculated intraperitoneally with 2.5 × 10^6^ viable EAC cells (day 1). Animals were randomly allocated into six groups (*n* = 6 per group): one served as the untreated EAC control, while the others received compound 6 at doses ranging from 2.5 to 20 mg kg^−1^ i. p. Treatments were administered every other day for a total of five injections over 10 days. On day 11, ascitic fluid was aspirated, and viable tumor cells were quantified using the trypan blue exclusion method. All experiments were performed in triplicate and repeated independently three times. Data were analyzed with GraphPad Prism software, and differences were considered significant at *p* < 0.05.^69^

#### 3.4.4. Experimental design, treatment, and tissue collection

Twenty-four mice were randomly distributed into four groups (*n* = 6 each) based on dose–response outcomes. Group 1 (untreated control) received sterile saline only. Group 2 (compound 6 control) was administered compound 6 at 15 mg kg^−1^ intraperitoneally (i. p.) in the absence of tumor inoculation. Group 3 (EAC-treated group) was inoculated with 2.5 × 10^6^ viable EAC cells without further intervention. Group 4 (compound 6 EAC-induced group) received the same EAC inoculum, followed by compound 6 (15 mg kg^−1^, i. p.) administered on alternate days for a total of five doses. On day 11, ascitic fluid was aspirated for tumor cell enumeration. Blood samples were collected from the retro-orbital venous plexus under anesthesia induced by intraperitoneal injection of thiopental sodium (20 mg kg^−1^). At the end of the experiment, animals were deeply anesthetized with thiopental sodium (20 mg kg^−1^, i. p.) and humanely euthanized by cervical dislocation while fully unconscious, in accordance with the NIH Guide for the Care and Use of Laboratory Animals (8th edition) and the institutional ethical approval for this study. The liver and kidneys were excised, rinsed with chilled phosphate-buffered saline (PBS), and divided into two portions: one stored at −20 °C for biochemical analysis and the other fixed in 10% neutral-buffered formalin for histopathological examination.^63,70^

#### 3.4.5. Quantification of serum ALT and AST markers

Serum activities of alanine aminotransferase (ALT; SGPT) and aspartate aminotransferase (AST; SGOT) were determined using commercial colorimetric assay kits (TECO Diagnostics, ALT Cat. A526; AST Cat. A561). Both assays employ modified endpoint methods: ALT catalyzes the transamination of l-alanine with α-ketoglutarate to yield pyruvate, which forms a hydrazone with 2,4-dinitrophenylhydrazine and is detected spectrophotometrically at 505 nm. In the AST assay, l-aspartate is transaminated with 2-oxoglutarate to form oxaloacetate, which reacts with a diazonium salt, producing a chromogen measurable at 530 nm. For each measurement, 0.1 mL of serum was incubated with pre-warmed substrate at 37 °C (30 min for ALT and 10 min for AST). Following the addition of the color reagent, the ALT reaction was developed for 5 min, whereas the AST reaction was terminated with 0.1 N HCl. Absorbance values were recorded within 60 min using a microplate reader. Enzyme activity (U L^−1^) was calculated against kit calibrators. Samples exceeding the linear range were reanalyzed after appropriate dilution (ALT: 1 : 5; AST: 1 : 10). All assays were conducted in triplicate, and results are reported as mean ± SD.^71,72^

#### 3.4.6. Quantifications of serum creatinine and urea markers

Renal function was assessed by determining serum creatinine and urea using commercial colorimetric kits (Abcam; creatinine: ab65340, urea: ab83362). The creatinine assay is based on a two-step enzymatic conversion to sarcosine, followed by oxidation that generates a chromogenic product measurable at 570 nm. In practice, serum (10 µL) was incubated with the reaction mixture at 37 °C for 30 min, and absorbance was recorded using a microplate reader. Urea was quantified through urease-mediated hydrolysis to ammonia and carbon dioxide, with the released ammonia reacting with a probe to yield a colorimetric signal at 570 nm. For this assay, serum (5 µL) was mixed with the working reagent, incubated at 37 °C for 20 min, and the absorbance was measured. Concentrations were calculated from standard calibration curves and expressed in mg dL^−1^. All measurements were carried out in triplicate, and results are presented as mean ± SD.^30,31^

#### 3.4.7. Quantification of oxidative stress markers

##### 3.4.7.1 Evaluation of SOD activity

Following the manufacturer's protocol, SOD1 activity in liver tissue homogenates was quantified using a commercial ELISA kit (Abcam, Cat. No. ab119520). In brief, liver homogenate samples and standards were applied to wells pre-coated with monoclonal anti-SOD1 antibodies, followed by incubation with an HRP-conjugated detection antibody. After successive wash steps to eliminate non-specific binding, TMB substrate was added, producing a blue chromogenic signal that was converted to yellow upon addition of the stop solution. Absorbance was recorded at 450 nm using a microplate reader, and SOD1 concentrations were interpolated from a standard curve. Each sample was analyzed in triplicate, and results are expressed as mean ± SD.^73^

##### 3.4.7.2 Evaluation of GSH activity

Following the manufacturer's protocol, hepatic GSH levels were quantified using a commercial ELISA kit (MyBioSource, Cat. No. MBS265674). Briefly, diluted liver homogenate samples and calibration standards were added to 96-well plates pre-coated with GSH-specific monoclonal antibodies and incubated at 37 °C for 1 h to allow antigen–antibody binding. After washing to eliminate unbound material, an HRP-conjugated detection antibody was introduced, followed by a 30-min incubation at 37 °C. Subsequent addition of TMB substrate generated a chromogenic signal, which was terminated by an acidic stop solution, producing a yellow endpoint. Optical density was measured at 450 nm using a microplate reader. In this competitive assay, absorbance values were inversely related to GSH concentration, which was calculated from a standard curve prepared with known concentrations. Each sample was assayed in triplicate, and data are presented as mean ± SD.^74^

##### 3.4.7.3 Evaluation of MDA activity

Following the manufacturer's protocol, hepatic MDA content, a marker of lipid peroxidation, was quantified using a competitive ELISA kit (MyBioSource, USA; Cat. No. MBS268427). Briefly, prepared liver homogenates and standard solutions were added to wells pre-coated with MDA-specific monoclonal antibodies and incubated to permit antigen binding. After removal of unbound material by washing, a biotin-labeled detection antibody was applied, followed by an avidin–HRP conjugate. Color development was achieved with TMB substrate, producing a chromogenic signal proportional to MDA concentration, and the reaction was terminated with an acidic stop solution. Absorbance was measured at 450 nm using a microplate reader, and MDA levels were calculated from a standard curve and expressed as nmol per gram of tissue. All assays were performed in triplicate, and results are presented as mean ± SD.^75^

##### 3.4.7.4 Evaluation of CAT activity

Following the manufacturer's protocol, CAT activity in liver homogenates was determined using a commercial ELISA kit (MyBioSource, Cat. No. MBS006963). Briefly, liver tissues were homogenized in assay buffer, and the supernatant was collected after centrifugation to remove cellular debris. Prepared extracts and standards were then loaded into 96-well plates pre-coated with catalase-specific antibodies and incubated at 37 °C to facilitate antigen–antibody interaction. After washing, a biotinylated detection antibody was applied, followed by an HRP-conjugated secondary reagent. The enzymatic reaction was initiated with TMB substrate and stopped with sulfuric acid, producing a stable yellow color. Absorbance was measured at 450 nm using a microplate reader, and catalase activity was calculated from a standard curve and normalized to tissue weight, expressed as units per gram. Each sample was analyzed in triplicate, and values are reported as mean ± SD.^76^

#### 3.4.8. Quantification of serum inflammatory and angiogenic markers

Serum concentration of TNF-α was measured using a mouse-specific ELISA kit (Invitrogen™, Cat. No. BMS607-3) according to the manufacturer's protocol. Briefly, serum samples and standards were added to 96-well plates pre-coated with capture antibodies against TNF-α and incubated to permit antigen binding. Following removal of unbound components by washing, a biotinylated detection antibody was applied, followed by avidin–HRP conjugate. Color development was achieved using TMB substrate and terminated with an acidic stop solution. Absorbance was recorded at 450 nm using a microplate reader, and TNF-α concentrations were interpolated from a standard calibration curve.^77^ On the other hand, serum concentrations of VEGFR-2 were quantified using a sandwich ELISA kit (Cusabio®, Cat. No. CSB-E07348r) following the manufacturer's protocol. Briefly, serum samples were added to 96-well plates pre-coated with monoclonal antibodies specific for VEGFR-2 and incubated to allow antigen–antibody binding. After washing to remove unbound material, a biotin-labeled detection antibody was applied, followed by avidin–HRP conjugate. Color development was initiated with TMB substrate and terminated by the addition of an acidic stop solution. Absorbance was measured at 450 nm using a microplate reader, and VEGFR-2 concentrations were calculated from a standard curve generated by logistic regression. Each sample was assayed in triplicate, and results are expressed as mean ± SD.^78^

#### 3.4.9. Histological analysis (hematoxylin and eosin staining)

At the time of sacrifice, liver and kidney tissues were excised and immediately fixed in 10% neutral-buffered formalin. After fixation, samples were dehydrated in ascending ethanol series, cleared in xylene, and embedded in paraffin wax. Paraffin blocks were sectioned at 5 µm thickness using a rotary microtome (Shandon Finesse, Thermo Fisher Scientific, USA), mounted on charged glass slides, and stained with hematoxylin and eosin (H&E) for general histopathological assessment.^79–81^ Renal sections were examined for features of acute tubular injury (ATI), including tubular epithelial cell flattening, cytoplasmic vacuolization, necrosis, and loss of brush-border integrity, and graded using a semi-quantitative injury scoring system.^82^ Hepatic sections were assessed according to a modified Brunt classification, with emphasis on hepatocyte ballooning, inflammatory cell infiltration, necrotic foci, and degenerative alterations. Microscopic evaluation was performed using a Zeiss Axioscope 5 microscope (Carl Zeiss Microscopy, Germany) equipped with an Axiocam 305 digital camera for image acquisition and documentation.^83^

#### 3.4.10. Statistical analysis

Data analyses were carried out using GraphPad Prism software (version 10.4.1; GraphPad Software, USA). Normality of distribution was verified with the Shapiro–Wilk test before applying parametric statistics. For datasets involving more than two groups, one-way ANOVA was performed, followed by Tukey's post hoc test to adjust for multiple comparisons. Where two independent variables were considered simultaneously, two-way ANOVA was applied. Statistical significance was set at *p* < 0.05, with graded levels of significance reported as: *p* ≤ 0.05 (*), *p* ≤ 0.01 (**), *p* ≤ 0.001 (***), and *p* ≤ 0.0001 (****).

### 3.5 *In silico* toxicity and off-target profiling

The safety and off-target liabilities of compound 6 were assessed using two complementary computational platforms, pkCSM and ProTox-III. The canonical SMILES of the compound was submitted to the pkCSM server (https://biosig.lab.uq.edu.au/pkcsm/), which applies graph-based signatures and machine-learning models to predict ADMET and toxicity parameters. From the pkCSM output, the following toxicity-related indices were collected and interpreted according to standard thresholds: Ames mutagenicity, hERG I and hERG II inhibition, maximum tolerated dose in humans (log mg per kg per day), oral rat acute toxicity (LD_50_, mol kg^−1^), oral rat chronic toxicity (LOAEL, log mg per kg per day), skin sensitisation, *Tetrahymena pyriformis* toxicity (log µg L^−1^), and minnow toxicity (log mM). To complement these predictions, the compound was further evaluated using the ProTox-III platform (https://tox-new.charite.de/protox_III/), which integrates molecular similarity, fragment-based features, and pharmacophore fingerprints to classify compounds across multiple toxicity endpoints. The following categories were examined: organ toxicities (hepatotoxicity and cardiotoxicity), general toxicity endpoints (carcinogenicity, immunotoxicity, mutagenicity, cytotoxicity, blood–brain barrier permeability, and ecotoxicity), and inhibitory activity against major cytochrome P450 isoforms (CYP1A2, CYP2C19, CYP2C9, CYP2D6, CYP3A4, and CYP2E1). For each endpoint, ProTox-III reports an active/inactive classification accompanied by a prediction probability.^84,85^

## 4 Conclusion

This work reports the design, synthesis, and biological evaluation of novel 1,3-thiazolone derivatives as potential multi-target anticancer agents. Among them, compound 6 emerged as the most promising candidate, exhibiting potent and selective antiproliferative activity against breast cancer cells while sparing non-tumorigenic cells. Mechanistic investigations demonstrated that the compound induces cell cycle arrest and apoptosis, accompanied by significant modulation of oxidative stress markers, suppression of pro-inflammatory cytokine TNF-α, and inhibition of angiogenic mediator VEGFR-II. Enzyme-based assays revealed effective inhibition of aromatase, STS, and COX-2, confirming its ability to interfere with estrogen biosynthesis and inflammatory signaling pathways. These findings were further supported by *in vivo* experiments in the EAC-induced animal model, where compound 6 significantly reduced tumor burden, improved liver and kidney function markers, and preserved tissue integrity, indicating both efficacy and safety. Molecular docking and long-scale molecular dynamics simulations provided molecular-level evidence for stable and favorable interactions of compound 6 within the active sites of its biological targets, complementing the experimental findings. Collectively, these results highlight 1,3-thiazolone derivatives, particularly compound 6, as promising multi-functional lead scaffolds with cytotoxic, antioxidant, anti-inflammatory, and anti-angiogenic activities. By simultaneously modulating multiple oncogenic pathways, these compounds offer a systems-based therapeutic strategy to overcome tumor complexity and drug resistance. To fully establish its therapeutic value, future investigations should focus on disease-specific animal models, as well as pharmacokinetic, pharmacodynamic, and extended toxicity studies, to better define its translational potential in breast cancer therapy.

## Ethics approval

All experimental procedures were conducted in accordance with the guidelines of the Institutional Animal Care and Use Committee (IACUC) of Port Said University, Egypt. The study protocol was reviewed and approved by the Research Ethics Committee, Faculty of Science, Port Said University (Approval No: ERN: PSU.Sci.21). Efforts were made to minimize animal suffering and to reduce the number of animals used.

## Author contributions

Conceptualization, M. A. E., I. M. E., and E. M. S.; methodology, M. A. E., I. M. E., M. F. M. M., S. Z. A., A. A. A., and E. M. S.; software, M. A. E., M. F. M. M., S. Z. A., and E. M. S.; validation, M. A. E., I. M. E., M. F. M. M., S. Z. A., A. A. A., and E. M. S.; formal analysis, M. A. E., I. M. E., M. F. M. M., S. Z. A., and E. M. S.; investigation, M. A. E., I. M. E., and E. M. S.; resources, M. A. E., I. M. E., M. F. M. M., and E. M. S.; data curation, M. M. A. E., M. F. M. M., S. Z. A., A. A. A., and E. M. S.; writing—original draft preparation M. A. E., I. M. E., M. F. M. M., S. Z. A., A. A. A., and E. M. S.; writing—review and editing, M. A. E., I. M. E., M. F. M. M., S. Z. A., A. A. A., and E. M. S.; visualization, M. A. E., M. F. M. M., A. A. A., and E. M. S.; supervision, M. A. E., I. M. E., and E. M. S.; project administration, M. A. E., I. M. E., M. F. M. M., and E. M. S.; funding acquisition, M. A. E., I. M. E., M. F. M. M., S. Z. A., A. A. A., and E. M. S. All authors have read and agreed to the published version of the manuscript.

## Conflicts of interest

The authors affirm that they are not aware of any personal or financial conflicts that would have seemed to affect the findings of this study's research.

## Supplementary Material

RA-016-D5RA07680C-s001

## Data Availability

Data supporting the results reported in this manuscript are included in this article and as part of the supplementary information (SI). The raw data supporting the conclusions of this article will be made available by the authors without any undue reservation. Samples of final compounds are available from the authors upon request. Supplementary information is available. See DOI: https://doi.org/10.1039/d5ra07680c.
