# Supplementary material for: Design, synthesis, and multi-target anticancer evaluation of 1,3-thiazolodin-4-one analogues against breast cancer: mechanistic insights into estrogen metabolism, inflammation, angiogenesis, and oxidative stress
Source: RSC Adv. 2026 Jan 12;16(3):2528–54. doi: 10.1039/d5ra07680c (PMC12794840; doi:10.1039/d5ra07680c)
Supplement: RA-016-D5RA07680C-s001 [file RA-016-D5RA07680C-s001.pdf]

Supplementary Information (SI) for RSC Advances.
This journal is © The Royal Society of Chemistry 2025

Supporting Information

Table S1: Summary of IC_{50} values for the antiproliferative activity of the synthesized compounds (**2-7**) and doxorubicin toward MCF-7, MDA-MB 231, and MCF-10A cells.

Compd. No.	IC_{50} (µM)		
	**MCF-7**	**MDA-MB 231**	**MCF-10A**
**Compd. 2**	26.21±1.93	9.41± 0.75	--
**Compd. 3**	49.59±1.85	30.16±1.73	--
**Compd. 4**	9.672±1.14	12.55±0.87	--
**Compd. 5**	28.33±2.16	19.44±0.73	--
**Compd. 6**	6.702±0.63	2.256±0.18	36.57±1.37
**Compd. 7**	9.276±0.62	12.47±1.55	--
**Doxorubicin**	17.77±1.57	3.72±0.24	14.22 ± 0.53

Table S2. Docking results of compound **6** compared to native co-crystallized ligands with selected targets

	Native co-crystallized ligand				Compound 6		
	**Delta G**	**RMSD**	**residues**	**Interaction**	**Delta G**	**residues**	**Interaction**
**Aromatase**	-8.01	0.20	Arg115<br>Met374	H-bond<br>H-bond	-7.82	Arg115<br>Met374	H-bond<br>H-bond
**Steroid Sulfatase (STS)**	-	-	-	-	-6.81	Phe233	Pi-sulfur
**COX-1**	-7.48	0.15	Arg120<br>Tyr355	Salt bridge<br>H-bond	-6.57	Arg120<br>Tyr355	H-bond and Pi cation<br>Pi-sulfur
**COX-2**	-8.30	0.83	Gln192<br>Phe518<br>Gly526<br>Ser353	H-bond<br>H-bond<br>amide-Pi stacking<br>Pi-sigma	-6.98	Tyr355<br>Trp387	Pi-sulfur<br>Pi-sulfur
**VEGFR2**	-10.20	0.48	Glu883<br>Cys917<br>Asp1044<br>Cys1043<br>Phe916	2 H-bonds<br>2 H-bonds + Pi-sulfur<br>H-bond + Pi-anion<br>Pi-sulfur<br>Pi-Pi stacking	-7.87	Cys1043	Pi-sulfur
**TNF-α**	-6.25	0.35	Tyr59<br>Tyr151	Pi-Pi stacking<br>H-bond	-5.34	Tyr59<br>Tyr151	Pi-sulfur<br>H-bond

The redocking of co-crystallized ligands yielded RMSD values below 1.0 Å for all targets, validating the reliability of the docking protocol (Table S1). The hit compound demonstrated favorable binding energies and significant molecular interactions across all tested targets. Among these, the strongest binding affinity was observed with VEGFR2 (−7.87 kcal/mol), followed by Aromatase (−7.82 kcal/mol), and COX-2 (−6.98 kcal/mol). In contrast, the weakest interaction was recorded with TNF-α (−5.34 kcal/mol).

Table S3. pkCSM-predicted toxicity parameters for compound **6**, summarizing genotoxicity, cardiotoxicity (hERG I/II), skin sensitization, systemic tolerance (MTD), acute and chronic toxicity estimates (LD_{50} and LOAEL), and environmental toxicity indices (T. pyriformis and minnow toxicity).

Toxicity Parameter	Prediction / Value	Type	Interpretation
**Ames mutagenicity**	No	Categorical (Yes/No)	Non-mutagenic
**hERG I inhibitor**	No	Categorical (Yes/No)	No predicted cardiotoxicity
**hERG II inhibitor**	No	Categorical (Yes/No)	No predicted cardiotoxicity
**Maximum tolerated dose (human)**	0.265 log mg/kg/day	Numeric	Moderate tolerance in humans
**Oral Rat Acute Toxicity (LD_{50})**	2.917 mol/kg	Numeric	Low acute toxicity
**Oral Rat Chronic Toxicity (LOAEL)**	1.214 log mg/kg_bw/day	Numeric	Low chronic toxicity
**Skin Sensitisation**	No	Categorical (Yes/No)	Non-sensitizing
***T. pyriformis* toxicity**	0.504 log µg/L	Numeric	Minimal aquatic toxicity
**Minnow toxicity**	0.717 log mM	Numeric	Minimal ecotoxicity

Table S4. ProTox-III–predicted toxicity endpoints for compound **6**, including organ toxicity, carcinogenicity, mutagenicity, immunotoxicity, cytotoxicity, BBB penetration, ecotoxicity, and inhibitory activity toward major CYP450 enzymes.

Category	Endpoint	Shorthand	Prediction	Probability	Interpretation
**Organ Toxicity**	Hepatotoxicity	DILI	Inactive	0.59	No predicted liver toxicity
	Cardiotoxicity	CARDIO	Inactive	0.60	No predicted heart toxicity
**Toxicity Endpoints**	Carcinogenicity	CARCINO	Inactive	0.60	Non-carcinogenic
	Immunotoxicity	IMMUNO	Active	0.53	Possible mild IM effect
	Mutagenicity	MUTAGEN	Inactive	0.57	Non-mutagenic
	Cytotoxicity	CYTO	Inactive	0.68	Non-cytotoxic to normal cells
	BBB-barrier	BBB	Inactive	0.55	Limited CNS penetration
	Ecotoxicity	ECO	Inactive	0.59	Non-ecotoxic
**Metabolism (CYP Enzymes)**	CYP1A2	CYP1A2	Inactive	0.94	No inhibition predicted
	CYP2C19	CYP2C19	Inactive	0.78	No inhibition predicted
	CYP2C9	CYP2C9	Inactive	0.64	No inhibition predicted
	CYP2D6	CYP2D6	Inactive	0.69	No inhibition predicted
	CYP3A4	CYP3A4	Inactive	0.80	No inhibition predicted
	CYP2E1	CYP2E1	Inactive	0.99	No inhibition predicted

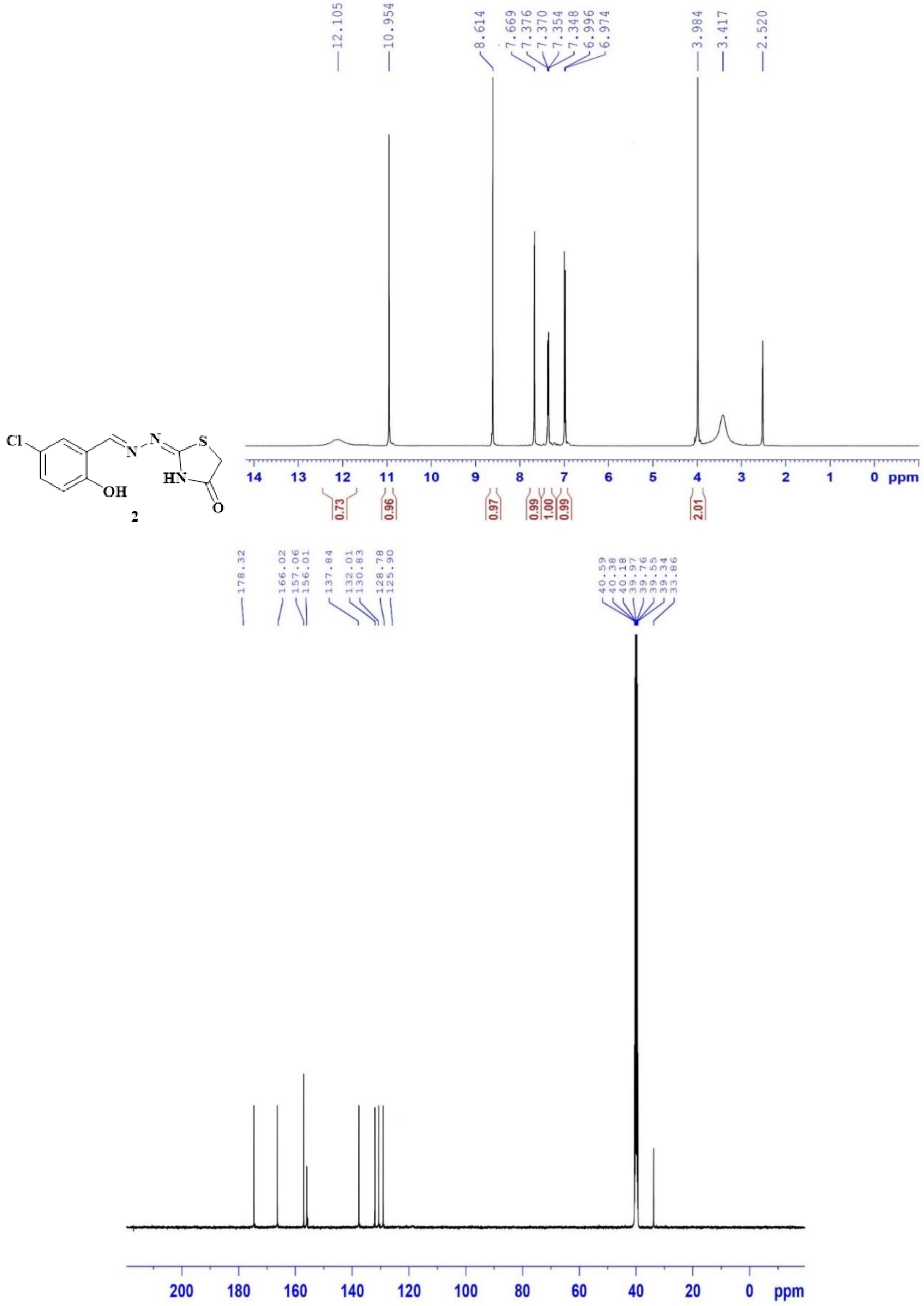

Figure S1A: ^{1}H- and ^{13}C-NMR Spectrum of Compound **2**

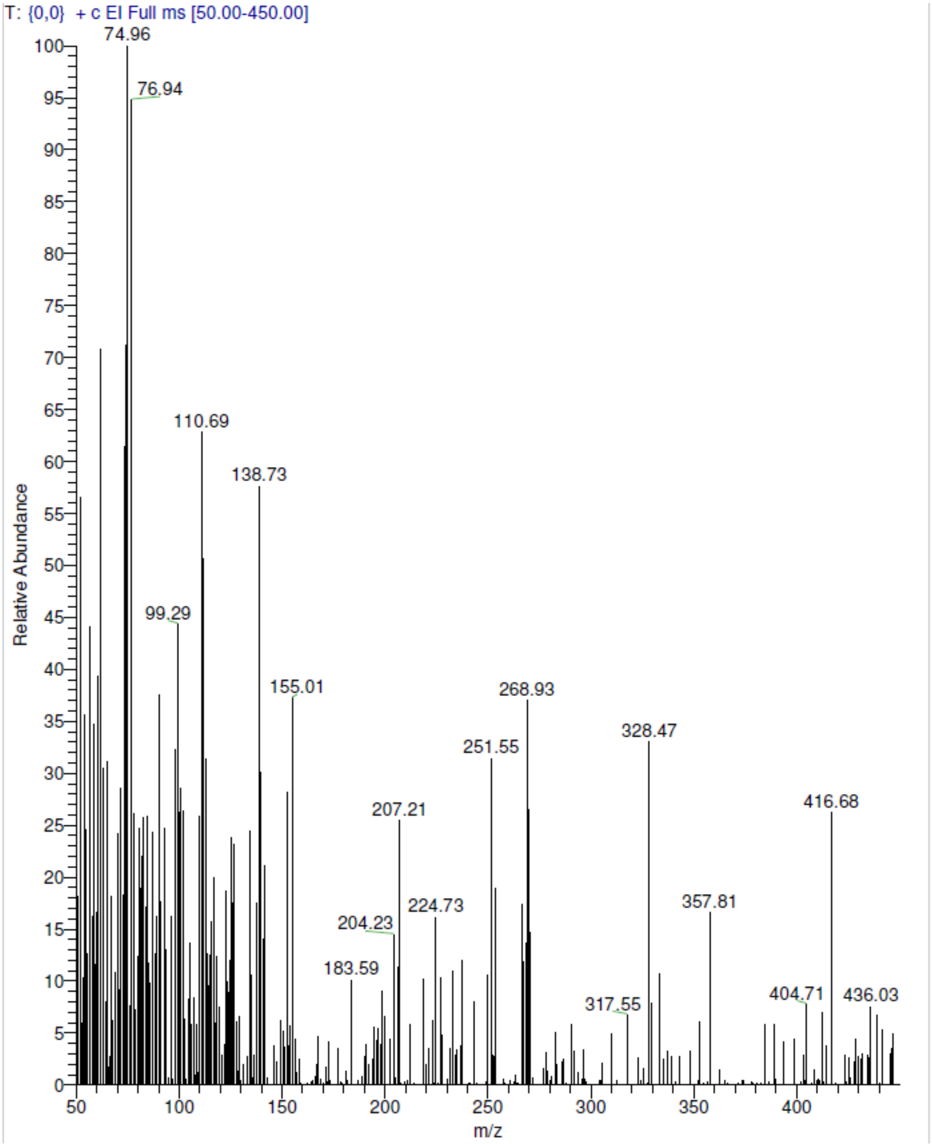

Figure S1B: ESI-MS analysis of Compound **2**

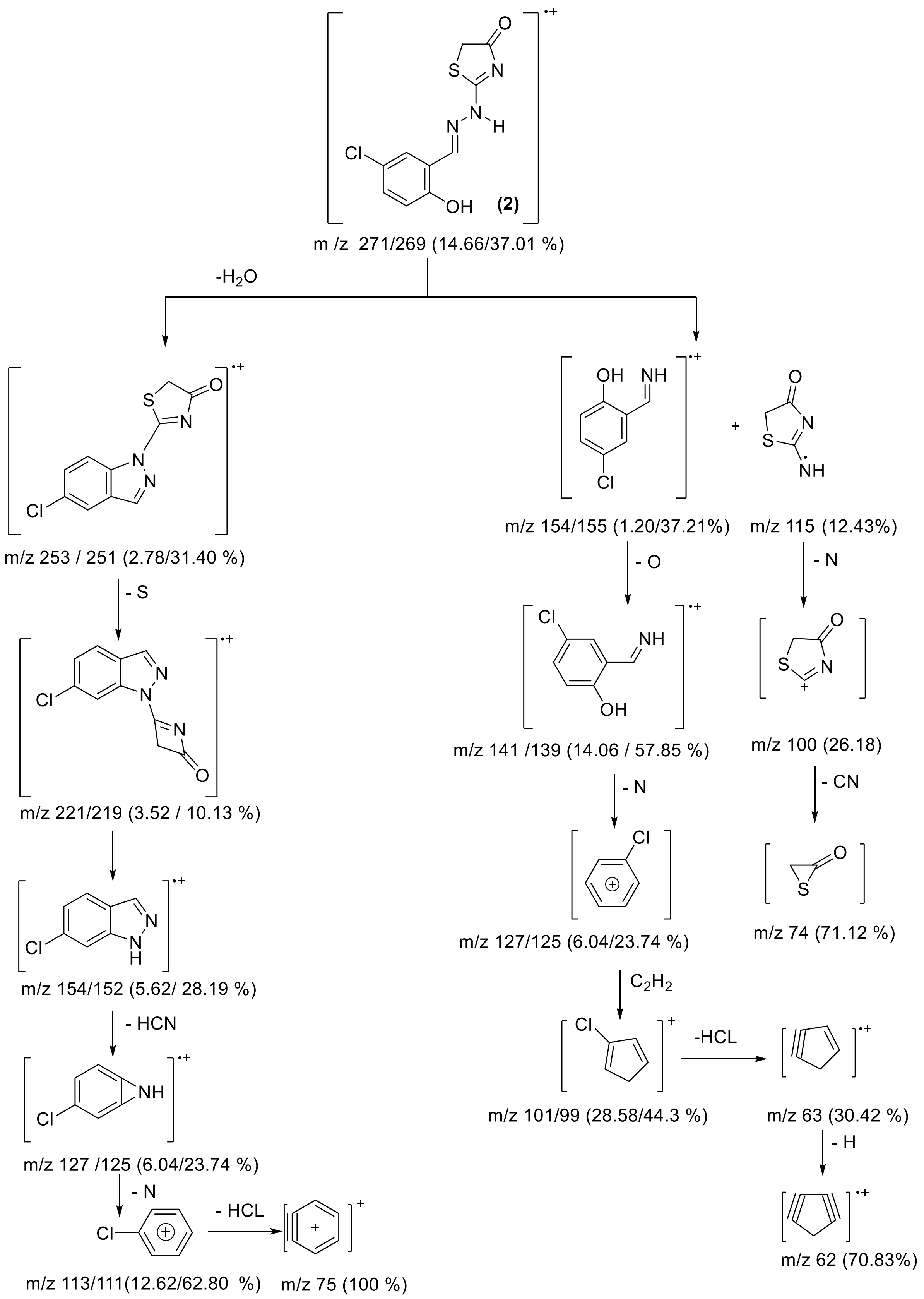

Figure S1C: Mass fragmentation pattern of Compound **2**

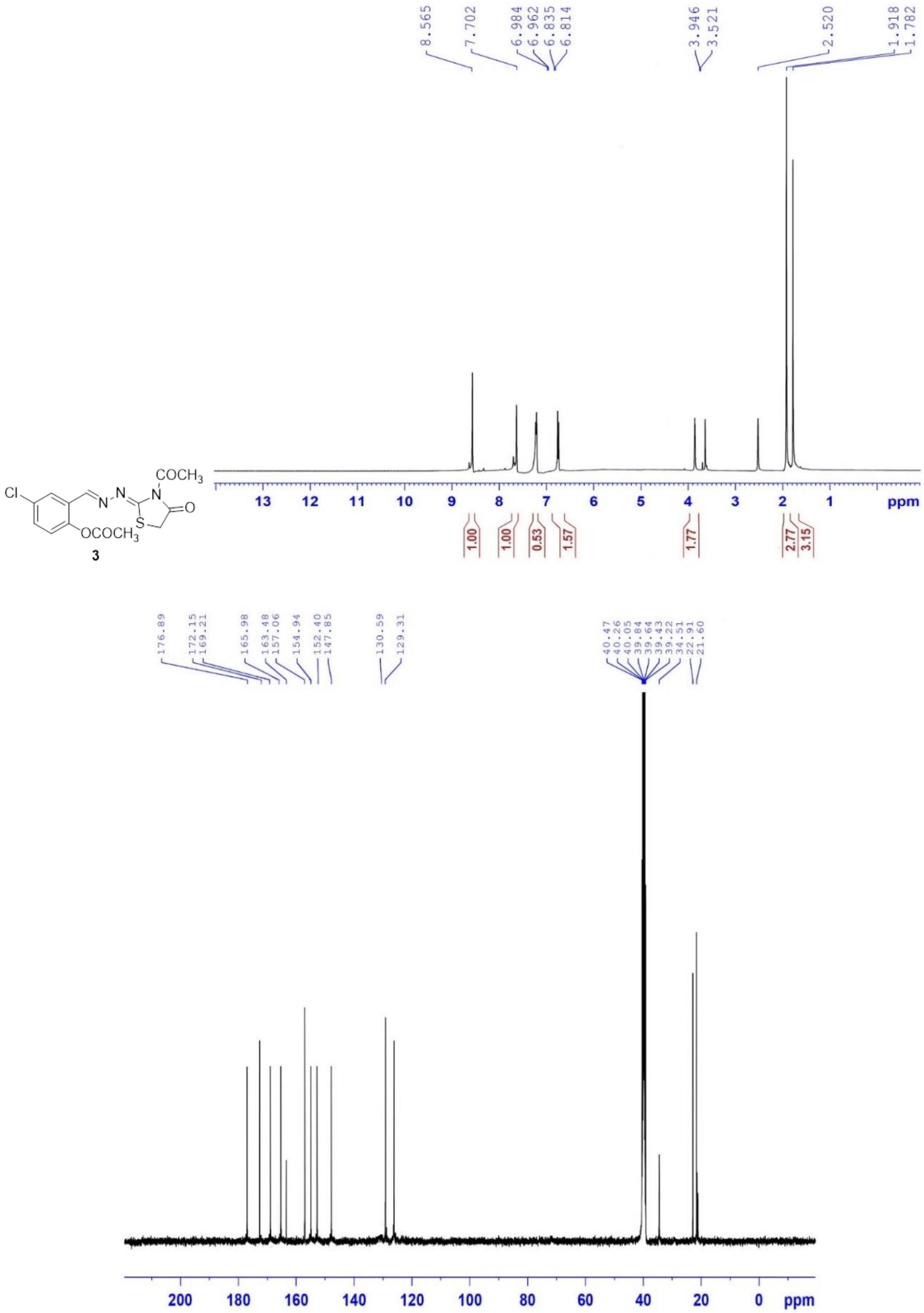

Figure S2A: ^{1}H- and ^{13}C-NMR Spectrum of Compound **3**

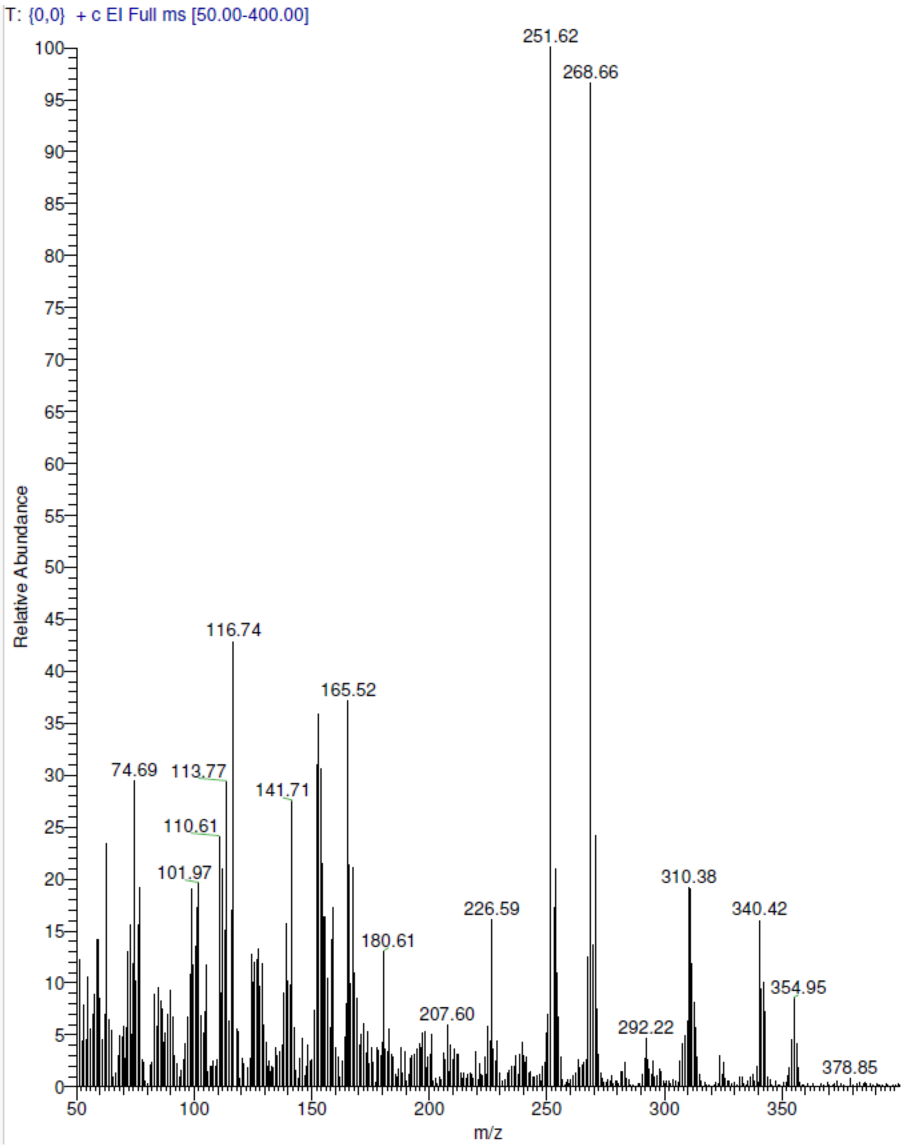

Figure S2B: ESI-MS analysis of Compound **3**

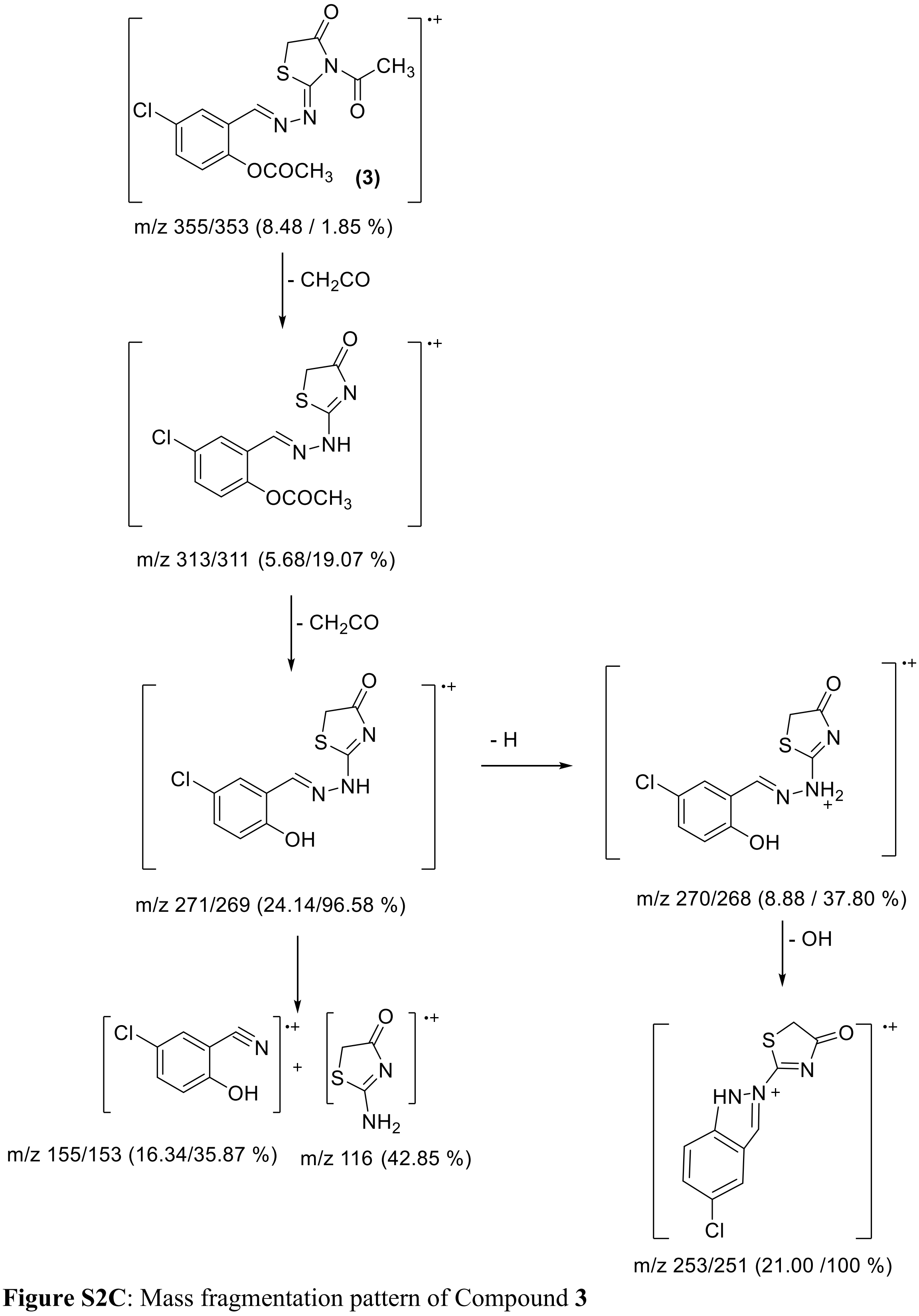

Figure S2C: Mass fragmentation pattern of Compound **3**

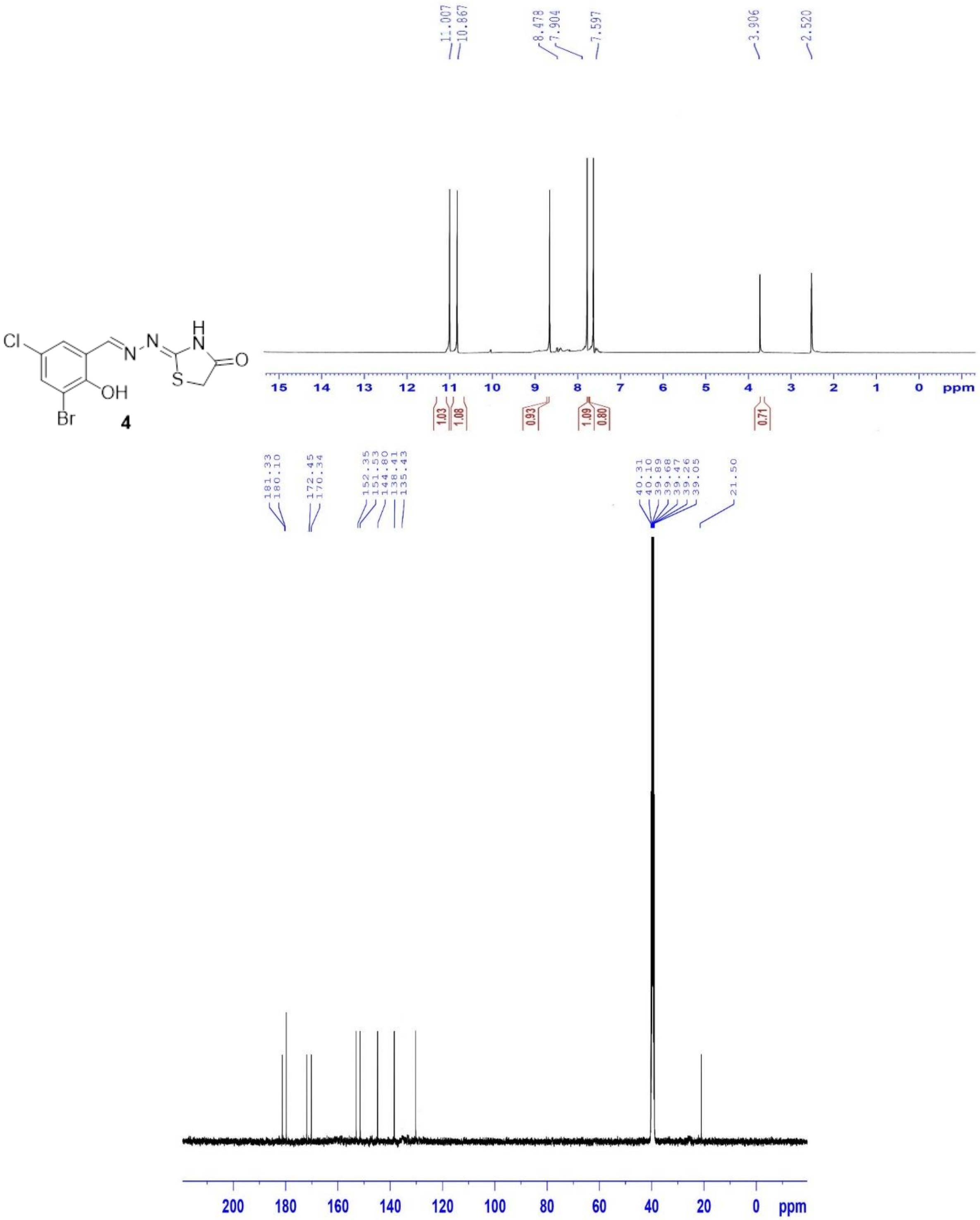

Figure S3: ^{1}H- and ^{13}C-NMR Spectrum of compound **4**

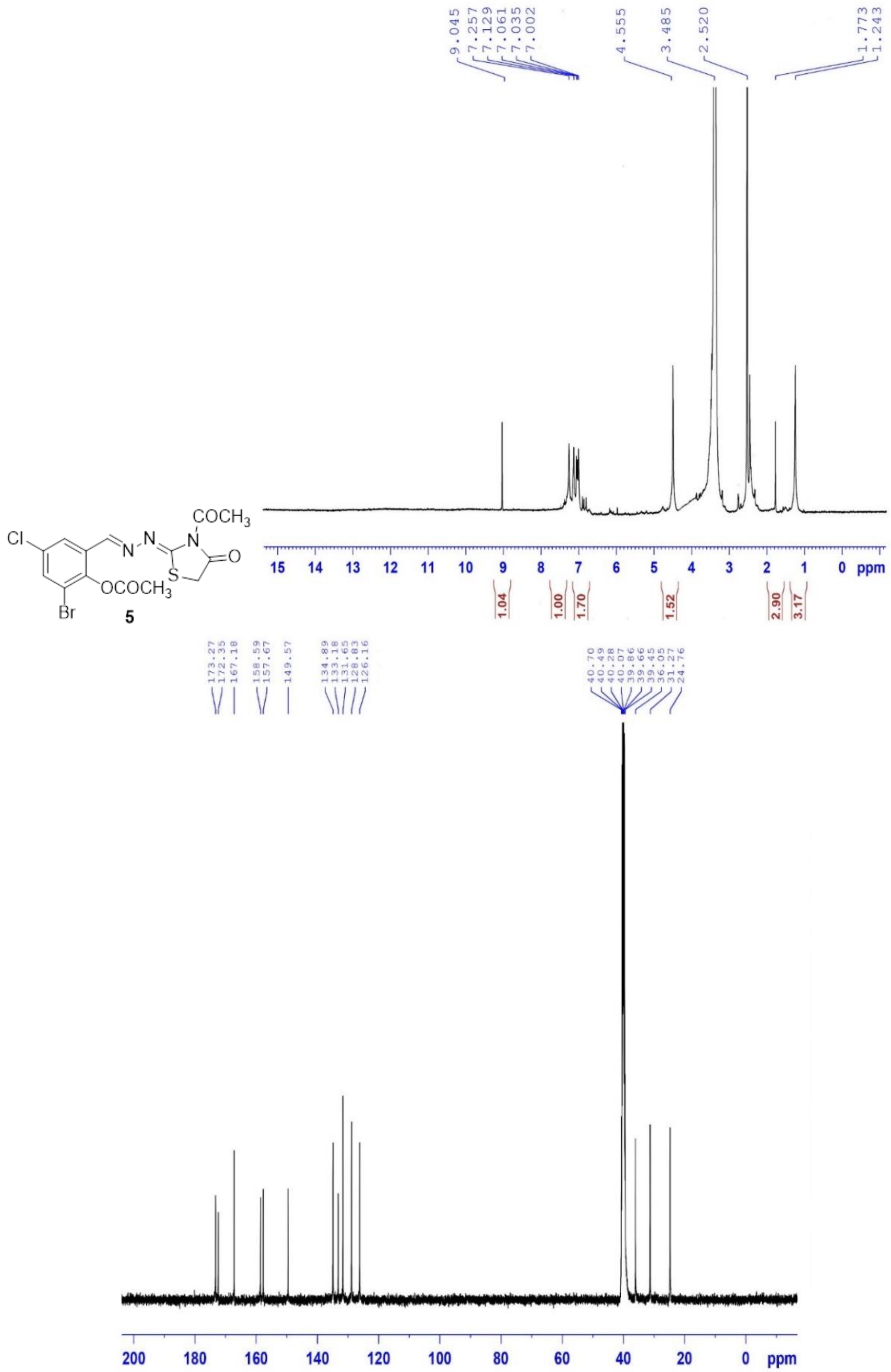

Figure S4: ^{1}H- and ^{13}C-NMR Spectrum of Compound **5**

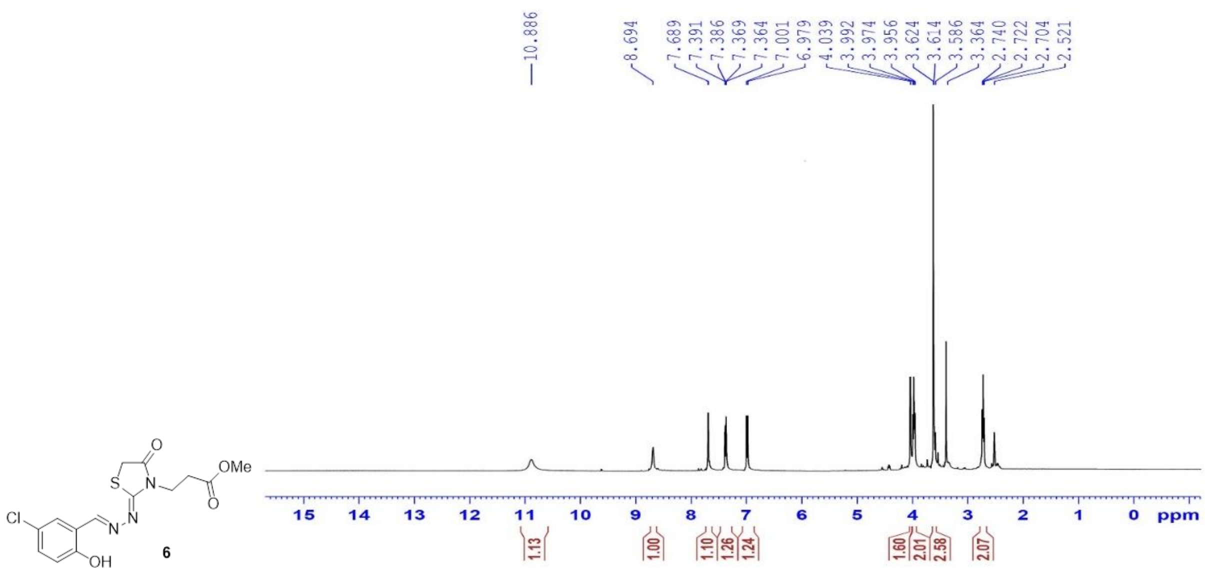

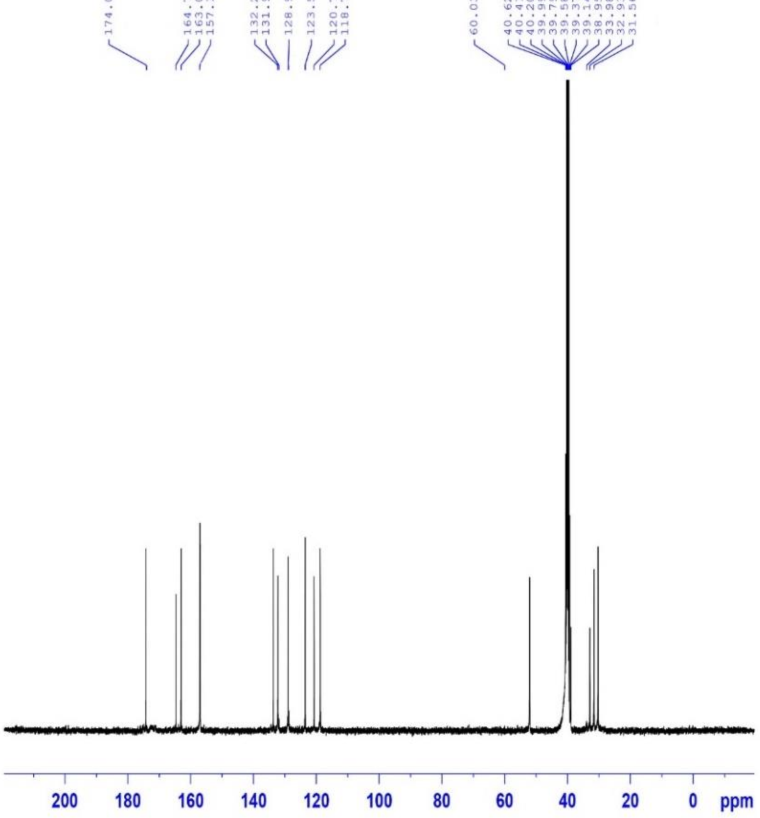

Figure S5: ^{1}H- and ^{13}C-NMR Spectrum of Compound **6**

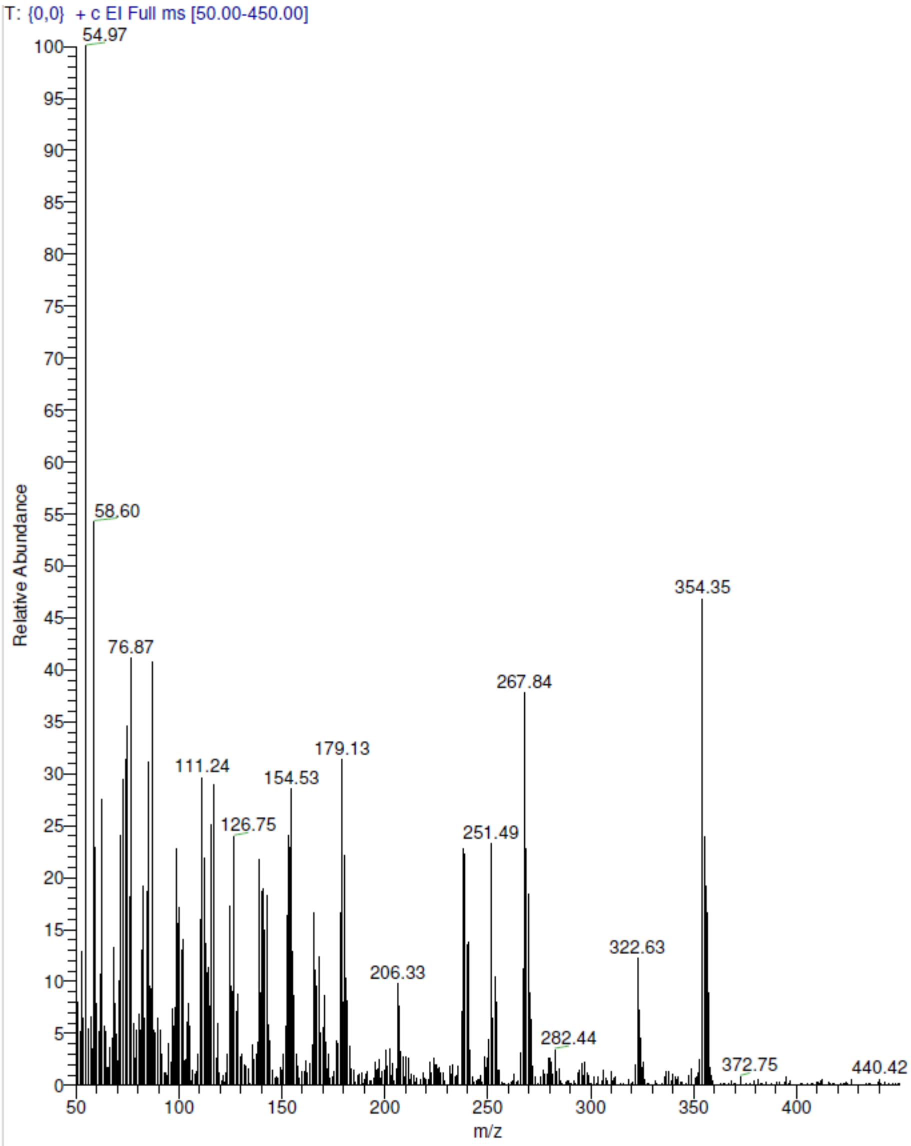

Figure S5B: ESI-MS analysis of Compound **6**

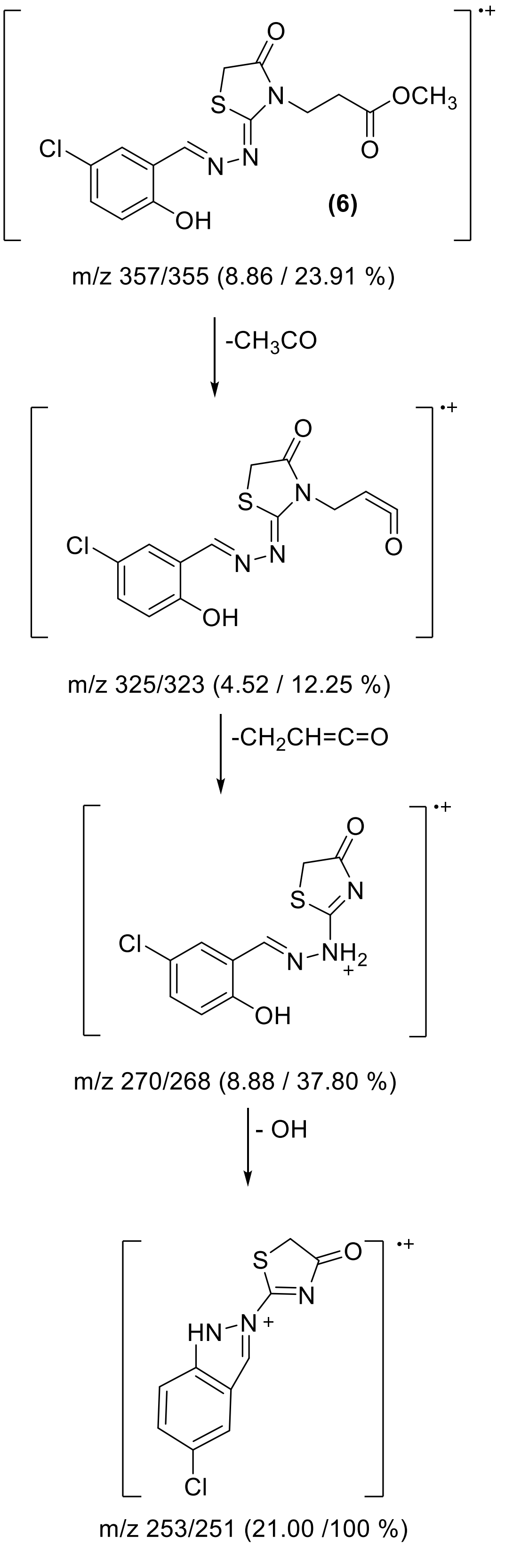

Figure S5C: Mass fragmentation pattern of Compound **6**

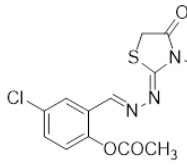

7

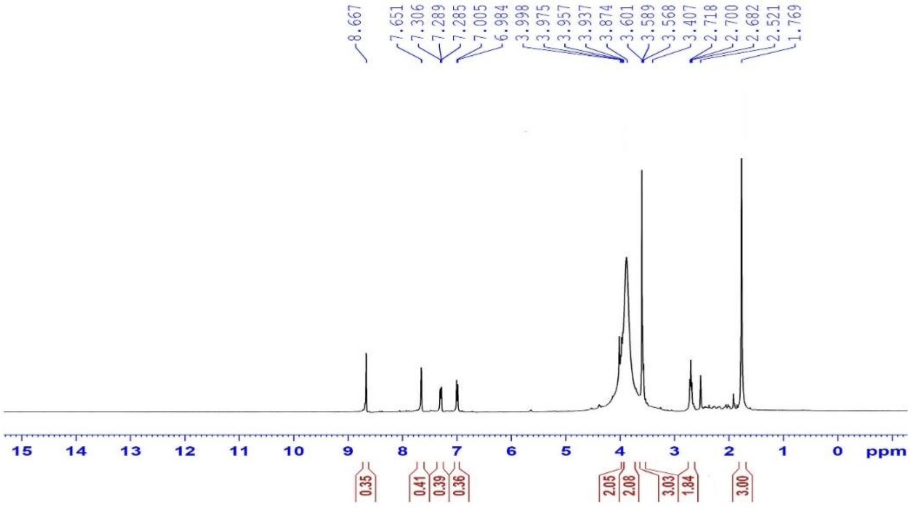

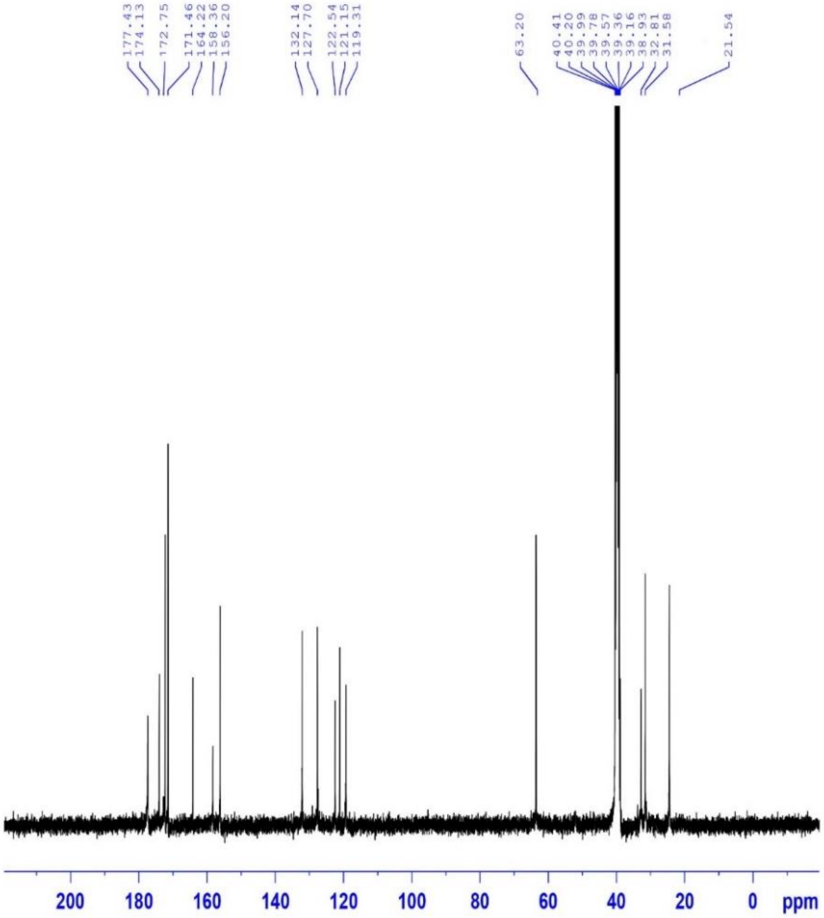

Figure S6: ^{1}H- and ^{13}C-NMR Spectrum of Compound **7**

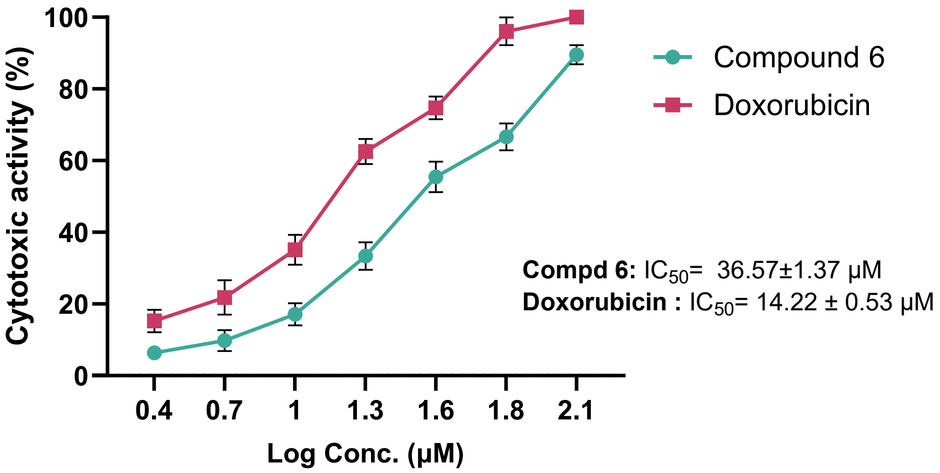

Figure S7: Effect of compound **6** and doxorubicin against non-tumorigenic MCF-10A cells as assessed by MTT assay after incubation for 48h at different concentrations.

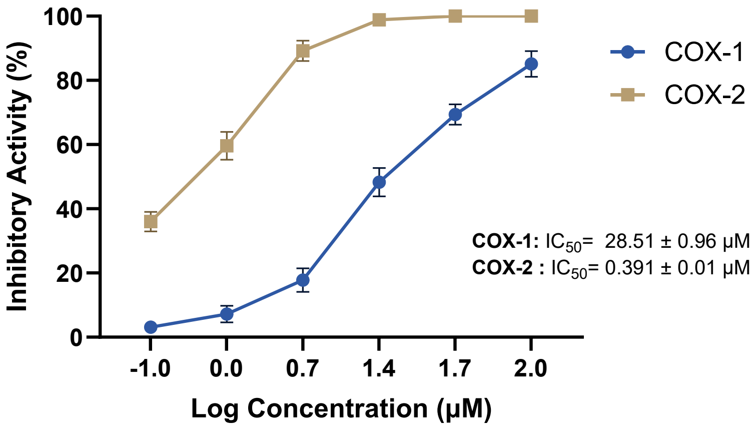

Figure S8: Dose-response inhibitory potential of celecoxib toward the activity of COX-1 and COX-2 proteins. Data expressed as mean±SE from three independent experiments (n = 3).

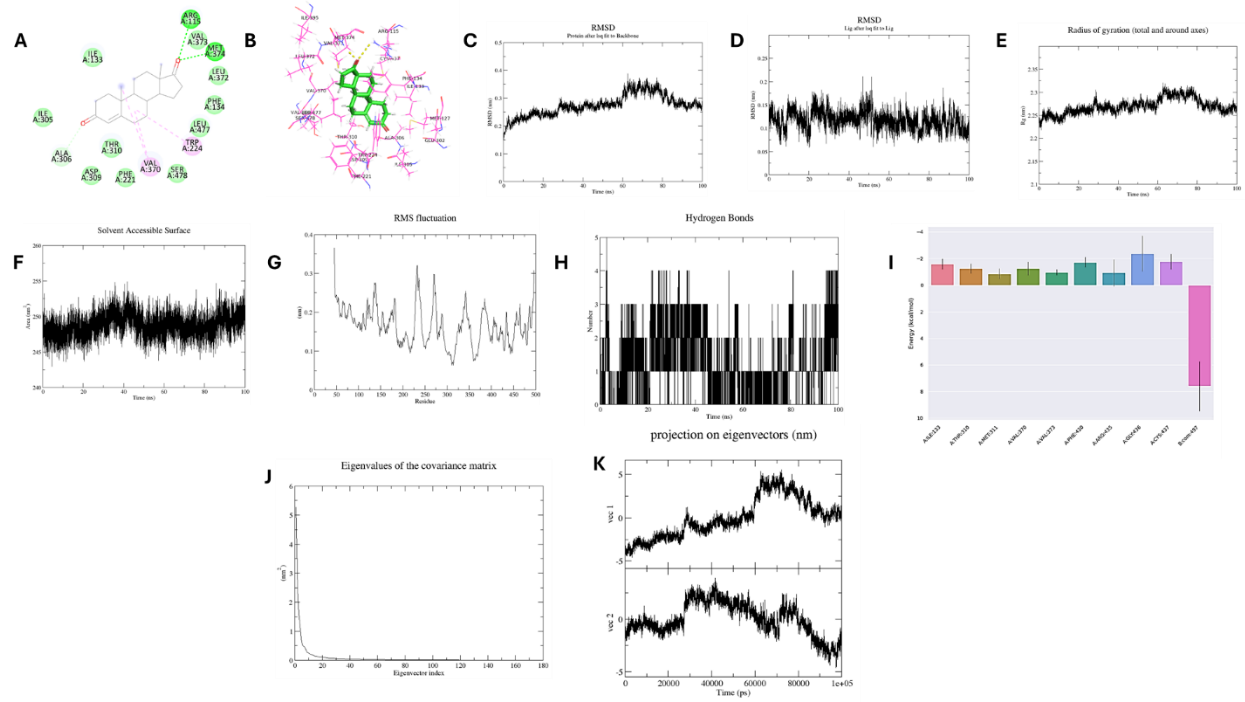

Figure S9: Molecular modelling, molecular dynamics, and principal component analysis of docked ligands with aromatase protein (PDB ID: *3EQM*). (A, B) 2D and 3D interaction diagrams of the co-crystallized ligand with the aromatase protein. **(C-E)** Structural dynamics of aromatase protein calculated during the 100 ns of MD trajectories; Root Mean Square deviation (RMSD) of aromatase (**C**), Ligand RMSD (**D**), Radius of gyration (**E**), complexes SASA values (**F**); Root Mean Square fluctuation (RMSF) of protein backbone (**G**), number of H-bonds formed with compound **6** (**H**), and Residue-binding-free energy decomposition for the simulated complex (**E**). (**J-K**) Principal component analysis and free energy landscape of the Aromatase–compound **6** complex**. (J)** Eigenvalue distribution of the covariance matrix, illustrating the dominant modes of motion. **(K)** Time evolution of the projections along PC1 and PC2 over the 100-ns trajectory.

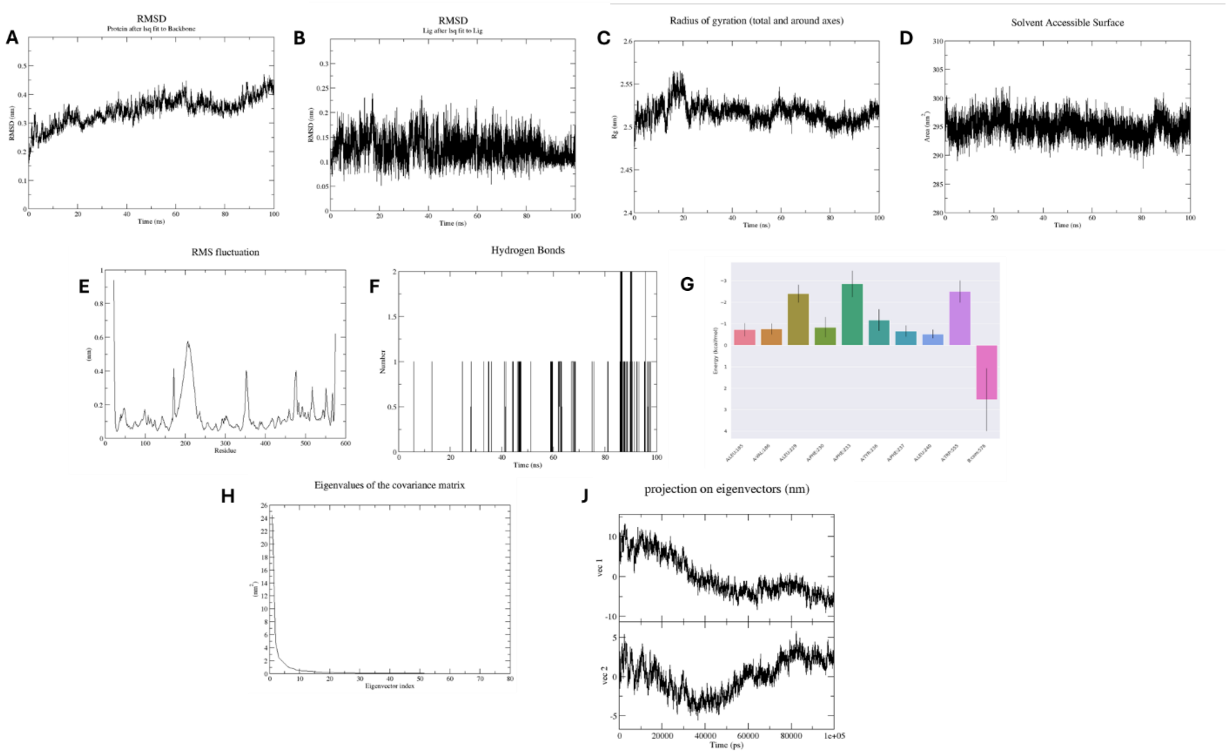

Figure S10: Molecular dynamics analysis of compound 6 with Steroid Sulfatase (STS) protein (PDB ID: *1P49*). (A-G) Structural dynamics of STS protein calculated during the 100 ns of MD trajectories; Root Mean Square deviation (RMSD) of STS (**A**), Ligand RMSD (**B**), Radius of gyration (**C**), complexes SASA values (**D**); Root Mean Square fluctuation (RMSF) of protein backbone (**E**), number of H-bonds formed with compound **6** (**F**), and Residue-binding-free energy decomposition for the simulated complex (**G**). (**H-I**) Principal component analysis and free energy landscape of STS–compound **6** complex**. (H)** Eigenvalue distribution of the covariance matrix, illustrating the dominant modes of motion. **(I)** Time evolution of the projections along PC1 and PC2 over the 100-ns trajectory.

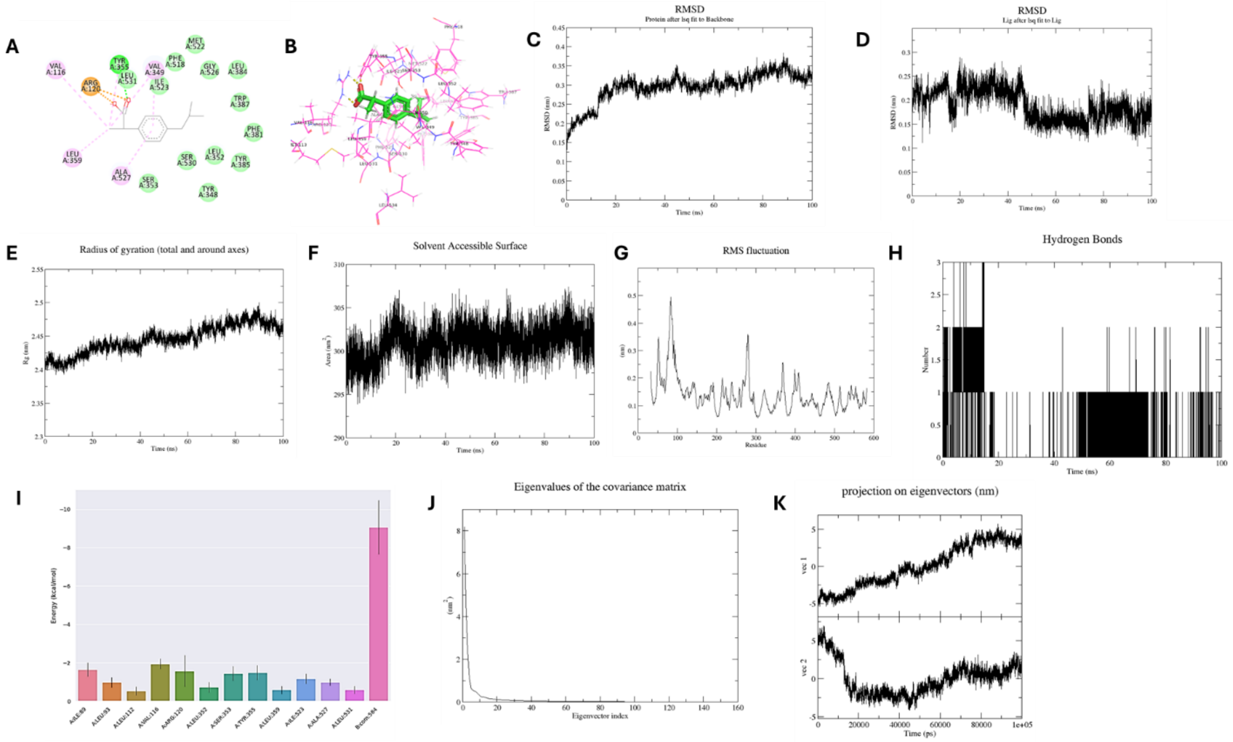

Figure S11: Molecular modelling and molecular dynamics analysis of docked ligands with cyclooxygenase-1 (COX-1) protein (PDB ID: *1EQG*). (A, B) 2D and 3D interaction diagrams of the co-crystallized ligand with COX-1 protein. **(C-I)** Structural dynamics of COX-1 protein calculated during the 100 ns of MD trajectories; Root Mean Square deviation (RMSD) of COX-1 (**C**), Ligand RMSD (**D**), Radius of gyration (**E**), complexes SASA values (**F**); Root Mean Square fluctuation (RMSF) of protein backbone (**G**), number of H-bonds formed with compound **6** (**H**), and Residue-binding-free energy decomposition for the simulated complex (**I**). (**J-K**) Principal component analysis and free energy landscape of COX-1–compound **6** complex**. (J)** Eigenvalue distribution of the covariance matrix, illustrating the dominant modes of motion. **(K)** Time evolution of the projections along PC1 and PC2 over the 100-ns trajectory.

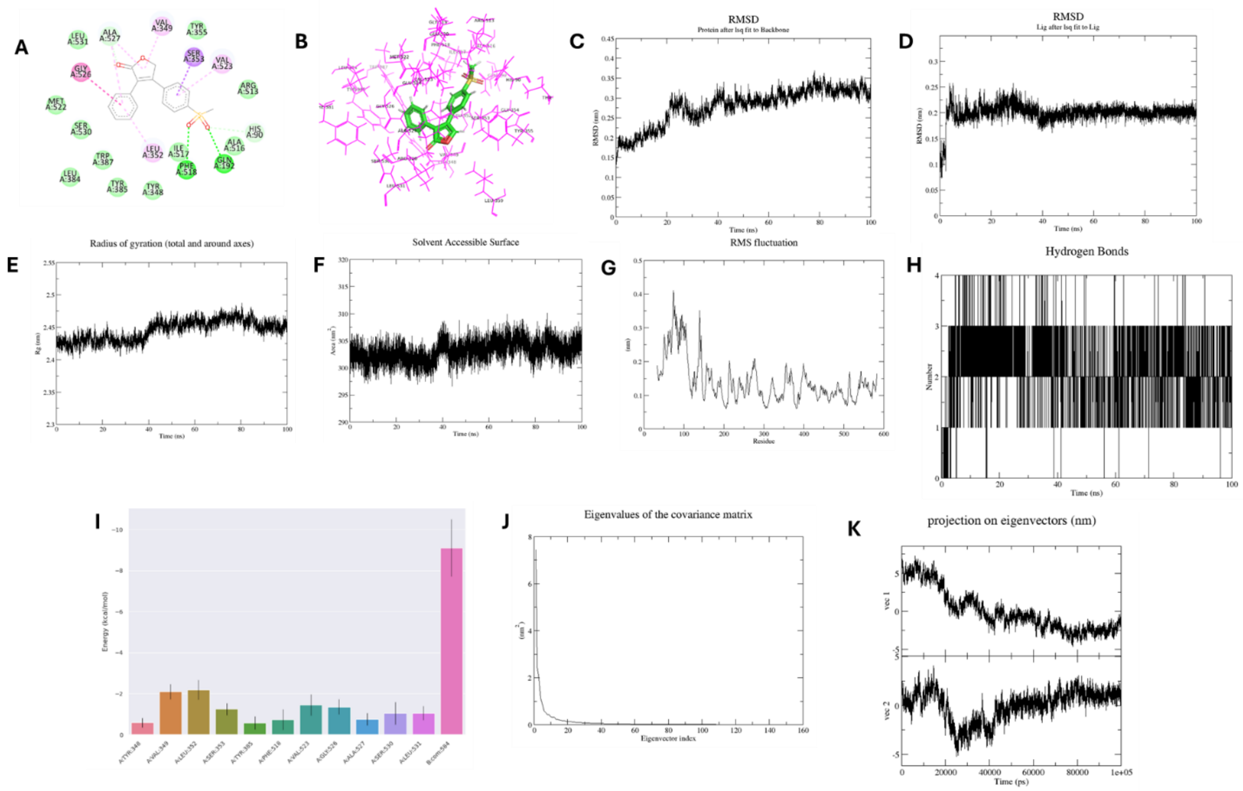

Figure S12: Molecular modelling and molecular dynamics analysis of docked ligands with cyclooxygenase-2 (COX-2) protein (PDB ID: *5KIR*). (A, B) 2D and 3D interaction diagrams of the co-crystallized ligand with COX-2 protein. **(C-I)** Structural dynamics of COX-2 protein calculated during the 100 ns of MD trajectories; Root Mean Square deviation (RMSD) of COX-2 (**C**), Ligand RMSD (**D**), Radius of gyration (**E**), complexes SASA values (**F**); Root Mean Square fluctuation (RMSF) of protein backbone (**G**), and number of H-bonds formed with compound **6** (**H**), and Residue-binding-free energy decomposition for the simulated complex (**I**). (**J-K**) Principal component analysis and free energy landscape of COX-2–compound **6** complex**. (J)** Eigenvalue distribution of the covariance matrix, illustrating the dominant modes of motion. **(K)** Time evolution of the projections along PC1 and PC2 over the 100-ns trajectory.

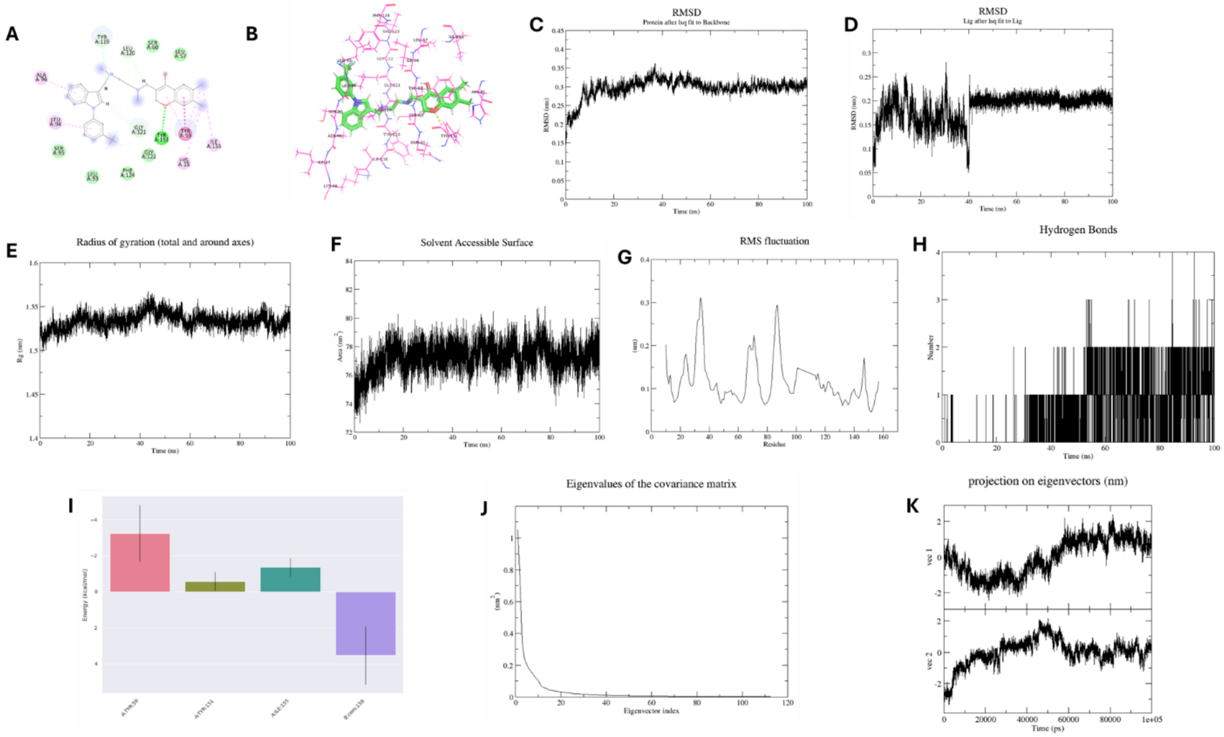

Figure S13: Molecular modelling and molecular dynamics analysis of docked ligands with tumor necrosis factor-alpha (TNF-α) protein (PDB ID: *2AZ5*). (A, B) 2D and 3D interaction diagrams of the co-crystallized ligand with TNF-α protein. **(C-H)** Structural dynamics of TNF-α protein calculated during the 100 ns of MD trajectories; Root Mean Square deviation (RMSD) of TNF-α (**C**), Ligand RMSD (**D**), Radius of gyration (**E**), complexes SASA values (**F**); Root Mean Square fluctuation (RMSF) of protein backbone (**G**), number of H-bonds formed with compound **6** (**H**), and Residue-binding-free energy decomposition for the simulated complex (**I**). (**J-K**) Principal component analysis and free energy landscape of TNF-α–compound **6** complex**. (J)** Eigenvalue distribution of the covariance matrix, illustrating the dominant modes of motion. **(K)** Time evolution of the projections along PC1 and PC2 over the 100-ns trajectory.

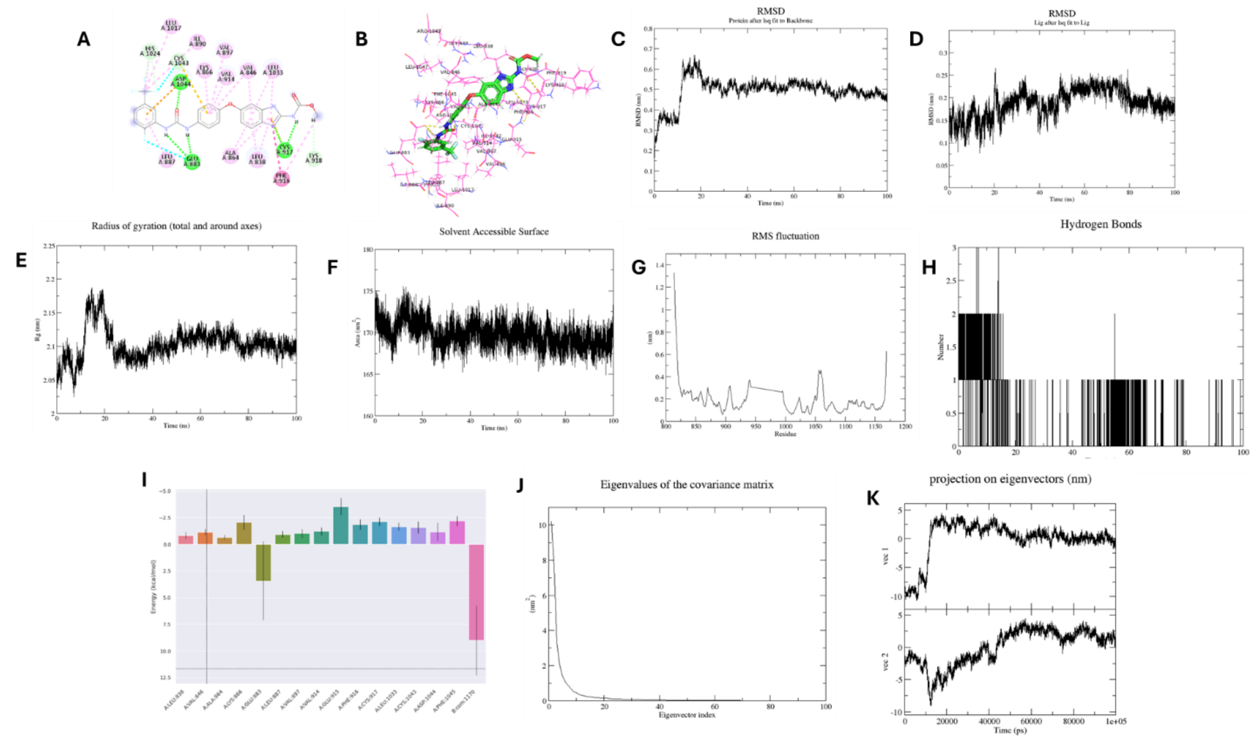

Figure S14: Molecular modelling and molecular dynamics analysis of docked ligands with vascular endothelial growth factor receptor 2 (VEGFR2) protein (PDB ID: *2OH4*). (A, B) 2D and 3D interaction diagrams of the co-crystallized ligand with VEGFR2 protein. **(C-H)** Structural dynamics of VEGFR2 protein calculated during the 100 ns of MD trajectories; Root Mean Square deviation (RMSD) of VEGFR2 (**C**), Ligand RMSD (**D**), Radius of gyration (**E**), complexes SASA values (**F**); Root Mean Square fluctuation (RMSF) of protein backbone (**G**), number of H-bonds formed with compound **6** (**H**), and Residue-binding-free energy decomposition for the simulated complex (**I**). (**J-K**) Principal component analysis and free energy landscape of VEGFR2–compound **6** complex**. (J)** Eigenvalue distribution of the covariance matrix, illustrating the dominant modes of motion. **(K)** Time evolution of the projections along PC1 and PC2 over the 100-ns trajectory.Supplementary
